# Social determinants of dementia: A scoping review

**DOI:** 10.1002/alz.70524

**Published:** 2025-07-28

**Authors:** Sebastian Walsh, Matthias Klee, Esther K. Hui, Usman Saeed, Sheena Waters, Isla Kuhn, Joyce Siette, Vasiliki Orgeta, Jean Stafford, Laura J. Smith, Stefano Tamburin, Daria E. A. Jensen, Elisa Mantovani, Avinash Chandra, Sarah‐Naomi James, Eugene Y. H. Tang, David J. Llewellyn, Isabelle F. Foote, Scott T. Chiesa, Anouk F. J. Geraets, the DEMON SDOH International Research Group

**Affiliations:** ^1^ Cambridge Public Health University of Cambridge, Forvie Site Cambridge UK; ^2^ Department of General Internal Medicine and Psychosomatics University Medical Centre Heidelberg Heidelberg Germany; ^3^ Division of Psychiatry University College London London UK; ^4^ Institute of Medical Science Temerty Faculty of Medicine University of Toronto Toronto Ontario Canada; ^5^ Hurvitz Brain Sciences Program Sunnybrook Research Institute Toronto Ontario Canada; ^6^ Centre for Preventive Neurology Queen Mary University of London London UK; ^7^ The MARCS Institute for Brain Behaviour and Development Western Sydney University Westmead NSW Australia; ^8^ Advanced Care Research Centre University of Edinburgh Edinburgh UK; ^9^ Department of Neurosciences Biomedicine and Movement Sciences University of Verona Verona Italy; ^10^ Clinic of Cognitive Neurology University Medical Center Leipzig Leipzig Germany; ^11^ Department of Neurology Max Planck Institute for Human Cognitive and Brain Sciences Leipzig Germany; ^12^ Institute of Cardiovascular Science University College London London UK; ^13^ Population Health Sciences Institute Newcastle University Newcastle upon Tyne UK; ^14^ Department of Health and Community Sciences University of Exeter Medical School, St Luke's Campus Exeter UK; ^15^ Institute for Behavioral Genetics University of Colorado Boulder Colorado USA; ^16^ Department of Social Sciences University of Luxembourg Esch‐sur‐Alzette Luxembourg

**Keywords:** dementia, employment, ethnicity, food environment, geography, housing, income, inequalities, physical environment, pollution, poverty, review, social determinants, social inclusion, socioeconomic status

## Abstract

Dementia risk is influenced by the social conditions in which people live. These social determinants of dementia (SDOD) are potential targets for prevention. We conducted a comprehensive scoping review to identify current evidence on SDOD in two stages: (1) review of systematic reviews on SDOD; and (2) review of primary literature to address identified gaps in the evidence base. Of the 3445 articles screened, 26 reviews and 74 primary studies were included. Evidence from reviews provide clear and consistent patterns for some SDOD (e.g., education, air pollution, socioeconomic status, ethnicity), while evidence for others is still emerging (e.g., housing quality/stability), or lacking entirely (e.g., incarceration). SDOD are important over the life course. Additional evidence is needed for understudied domains and to unravel the complex interactions between determinants, whilst education and air pollution stand out as key targets for public health interventions.

**Highlights:**

- There is clear and consistent evidence for some social determinants of dementia (SDOD) across diverse domains.
- Evidence for other SDOD (e.g., housing, incarceration) is still emerging or lacking.
- Evidence for SDOD comes mainly from high‐income countries.
- The complex intercorrelations between SDODs demand a nuanced analytical approach.
- Standardized measures and longer follow‐up to study SDOD are recommended.

## 1 INTRODUCTION

Dementia is among the most significant contributors to health and socioeconomic costs worldwide.^1^, ^2^ As the global population ages, the prevalence of dementia is forecast to increase, with low‐and middle‐income countries (LMICs) bearing the greatest burden.^2^ Inequalities also exist within countries, with higher dementia rates and earlier age of disease onset disproportionately affecting those with fewer socioeconomic resources.^3^, ^4^

Epidemiological studies have reported decreasing age‐specific incidence of dementia in high‐income countries (HICs) of around 13% per decade.^5^, ^6^ These secular trends imply that risk reduction is possible, and are thought to be secondary to improved public health, for example reduced smoking rates, and social factors, for example higher rates of education.^5^, ^6^ Most research on dementia prevention has been focused on individual‐level interventions^7^—those which are focused on encouraging individuals to lower their risk through lifestyle change or adherence to medications. However, population‐level interventions—those which focus on changing social conditions and environments to lower risk across the population—may be more effective and may help to reduce inequalities.^8^, ^9^ In addition, most research on dementia prevention is conducted in HICs. Therefore, an inclusive population‐level dementia prevention approach is a public health priority.^10^

Several potentially modifiable risk factors for dementia, such as hypertension, smoking, and midlife obesity, have been identified at different stages of the life course, and causality has been asserted by expert groups.^11^, ^12^ These risk factors follow a socioeconomic gradient, with a higher clustering of risk in resource‐constrained and socially deprived settings.^13^ For instance, an individual living in an affluent area is more likely to be able to access and afford healthy foods and achieve a higher level of education compared to an individual living in a resource‐deprived area. The conditions in which individuals live, work, and age can significantly influence disparities in dementia risk. These environmental conditions, the “social determinants of health,” have a key role in shaping health outcomes, including dementia risk.^3^, ^4^, ^11^ By systematically identifying and addressing these determinants in relation to dementia risk, it may be possible to lower dementia risk in a manner that is both impactful and equitable across populations.^14^, ^15^

Researching social determinants of health is complex because of the broad range and interacting nature of environmental and socioeconomic factors that constitute social environments. In addition, some social determinants can be challenging to assess due to their complex nature (e.g., discrimination). As the social determinants of dementia are still unclear, we undertook a comprehensive and wide‐ranging scoping review to systematically and pragmatically capture the present state of evidence linking social determinants of health to incident dementia.^16^, ^17^, ^18^ Consequently, more specific research questions can be answered in future narrower systematic reviews.

The overarching aim of this scoping review is to guide this emerging field of research on social determinants of dementia (SDOD) by providing an overview of existing research, identifying gaps, and presenting a series of actionable research recommendations for future studies.

## 2 METHODS

The protocol for this scoping review was registered on the Open Science Framework (https://doi.org/10.17605/OSF.IO/SK69J).^19^ We have reported the results against the Preferred Reporting Items for Systematic reviews and Meta‐Analyses (PRISMA) Scoping Review extension checklist (see Supplementary Material).

### 2.1 Review definition

We defined SDOD as: “conditions, beyond medical, demographic, and individual lifestyle factors, which may influence people's dementia risk through their life.” This definition was developed through a consensus agreement by the DEMON (DEep deMentia phenOtypiNg) Network Social Determinants of Health international research group.^20^ It builds upon existing definitions of social determinants of health in the literature^15^, ^21^ as well as from leading international public health agencies, such as the World Health Organization (see Table S1 for full details).^11^, ^22^, ^23^

### 2.2 Review stages

Pilot searches indicated that a search strategy accommodating all primary literature investigating the associations between social determinants of health and incident dementia (> 50,000 search hits) would not be feasible. We therefore conducted a scoping review in two stages: (1) a review of published systematic reviews on social determinants of health and incident dementia, including the Lancet Commission reports on dementia;^11^, ^22^, ^23^ and (2) a review of primary literature on social determinants of health and incident dementia using a search strategy designed to update or fill gaps identified by the first stage. Searches were designed by an expert librarian (I.K.), building upon examples of social determinants of health from existing literature (Table S1), and conducted without language restrictions.

#### 2.2.1 Review of reviews

The Lancet Commission on dementia prevention, intervention, and care has published three reports.^11^, ^22^, ^23^ These reports summarised results from systematic reviews and meta‐analyses of associations, as well as specific additional studies on genetic epidemiology, mechanistic evidence, and interventional evidence. Although modifiable risk factors were predominantly reviewed through an individual‐level paradigm (e.g., hypertension, physical activity), the reports have also comprehensively presented evidence related to some SDOD, such as education, air pollution, and inequalities. The first report was published in 2017^22^ and was updated in 2020^23^ and 2024.^11^

We then searched for systematic reviews that examined evidence linking specific social determinants of health, not comprehensively covered in the Lancet Commission reports, to dementia risk. We searched Medline via Ovid on the 22nd of February 2024, using terms for “dementia” and “*social determinants of health*,” and “*review*” without date restrictions (see Table S2 for the full search strategy). We included systematic reviews that were conducted using a clearly reported systematic literature search and reported on a specific synthesis of literature pertaining to the longitudinal associations of one or more social determinants of health and incident dementia. The social determinants must have been measured at a time when the subject was dementia‐free, dementia status must then also be ascertained at a later time point (with the exception of ethnicity/race, for which we included cross‐sectional analyses as ethnicity/race is obtained by birth). We required that dementia was ascertained either by clinical assessment (direct, self‐reported clinical diagnosis, or via healthcare record linkage) or using a validated algorithm for establishing dementia status based on cognitive and/or functional tests in cohort studies. Two reviewers (Se.W. and A.G.) independently screened articles for inclusion and resolved conflicts through discussion.

Data from all included reviews, including the Lancet Commission reports,^11^, ^22^, ^23^ were extracted using a template that captured the information presented in Table S3.

#### 2.2.2 Update search for primary literature review

We then searched for primary literature. For those SDOD that were covered by systematic reviews identified in stage 1, we searched for primary literature published since the search date of the published systematic review. For other SDOD that were not covered by a systematic review, databases were searched from inception (see Table S4). We performed these searches (described fully in Table S2) in Medline via Ovid, Web of Science, and PsycINFO on the 11th of May 2024. Studies were screened by two reviewers independently (Se.W. reviewed all articles, and M.K., Sh.W., E.H., and U.S. reviewed 25% each), followed by conflict resolution. Eligibility criteria for this stage were the same as stage 1 (except that the study needed to be an original article and not a systematic review). Extraction was completed by one reviewer (A.G.) and checked by a second reviewer (M.K., Sh.W., E.H., U.S., 25% each) for accuracy, using a template which captured the data as presented in Table S5.

### 2.3 Synthesis

The narrative synthesis of extracted data is structured by six social determinants of health domains, as identified from sources detailed in Table S1. These domains are: (1) food environment, (2) physical environment, geography and pollution, (3) housing and sanitation, (4) income, employment, working conditions, socioeconomic status, and poverty, (5) social inclusion and ethnicity, and 6) education. Specific examples for each of the six SDOD domains were identified. In cases where a study included data relevant to multiple domains (e.g., occupational electromagnetic field exposure), we included this study in both relevant domains (e.g., domains 2 and 4). The effects of SDOD mediators that might be situated on the causal pathway from another SDOD to incident dementia (e.g., ethnicity → socioeconomic position → dementia risk) and SDOD that modified the effect of another SDOD (e.g., occupational complexity by ethnic groups), were out of the scope of this scoping review for context‐specific intercorrelation and comparability reasons.

For each specific SDOD, we describe the level of evidence after reviewing the Lancet Commission reports, systematic reviews, and primary literature.

## 3 RESULTS

### 3.1 Search results

**FIGURE 1:**
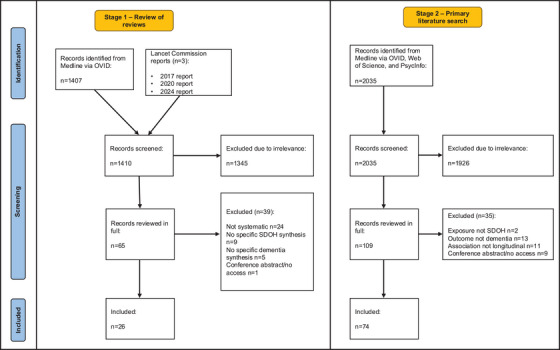
The Preferred Reporting Items for Systematic reviews and Meta‐Analyses (PRISMA) flowchart.

**FIGURE 2:**
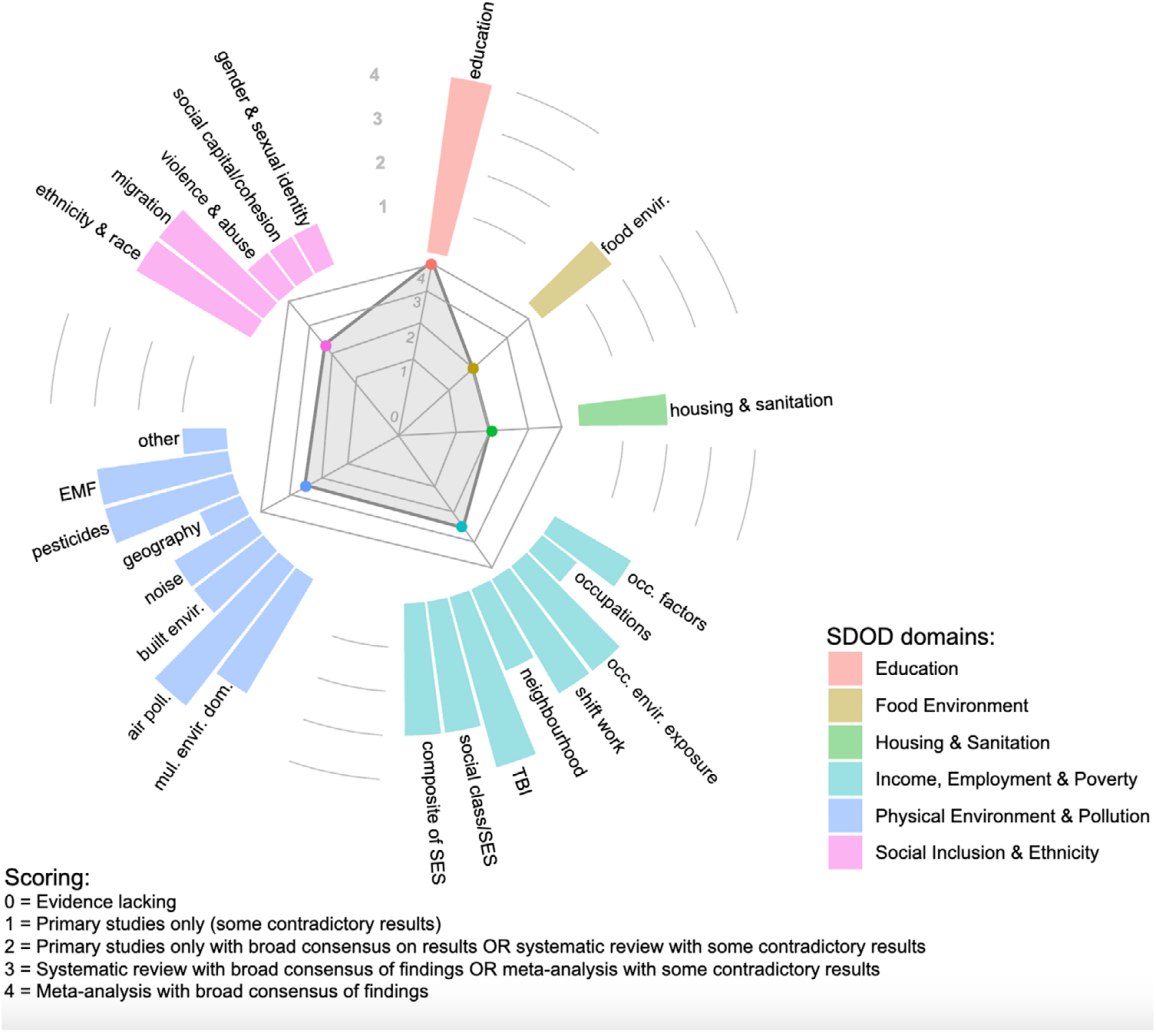
Modified radar plot showing the availability of current evidence on social determinants of dementia, by domain.

We considered 3445 articles for inclusion, of which 100 (*n* = 26 reviews; *n* = 74 primary studies) were included (Figure 1). Extracted data are reported in full in Supplementary Tables S3 and S5, and a narrative summary of available evidence is reported for each SDOD domain below. An overall summary of the findings is depicted in Figure 2.

#### 3.1.1 Food environment

We identified one systematic review which aimed to summarize evidence on the association of food insecurity with multiple outcomes of brain health, including dementia (Table S3).^24^ Although some evidence linking food insecurity to poorer cognition was identified, no studies measuring incident dementia as an outcome were identified by the authors. However, our primary literature search identified two primary studies (Table S5). A population‐based study from the United States found a positive association between experiencing food insecurity and increased dementia risk (odds ratio [OR] = 1.38; 95% confidence interval [CI] = 1.15;1.67).^25^ In contrast, a Japanese study reported an association between subjective availability of food stores and increased dementia risk (hazard ratio [HR] = 1.30[1.10;1.52] lowest vs. highest quartile), while the associations between objective availability of food stores and dementia risk was statistically non‐significant.^26^

#### 3.1.2 Physical environment, geography, and pollution

We identified 10 reviews^11^, ^23^, ^27^, ^28^, ^29^, ^30^, ^31^, ^32^, ^33^, ^34^ (Table S3) and 15 additional primary studies^35^, ^36^, ^37^, ^38^, ^39^, ^40^, ^41^, ^42^, ^43^, ^44^, ^45^, ^46^, ^47^, ^48^, ^49^ related to environmental SDOD (Table S5).

The Lancet Commission highlights a wealth of evidence, including two systematic reviews with meta‐analyses, linking ambient air pollution to dementia risk.^11^, ^23^ Specific cohort studies highlighted by the commission included those notable for their particularly low risk of bias,^50^ and those that reported a significant mediating role of air pollution in explaining some of the racial differences in dementia risk within HICs.^51^, ^52^ The commission reports focused primarily on ambient air pollution, typically measured as particulate matter (PM)2.5 and PM10. We identified further primary studies, which found associations between increased dementia risk and exposure to indoor pollution from solid cooking fuel,^41^ and ambient exposures such as Agent Orange,^42^ respirable aluminum,^48^ and radon.^47^

Two systematic reviews reported on a large range of environmental social determinants of health.^27^, ^28^ The more recent and larger systematic review by Zhao et al.^27^ assessed 132 studies including 44 different natural, physical, and social environmental determinants of health. Associations of several SDOD were meta‐analysed and positive associations were reported for road proximity (*n* = 5), neighborhood deprivation (*n* = 9) (see also 3.2.4), and exposure to aluminium (*n* = 14), solvents (*n* = 12), electromagnetic fields (*n* = 27) (see also 3.2.4), and pesticides (*n* = 14) (see also 3.2.4).^27^ No difference in dementia risk was reported for residential green space (*n* = 7), rurality (*n* = 28), living close to a power line (*n* = 4), noise (*n* = 4), water silicon levels (*n* = 5), and vibrations at work (*n* = 2).^27^ There were too few studies available, and/or measures were too heterogeneous between studies, for other social determinants of health to allow for meta‐analytic comparison. Examples of these included exposure to fluoride^53^ and lithium^54^ in drinking water, and exposure to atomic bombings.^55^

We identified other systematic reviews that presented more focused summaries of evidence. These reviews largely corroborated the findings of the broader Zhao et al. review,^27^ for example, positive associations with dementia risk were reported for road proximity,^29^ electromagnetic fields,^33^, ^34^ pesticides,^32^ and solvents,^32^ whereas findings were mixed for exposure to urban green space.^29^ Mixed findings were also reported for land use (accessibility, walkability, land use mix), and noise exposure.^29^

Primary studies^35^, ^36^, ^37^, ^38^, ^39^, ^40^, ^41^, ^42^, ^43^, ^44^, ^45^, ^46^, ^47^, ^48^, ^49^ were identified that investigated novel potential determinants of health such as exposure to “blue space” (i.e., an outdoor environment that features water; Table S5). In a US‐based cohort study, no significant association was reported between blue space cover and the risk of dementia among fee‐for‐service Medicare beneficiaries aged 65 years or older.^40^ Analysis of a multigenerational population‐based US cohort found that childhood exposure to second‐hand smoke elevates the risk of dementia.^56^ Additionally, ecological studies reported positive associations with dementia risk for several geographical SDOD, including birth in a high stroke mortality state^37^ and area‐level ethnicity.^43^

#### 3.1.3 Housing and sanitation

We identified one systematic review, which searched for US‐based studies on the association between homelessness and dementia risk (Table S3).^57^ Based on six studies, the authors suggested a positive association between homelessness and dementia.^57^

We identified an additional recent primary study on the association between homelessness and dementia in the United States^58^ (Table S5). Roncarati et al.^58^ found that US veterans with housing instability had 1.41 (HR = 1.41[1.36;1.47]) times higher dementia risk compared to veterans without housing instability.

#### 3.1.4 Income, employment, working conditions, socioeconomic status, and poverty

We identified 16 systematic reviews that investigated social determinants of health based on income, employment, working conditions, socioeconomic status, and poverty with dementia risk (Supplementary Table S3).^11^, ^22^, ^23^, ^27^, ^33^, ^34^, ^59^, ^60^, ^61^, ^62^, ^63^, ^64^, ^65^, ^66^, ^67^

Three systematic reviews considered the general construct of socioeconomic position and reported associations between lower socioeconomic position and increased dementia risk.^65^, ^66^, ^67^ Socioeconomic position is a complex concept that can include many individual‐level (e.g., education, income, financial security, occupation) as well as neighborhood‐based measures, making it difficult to directly compare studies. In the meta‐analyses by Bodryzlova et al.,^65^ occupational attainment and income were independently associated with increased dementia risk. A review by Wang et al.^66^ reported an increased dementia risk linked to a lower socioeconomic position composite score (which included occupational attainment and income variables), but did not find an association between solely occupational attainment or solely income with dementia risk.

Nine systematic reviews investigated specific occupations as risk factors.^11^, ^22^, ^23^, ^27^, ^33^, ^34^, ^59^, ^60^, ^61^, ^67^ Meta‐analyses reported an increased dementia risk for shift work (*n* = 2 studies), occupational exposure to magnetic field (*n* = 7 studies), and solvents (*n* = 2) (see also section 3.2.2), whereas no association was identified for non‐manual work compared to manual work (*n* = 6), occupational complexity (*n* = 4), occupational pesticide exposure (*n* = 2) (see also section 3.2.2), or night shifts (*n* = 2).^59^ In contrast, earlier reviews (which did not meta‐analyse results) observed a higher dementia risk for those in manual professions or with lower work complexity in four out of five cohort studies in one review^60^ and four out of four studies in the other.^67^ A review by Then et al.^61^ divided work complexity into four types, and noted that “people complexity” (e.g., training others), and “data complexity” (e.g., performing arithmetic operations) showed protective associations, whereas results were mixed for “things complexity” (e.g., using special devices to work) and “work demands” (e.g., perceived control and stress). Lastly, more evidence for shift work (including night shifts) as a risk factor for dementia was found in the systematic reviews by Gao et al.^62^ and Hai et al.,^63^ although the overall evidence base was deemed insufficient for causal association by the Lancet Commission.^11^ Evidence for occupational traumatic brain injury as a modifiable risk factor was also presented by the Lancet Commission.^11^, ^22^, ^23^ The authors described findings from two systematic reviews^68^, ^69^ with meta‐analyses of cohort studies, which reported higher dementia risk for professions with high‐risk of traumatic brain injury (e.g., veterans, professional soccer and rugby players) compared to matched general population samples.^70^, ^71^, ^72^, ^73^, ^74^, ^75^, ^76^

We identified 32 new primary studies which broadens the evidence base for a link between socioeconomic status and dementia risk (Table S5).^4^, ^77^, ^78^, ^79^, ^80^, ^81^, ^82^, ^83^, ^84^, ^85^, ^86^, ^87^, ^88^, ^89^, ^90^, ^91^, ^92^, ^93^, ^94^, ^95^, ^96^, ^97^, ^98^, ^99^, ^100^, ^101^, ^102^, ^103^, ^104^, ^105^, ^106^ The link between socioeconomic status and dementia risk now extends as far back as childhood.^82^, ^92^ A more detailed exploration of occupational factors such as contact with co‐workers^87^ and older age at retirement (≥66 years),^104^ which were reported to be associated with reduced dementia risk in Northern European studies. Occupational conflict^86^ and military employment^101^ were not found to be associated with dementia risk in population‐based cohorts. Further evidence for associations between higher wealth/income and lower dementia risk has been reported in several population‐based cohorts,^4^, ^82^, ^83^, ^88^, ^89^, ^100^ using both individual‐level measures and neighborhood‐based assessments.^4^, ^79^, ^80^, ^81^

#### 3.1.5 Social inclusion and ethnicity

Three systematic reviews on race or ethnicity as SDOD were identified (Table S3).^107^, ^108^, ^109^ The most recent meta‐analysis from Shiekh et al.^107^ included 19 studies primarily from HICs and concluded that Black individuals had an increased dementia risk as compared to White individuals, while findings for Asian and Latino individuals were non‐significant. A large systematic review that was restricted to studies conducted within the United States (*n* = 114) also reported a higher incidence of dementia in African American and Caribbean American populations as compared to Japanese American (men only), Mexican American, and non‐Latino White American populations.^108^ These findings of a higher risk among Black groups, particularly in the United States, were supported by several new primary studies identified by our search (Table S5).^37^, ^43^, ^79^, ^83^, ^85^, ^89^, ^106^, ^110^, ^111^, ^112^, ^113^, ^114^, ^115^, ^116^, ^117^, ^118^, ^119^, ^120^, ^121^, ^122^ A systematic review restricted to the Asia‐Pacific region reported lower dementia prevalence among ethnic Chinese, compared to ethnic Malay and ethnic Indian individuals, across three comparative studies within Singapore.^109^ Finally, a higher dementia prevalence was observed among Indigenous Australian individuals compared to the general Australian population.^109^

One systematic review on migration and dementia risk included seven studies from the United Kingdom, the Netherlands, and Norway (Table S3).^123^ In a pooled analysis, which combined migrants with ethnic minorities without a report of migration history, dementia risk was found to be higher for migrants/ethnic minorities compared to natives (OR = 1.73[1.42;2.11]). No association was observed between the age of migration and future dementia risk^124^ (Table S5).

We identified several new, primary studies that investigated the association between characteristics of the social environment and dementia risk (Table S5). Most reported elevated dementia risk associated with characteristics of the social environment, such as higher neighborhood disorder,^80^, ^125^ increased neighborhood social vulnerability,^114^ social isolation,^80^ and lower social capital.^126^, ^127^ Two studies, however, found no association with dementia risk for neighborhood social cohesion.^80^, ^125^

Other SDOD in this domain were also identified that were reported to have an association with increased dementia risk in primary studies, which included sibling loss,^112^ transgender sexual identity,^128^ being victims of financial abuse in later‐life^129^ or severe spousal physical abuse,^130^ and adverse childhood experiences.^127^, ^131^, ^132^ No association was found for intimate partner violence^133^ (using a less severe threshold than Leung et al.^130^), physical or psychological abuse.^129^ Findings about sexual relationships were mixed with one study reporting an increased risk for those in a same‐sex relationship,^134^ while another study found no association between sexual relationships (same‐sex or opposite‐sex relationships) and dementia risk (Table S5).^135^

#### 3.1.6 Education

The Lancet Commission reports on dementia determined low formal educational attainment to be a causal modifiable risk factor for dementia, with possible direct and indirect effects on cognitive reserve (Table S3).^11^, ^22^, ^23^ This was based on a broad evidence base that included evidence from HIC and LMICs, as well as within‐country comparisons among ethnic minority groups. Education was typically assessed by attainment (e.g., ranging from no education to university degree). The Commission authors further postulated that educational attainment disparities might explain much of the increased dementia risk for women (beyond the effects of longer life expectancy).^11^, ^136^

## 4 DISCUSSION

### 4.1 Main findings

In this scoping review, we systematically summarized the current evidence linking social determinants of health with dementia risk, highlighting a rapidly growing body of research that demonstrates increasing maturity. We built on existing definitions and conceptual work to define SDOD, and synthesising evidence across reviews (*n* = 26) and primary studies (*n* = 74). We identified evidence of associations with dementia risk across six domains of SDOD (food environment; physical environment geography, and pollution; housing and sanitation; income, employment, working conditions, socioeconomic status, and poverty; social inclusion and ethnicity; and education).

The evidence base for some SDOD is large, robust, and consistent, such as low education, air pollution, and the overall effect of low socioeconomic status. For other SDOD, evidence bases are less robust (e.g., food environment), heterogeneous (e.g., environmental exposures) or are simply lacking (e.g., incarceration). Overall, the field requires continued efforts to identify robust evidence across diverse populations, more standardised measures for SDOD, and more collaborative and harmonisation approaches to compare associations across contexts. Other challenges for the field include developing more robust methods to reduce confounding and selection biases, and to ensure that there is a sufficient length of follow‐up to avoid reverse causality.

The lack of studies in the domains of food environment, housing and sanitation, and social inclusion, highlights the need for more primary studies in these areas. For example, no studies on agriculture, availability of fast food, food programs, housing quality, sanitation, racism, discrimination, or incarceration were identified. Research on these potential SDOD often encounters challenges related to insufficient data availability or difficulties with longitudinal follow‐up, such as those arising from housing instability. Nevertheless, advances in data collection and the emergence of novel databases have facilitated progress in this area. For instance, Veteran Affairs records have been used in a novel way to investigate homelessness in the US.^58^ It is established that discrimination is associated with general health and cardiovascular health outcomes.^137^ For example, discrimination has been linked to hypertension,^138^ medication non‐adherence,^139^ and missed healthcare appointments.^140^ These factors may therefore be expected to increase dementia risk through cerebrovascular disease burden.^141^, ^142^ Alternatively, discrimination may act on dementia risk through mental health^143^ and/or poor lifestyle, including substance use.^144^ Furthermore, most research in areas such as built environment and pollution were conducted in HICs which may have limited generalisability to LMICs, where most dementia cases occur and certain exposures (e.g., to solid cooking fuel) are more common.

Factors related to low socioeconomic status were generally found to be associated with increased dementia risk. For example, the following indicators of socioeconomic status were associated with elevated risk of dementia: reduced wealth or income, manual work or occupations classified as “low complexity,” shift work, occupational exposure to magnetic fields and solvents, and neighborhood disadvantage. However, variability in findings across studies may have arisen from selection/attrition biases, different adjustment for confounders across studies, as well as differences in outcome and exposure classification. More focused reviews, which include quality appraisal processes, could explore these possibilities in more detail. An added consideration is that the same socioeconomic indicator may have a different role in different settings. This may also be the case for differences based on ethnicity or race, which may differ from one context to another depending on levels of entrenched structural racism and other power imbalances. Moreover, although many studies have been published on ethnicity and race, data from many ethnic groups and populations in the Global South remain limited. Persistent methodological issues continue to present challenges, including those of age standardisation, sampling bias, treatment of confounding factors, and the disaggregation and self‐determination of ethnic groups. To address these gaps, more in‐depth collaborative studies are needed to unravel cultural differences and secular trends in socioeconomic, ethnic, and racial inequalities in dementia prevalence over time.^143^,^145^

### 4.2 Findings in context

Social determinants of health are not likely to be the proximal or downstream “causes” of dementia but may instead be better defined as the upstream “causes of the causes”^146^ that may interact with one another to increase dementia risk across the life course. Targeting social determinants of health is an ideal opportunity for dementia prevention efforts. However, a challenge for researchers lies in disentangling the interrelationships between SDOD and other risk factors.^14^ in addition, vulnerable populations often share multiple risk factors. For instance, veterans are more likely to experience mental health problems, substance abuse, trauma, and are more likely to be homelessness.^147^, ^148^ The high clustering of these risk factors presents significant challenges for research focused on SDOD.^13^

We observed that among the most recent studies we identified, there was an increasing trend towards examining connected relationships between multiple SDOD. For example, effect modification by age, neighborhood income, and walkability has been assessed on the association between residential green space and the risk of dementia.^35^ The findings show that younger ages (65–74 years), lower‐income, and non‐car dependent neighborhoods may benefit most from residential green space.^35^ Furthermore, Tani et al.^46^ observed that sidewalk coverage in Japan was associated with a decreased dementia risk among participants living in urban areas but not among those living in rural areas. Genetic risk factors, such as the ε4‐allele of *apolipoprotein E*, may also modulate the influence of social determinants on dementia risk, underscoring the need for further research. For example, less racial/ethnic disparity was detected in a study among the carriers of apolipoprotein E ε4‐allele suggesting interaction of genetic factors with social determinants of health.^120^

Recent work to develop “polysocial scores” (akin to polygenic risk scores but using multiple social determinants of health variables) could offer a novel opportunity to investigate multiple interconnected social determinants of health, their clustering, and their relationships with dementia risk.^149^, ^150^ Strengthening the causal epidemiology in this space is important. For example, some included studies measured lifestyle behaviors, like smoking or alcohol, as “confounders”. However, classic public health models would suggest that such behaviours are, at least in part, intermediate factors on the causal pathway between social disadvantage and poor health. Thus, more focused studies into specific SDOD should focus on investigating these direct and indirect pathways to inform robust study design with appropriate confounder adjustment.

### 4.3 Strengths and limitations

We adopted a pragmatic approach to cover the breadth of literature on the association between social determinants of health and incident dementia. We systematically designed our search strategies in several stages to ensure good representation of the literature on this topic. When searching for reviews, we restricted our search to Medline via Ovid to capture high quality key systematic reviews in this field, although we may also have missed some reviews published only in other databases. As is standard practice for a scoping review, we did not perform a risk of bias assessment for individual studies.^16^, ^17^, ^18^ This will be particularly important for more focused systematic reviews that build upon this work. We critically scrutinized the methodological strategies of the studies and systematic reviews we included, as this will be helpful to those using our work to inform updated systematic reviews in the future. Although our search strategy was designed to be broad and globally relevant, it is to be noted that most evidence was captured from HICs. It is possible that some SDOD specific to certain global contexts are yet to be uncovered in the literature.

We only included studies measuring incident dementia as an outcome. Other outcome measures, such as cognitive testing, structural brain abnormalities, and cognitive reserve, are clearly important to understanding these relationships and should be included in future studies. Our scoping review approach also precludes detailed considerations of whether the evidence base for any given SDOD is sufficient to assert causality. We aimed to provide a broad overview of the level of existing evidence for each determinant, with the aim of informing more targeted reviews addressing specific knowledge gaps.

### 4.4 Recommendations for future research

Findings from this scoping review can be translated into several recommendations for future studies.

First, the lack of studies in the domains of food environment,^151^ housing^152^ and sanitation, and social inclusion and ethnicity^153^—which have all been proposed for dementia (Table S1) and linked to other non‐communicable diseases—demands more primary studies in these areas on the relationship with dementia. Specific social determinants within other domains remain understudied, such as transport infrastructure, income distribution, unemployment, discrimination, structural conflict, and racism (Table S1).

Second, future studies will benefit from standardised study designs, exposure classification, analyses, and reporting to allow comparisons and achieve a granular understanding of how social determinants are associated with risk of dementia. For example, the World Health Organization recently published a “Preferred Product Characteristics” for blood‐based biomarkers for Alzheimer's disease,^154^ and a similar schema for SDOD could be highly useful. Classification systems, such as the International Standard Classification of Occupations, may produce clearer evidence on the associations between occupational factors and dementia risk.

Third, more cohort studies performed in LMICs and continents outside Northern America and Europe are needed.

Fourth, better data linkage between social and environmental data with healthcare data is highly recommended.

Fifth, there is a need of cohorts with longer follow‐up periods for understudied social determinants of health (Table S1) such as household solid cooking fuel, residential segregation, occupational complexity, and socioeconomic position.

Finally, future studies employing rigorous methodologies can significantly advance the field. For example, ensuring careful causal epidemiology work to elicit causal pathways and confounding factors. This is particularly important given that many apparent confounders may in fact be on the causal pathway to dementia, or be both confounders and mediators within complex system relationships.^155^ Other types of studies and causal inference designs can add value, including natural experiment studies of health and social policy changes, and instrumental variable analysis.

### 4.5 Conclusion

This scoping review identified a large, and growing evidence base for SDOD. Broad consensus has been reached that the evidence for some determinants, such as low education and air pollution, is sufficient to infer causality. For other determinants, including socioeconomic status, occupation, and ethnic minority groups, there are clear and consistent patterns of evidence available from systematic reviews and meta‐analyses. For yet other determinants, such as housing and food insecurity, the evidence is still emerging. Most evidence originates from HICs, despite an increasing majority of people living with dementia in LMICs. Future research must address this imbalance while remaining attuned to the challenges of disentangling complex relationships among interacting social phenomena, which are often highly context specific. Results of this scoping review provide actionable research recommendations for future studies, which in turn may inform much‐needed health policy actions to increase population dementia risk equitably. Future risk reduction strategies and policies should therefore incorporate a wider range of dementia risk factors informed by our evolving understanding of the social determinants of health.

## CONFLICT OF INTEREST STATEMENT

The authors declare no conflicts of interest. Author disclosures are available in the Supporting Information.

## Supporting information



Supporting Information

Supporting Information

Supporting Information
