# Supplementary material for: Social determinants of dementia: A scoping review
Source: Alzheimers Dement. 2025 Jul 28;21(7):e70524. doi: 10.1002/alz.70524 (PMC12301702; doi:10.1002/alz.70524)
Supplement: Supplementary file 1 — Supporting Information [file ALZ-21-e70524-s003.docx]

**Supplementary Material**

**Social determinants of dementia: a scoping review**

Sebastian Walsh, Matthias Klee, Esther K. Hui, Usman Saeed, Sheena Waters, Isla Kuhn, Joyce Siette, Vasiliki Orgeta, Jean Stafford, Laura J. Smith, Stefano Tamburin, Daria E.A. Jensen, Elisa Mantovani, Avinash Chandra, Sarah-Naomi James, Eugene Y.H. Tang, David J. Llewellyn, Isabelle F. Foote, Scott T. Chiesa, and Anouk F.J. Geraets and the DEMON SDOH International Research Group

**Table S.1 Defining social determinants of health and eliciting examples to inform our search strategy from existing work**

|  | **Source** | | | | | **Common themes** |
| --- | --- | --- | --- | --- | --- | --- |
|  | World Health Organization* | US Dept. of Health, Office of Disease Prevention & Health Promotion† | Dahlgren & Whitehead and UK Govt.‡ | Adkins-Jackson et al., 2023 (microsystem)§ | Raphael et al., 2020 (Canada) ¶ |  |
| **Definition** | The non-medical factors that influence health outcomes. They are the conditions in which people are born, grow, work, live, and age, and the wider set of forces and systems shaping the conditions of daily life | The conditions in the environments where people are born, live, learn, work, play, worship, and age that affect a wide range of health, functioning, and quality-of-life outcomes and risks. | The broad social and economic circumstances that together influence health throughout the life course are known as the ‘social determinants of health’. | The “conditions in the environments where people are born, live, learn, work, play, worship, seek health care, and age.” | "The primary factors that shape the health of Canadians are not medical treatments or lifestyle choices but rather the living conditions they experience. These conditions have come to be known as the social determinants of health.” | Definition for our work: 'conditions, beyond medical, demographic, and individual lifestyle factors, which may influence people's dementia risk through their life' |
| **Examples** | Food insecurity | Food access | Food production | Food access | Food insecurity | Food environment |
|  |  |  | Agriculture | Food environment |  |  |
|  |  | Transport infrastructure |  | Green space | Geography | Physical environment, Geography, and pollution |
|  |  | Physical activity opportunities |  | Transport infrastructure |  |  |
|  |  | Polluted air |  |  |  |  |
|  | Housing  Basic amenities | Safe housing  Polluted water | Sanitation  Housing | Housing | Housing | Housing and sanitation |
|  | Income | Income | Work | Poverty | Income | Income, employment, working conditions, and poverty |
|  | Social protection | Economic stability | Environment | Workplace | Income distribution |  |
|  | Unemployment | Job opportunities | Employment | Occupation | Social safety net |  |
|  | Job insecurity |  |  |  | Unemployment |  |
|  | Working life conditions |  |  |  | Job security |  |
|  | Structural conflict | Violence |  | Policing | Social exclusion | Social inclusion, violence, and discrimination |
|  | Social inclusion | Racism |  | Incarceration | Indigenous |  |
|  | Non-discrimination | Discrimination |  |  | ancestry |  |
|  |  |  |  |  | Gender |  |
|  |  |  |  |  | Immigration Race |  |
|  |  |  |  |  | Globalization |  |
|  | Education | Education access | Education | Education | Education | Education |
|  | Early childhood | Education quality |  |  | Early child |  |
|  | development | Language/literacy skills |  |  | development |  |

Note:

* World Health Organization. Social determinants of health. Accessed 14 March 2025. <https://www.who.int/health-topics/social-determinants-of-health#tab=tab_1>

† US Office of Disease Prevention & Health Promotion. Social Determinants of Health. Accessed 14 March 2025. <https://odphp.health.gov/healthypeople/priority-areas/social-determinants-health#:~:text=Social%20determinants%20of%20health%20(SDOH,of%2Dlife%20outcomes%20and%20risks>

‡ Dahlgren & Whitehead and UK Govt (2017). *Chapter 6: Social determinants of health in Health profile for England.* Accessed 14 March 2025. <https://www.gov.uk/government/publications/health-profile-for-england/chapter-6-social-determinants-of-health#:~:text=The%20broad%20social%20and%20economic%20circumstances%20that%20together%20determine%20the,%3A%20Dahlgren%20and%20Whitehead%20(1991)>

§ Adkins‐Jackson, P. B., George, K. M., Besser, L. M., Hyun, J., Lamar, M., Hill‐Jarrett, T. G., ... & Babulal, G. (2023). The structural and social determinants of Alzheimer's disease related dementias. *Alzheimer's & Dementia*, *19*(7), 3171-3185. <https://doi.org/10.1002/alz.13027>

¶ Raphael, D., Bryant, T., Mikkonen, J., & Alexander, R. (2020). *Social determinants of health: The Canadian facts*. Ontario Tech University Faculty of Health Sciences.

**Table S.2 Full search strategies**

(i) Lancet reports (Advanced copy provisionally reviewed January 2024, formally extracted 3^rd^ September 2024)

Livingston G, Sommerlad A, Orgeta V, et al. The lancet international commission on dementia prevention and care. Lancet. 2017;390(10113):2673-734.

Livingston G, Huntley J, Sommerlad A, et al. Dementia prevention, intervention, and care: 2020 report of the Lancet Commission. The lancet. 2020 Aug 8;396(10248):413-46.

Livingston G, Huntley J, Liu KY, et al. Dementia prevention, intervention, and care: 2024 report of the Lancet standing Commission. The Lancet. 2024 Aug 10;404(10452):572-628.

Review of reviews (22^nd^ February 2024)

**Ovid MEDLINE(R)** and Epub Ahead of Print, In-Process, In-Data-Review & Other Non-Indexed Citations, Daily and Versions <1946 to February 21, 2024>

1 (dementia* or alzheimer* or lewy bod*).ti. or exp *Dementia/ 207212

2 exp *"Social Determinants of Health"/ or ("social determinant" or "commercial determinant" or " social driver*" or "socioeconomic*").ti. 26905

3 exp *Food Supply/ or exp *Food Security/ or exp *Food Insecurity/ or exp *Fast Foods/ or exp *Access to healthy foods/ or exp *food desert/ or ((food* adj (system* or chain* or procurement or health* or unhealth* or afford* or cost or availab* or fast or junk or secur* or insecur* or vouchers or banks)) or fastfood or takeaway or (food adj3 reformulation) or famine).ti. 18989

4 exp *Environment Design/ or exp *Built Environment/ or exp *Environmental Exposure/ or *Parks, Recreational/ or exp *crowding/ or exp *air/ or exp *weather/ or exp *cities/ or exp *climate/ or exp *environmental pollution/ or exp *fresh water/ or exp *noise/ 616440

5 (cycle lane* or bike lane* or cycle scheme* or walking infrastructure or street light* or green space or park* or "low traffic neigh*" or street closure* or urban design).ti. 97690

6 exp *Ill-Housed Persons/ or exp *Air Pollution, Indoor/ or exp *Residence Characteristics/ or exp *Sanitation/ or exp *home environment/ or (homeless* or (home* adj (warm* or damp* or mould* or slum or poor or insecur*)) or sanitation or hous*).ti. 185945

7 exp *Social Welfare/ or exp *Income/ or exp *Poverty/ or exp *Employment, Supported/ or exp *Employment/ or exp *Socioeconomic Factors/ or exp *Unemployment/ or exp *Occupational Diseases/ or exp *work/ or exp *workplace/ or (income or poverty or social security or social welfare or employ* or unemploy* or job security or earning*).ti. 423981

8 exp *Social Inclusion/ or exp *Racism/ or exp *Exposure to Violence/ or exp *Systemic Racism/ or exp *Violence/ or exp *Social Capital/ or exp *social welfare/ or exp *social environment/ or exp *minority groups/ or exp *"ethnic and racial minorities"/ or exp *ethnicity/ or exp *community resources/ or (social inclusion or social exclusion or racism or violen* or conflict or crime).ti. 274560

9 2 or 3 or 4 or 5 or 6 or 7 or 8 1490364

10 (review or systematic review).pt. 3398884

11 1 and 9 and 10 1407

(ii) Primary literature search (11^th^ May 2024)

**Ovid MEDLINE(R)** and Epub Ahead of Print, In-Process, In-Data-Review & Other Non-Indexed Citations, Daily and Versions <1946 to May 10, 2024>

1 (dementia* or alzheimer* or lewy bod*).ti. or exp Dementia/ 239343

2 exp *Environment Design/ or exp *Built Environment/ or exp *Environmental Exposure/ or *Parks, Recreational/ or exp *crowding/ or exp *weather/ or exp *cities/ or exp *climate/ or exp *environmental pollution/ or exp *fresh water/ or exp *noise/ or (cycle lane* or bike lane* or cycle scheme* or walking infrastructure or street light* or green space or greenness or park* or "low traffic neigh*" or street closure* or urban design or (pollution adj1 (noise or water or environmental)) or electromagnetic field* or overcrowd* or (crowd* adj2 hous*) or ((built or urban) adj environment*) or weather or climate).ti. 749572

3 limit 2 to yr="2021 -Current" 91458

4 exp *Food Security/ or exp *Food Insecurity/ or exp *Access to healthy foods/ or (food* adj (availab* or secur* or insecur*)).ti. 6674

5 limit 4 to yr="2021 -Current" 3043

6 exp *Ill-Housed Persons/ or exp *Air Pollution, Indoor/ or exp *Residence Characteristics/ or exp *Sanitation/ or exp *home environment/ or (homeless* or (home* adj (warm* or damp* or mould* or slum or poor or insecur*)) or sanitation or hous*).ti. 187714

7 exp *Employment, Supported/ or exp *Employment/ or exp *Unemployment/ or exp *Occupational Diseases/ or exp *work/ or exp *workplace/ or (employ* or unemploy*).ti. 241243

8 limit 7 to yr="2020 -Current" 29268

9 (Shift work or night shift*).ti. 2768

10 limit 9 to yr="2022 -Current" 459

11 exp *Income/ or exp *Socioeconomic Factors/ or (income or earning*).ti. or exp *"Social Determinants of Health"/ or ("social determinant*" or "commercial determinant*" or " social driver*" or "socioeconomic*").ti. 226683

12 limit 11 to yr="2022 -Current" 20388

13 exp *Social Welfare/ or exp *Poverty/ or (poverty or social security or social welfare or job security).ti. 65638

14 exp *Social Inclusion/ or exp *Racism/ or exp *Exposure to Violence/ or exp *Systemic Racism/ or exp *Violence/ or exp *Social Capital/ or exp *social welfare/ or exp *social environment/ or exp *community resources/ or exp *minority groups/ or exp *sexuality/ or (sexuality or sexual orientation* or gay or heterosexual or homosexual or LGBT* or lesbian or "same sex" or "sexual minority" or transgender or "trans gender" or transsexual or "trans sexual" or social inclusion or social exclusion or racism or violen* or conflict or crime or migrant or migration or non-binary or nonbinary or lgbqt* or bisexual* or bi-sexual*).ti. 354675

15 exp *"ethnic and racial minorities"/ or exp *ethnicity/ or ethnic minority.ti. 56278

16 3 or 5 or 6 or 8 or 10 or 12 or 13 or 14 688105

17 (cohort or longitudinal or case-control or follow-up or follow up or prospective or retrospective or incidence or incident or risk).ti,ab. or exp Cohort Studies/ or exp Case-Control Studies/ 6517874

18 (cross-section* or cross section* or prevalence).ti,ab. or exp cross-sectional studies/ 1424987

19 1 and 16 and 17 1276

20 1 and 15 and (17 or 18) 255

21 19 or 20 1520

**# Web of Science Search Strategy (v0.1)**

# Database: Web of Science Core Collection

# Entitlements:

- WOS.IC: 1993 to 2024

- WOS.CCR: 1985 to 2024

- WOS.SCI: 1900 to 2024

- WOS.AHCI: 1975 to 2024

- WOS.BHCI: 2008 to 2024

- WOS.BSCI: 2008 to 2024

- WOS.ESCI: 2019 to 2024

- WOS.ISTP: 1990 to 2024

- WOS.SSCI: 1956 to 2024

- WOS.ISSHP: 1990 to 2024

# Searches:

1: TI=(dementia* or alzheimer* or “lewy bod*”) Date Run: Tue May 14 2024 16:56:21 GMT+0100 (British Summer Time) Results: 194739

2: TI=(“cycle lane*” or “bike lane*” or “cycle scheme*” or “walking infrastructure” or “street light*” or “green space” or park* or "low traffic neigh*" or “street closure*” or “urban design” or (pollution near/1 (noise or water or environmental)) or “electromagnetic field*” overcrowd* or (crowd* near/2 hous*) or ((built or urban) near/1 environment*) or weather or climate) Timespan: 2021-01-01 to 2025-12-31 Date Run: Tue May 14 2024 16:56:52 GMT+0100 (British Summer Time) Results: 109641

3: TI= (food* adj (availab* or secur* or insecur*)) Timespan: 2021-01-01 to 2025-12-31 Date Run: Tue May 14 2024 16:57:38 GMT+0100 (British Summer Time) Results: 6661

4: TI= (homeless* or (home* near/1 (warm* or damp* or mould* or slum or poor or insecur*)) or sanitation or hous*) Timespan: 2021-01-01 to 2025-12-31 Date Run: Tue May 14 2024 16:57:51 GMT+0100 (British Summer Time) Results: 36891

5: TI=(employ* or unemploy* or work or workplace* or “occupational disease*”) Timespan: 2020-01-01 to 2025-12-31 Date Run: Tue May 14 2024 16:58:20 GMT+0100 (British Summer Time) Results: 137302

6: TI=(Shift work or night shift*) Timespan: 2022-01-01 to 2025-12-31 Date Run: Tue May 14 2024 16:58:33 GMT+0100 (British Summer Time) Results: 759

7: TI= ("social determinant*" or "commercial determinant*" or " social driver*" or "socioeconomic*" or income or earning*) Timespan: 2022-01-01 to 2025-12-31 Date Run: Tue May 14 2024 16:58:42 GMT+0100 (British Summer Time) Results: 23424

8: TI= (poverty or “social security” or “social welfare” or “job security”) Date Run: Tue May 14 2024 16:58:48 GMT+0100 (British Summer Time) Results: 47297

9: TI= (sexuality or “sexual orientation*” or gay or heterosexual or homosexual or LGBT* or lesbian or "same sex" or "sexual minority" or transgender or "trans gender" or transsexual or "trans sexual" or “social inclusion” or “social exclusion” or racism or violen* or conflict or crime or migrant or migration or “non-binary” or nonbinary or lgbqt* or bisexual* or “bi-sexual*” or “community resource*” or “minority group*” or violence or racism) Date Run: Tue May 14 2024 16:58:56 GMT+0100 (British Summer Time) Results: 556463

10: TI=( "ethnic minorit*" or ethnicity) Date Run: Tue May 14 2024 16:59:12 GMT+0100 (British Summer Time) Results: 32469

11: #2 OR #3 OR #4 OR #5 OR #6 OR #7 OR #8 OR #9 Date Run: Tue May 14 2024 16:59:24 GMT+0100 (British Summer Time) Results: 902944

12: TI=(cohort or longitudinal or case-control or follow-up or follow up or prospective or retrospective or incidence or incident or risk) or AB=(cohort or longitudinal or case-control or follow-up or follow up or prospective or retrospective or incidence or incident or risk) Date Run: Tue May 14 2024 16:59:36 GMT+0100 (British Summer Time) Results: 7273069

13: TI=(cross-section* or cross section* or prevalence) or AB=(cross-section* or cross section* or prevalence) Date Run: Tue May 14 2024 16:59:40 GMT+0100 (British Summer Time) Results: 1678448

14: #1 AND #11 AND #12 Date Run: Tue May 14 2024 16:59:49 GMT+0100 (British Summer Time) Results: 455

15: #1 and #10 and (#12 or #13) Date Run: Tue May 14 2024 17:00:11 GMT+0100 (British Summer Time) Results: 66

16: #14 OR #15 Date Run: Tue May 14 2024 17:00:30 GMT+0100 (British Summer Time) Results: 516

**Psycinfo**

| # | Query | Limiters/Expanders | Last Run Via | Results |
| --- | --- | --- | --- | --- |
| S1 | TI(dementia* or alzheimer* or "lewy bod*") | Expanders - Apply equivalent subjects Search modes - Boolean/Phrase | Interface - EBSCOhost Research Databases Search Screen - Basic Search Database - APA PsycInfo | 71,500 |
| S2 | DE "Dementia" OR DE "AIDS Dementia Complex" OR DE "Alzheimer's Disease" OR DE "Dementia with Lewy Bodies" OR DE "Frontotemporal Lobar Degeneration" OR DE "Presenile Dementia" OR DE "Pseudodementia" OR DE "Senile Dementia" OR DE "Vascular Dementia" | Expanders - Apply equivalent subjects Search modes - Boolean/Phrase | Interface - EBSCOhost Research Databases Search Screen - Basic Search Database - APA PsycInfo | 98,578 |
| S3 | S1 OR S2 | Expanders - Apply equivalent subjects Search modes - Boolean/Phrase | Interface - EBSCOhost Research Databases Search Screen - Basic Search Database - APA PsycInfo | 101,794 |
| S4 | TI(("cycle lane*" or "bike lane*" or "cycle scheme*" or "walking infrastructure" or "street light*" or "green space" or park* or "low traffic neigh*" or "street closure*" or "urban design" or (pollution n1 (noise or water or environmental)) or "electromagnetic field*" or overcrowd* or (crowd* n2 hous*) or ((built or urban) n1 environment*) or weather or climate)) | Expanders - Apply equivalent subjects Search modes - Boolean/Phrase | Interface - EBSCOhost Research Databases Search Screen - Basic Search Database - APA PsycInfo | 38,493 |
| S5 | ((((((((MM "Environmental Planning" OR MM "Interior Design" OR MM "Sustainable Development" OR MM "Urban Planning") OR (MM "Built Environment")) OR (MM "Recreation Areas" OR MM "Playgrounds" OR MM "Public Space")) OR (MM "Crowding")) OR (MM "Atmospheric Conditions" OR MM "Climate Change" OR MM "Extreme Weather")) OR (MM "Urban Environments")) OR (MM "Pollution")) OR (MM "Auditory Stimulation" OR MM "Auditory Displays" OR MM "Auditory Feedback" OR MM "Dichotic Stimulation" OR MM "Filtered Noise" OR MM "Loudness" OR MM "Pitch (Frequency)" OR MM "Rhythm" OR MM "Silence" OR MM "Tempo" OR MM "White Noise")) OR (MM "Noise Levels (Work Areas)") | Expanders - Apply equivalent subjects Search modes - Boolean/Phrase | Interface - EBSCOhost Research Databases Search Screen - Basic Search Database - APA PsycInfo | 63,426 |
| S6 | S4 OR S5 | Expanders - Apply equivalent subjects Search modes - Boolean/Phrase | Interface - EBSCOhost Research Databases Search Screen - Basic Search Database - APA PsycInfo | 97,344 |
| S7 | S4 OR S5 | Limiters - Publication Year: 2021-2024 Expanders - Apply equivalent subjects Search modes - Boolean/Phrase | Interface - EBSCOhost Research Databases Search Screen - Basic Search Database - APA PsycInfo | 15,238 |
| S8 | TI(food* n1 (availab* or secur* or insecur*)) | Expanders - Apply equivalent subjects Search modes - Boolean/Phrase | Interface - EBSCOhost Research Databases Search Screen - Basic Search Database - APA PsycInfo | 1,493 |
| S9 | MM "Food Insecurity" | Expanders - Apply equivalent subjects Search modes - Boolean/Phrase | Interface - EBSCOhost Research Databases Search Screen - Basic Search Database - APA PsycInfo | 1,223 |
| S10 | S8 OR S9 | Expanders - Apply equivalent subjects Search modes - Boolean/Phrase | Interface - EBSCOhost Research Databases Search Screen - Basic Search Database - APA PsycInfo | 1,847 |
| S11 | S8 OR S9 | Limiters - Publication Year: 2021-2024 Expanders - Apply equivalent subjects Search modes - Boolean/Phrase | Interface - EBSCOhost Research Databases Search Screen - Basic Search Database - APA PsycInfo | 686 |
| S12 | TI(homeless* or (home* n1 (warm* or damp* or mould* or slum or poor or insecur*)) or sanitation or hous*) | Expanders - Apply equivalent subjects Search modes - Boolean/Phrase | Interface - EBSCOhost Research Databases Search Screen - Basic Search Database - APA PsycInfo | 20,939 |
| S13 | (MM "Homeless" OR MM "Homeless Mentally Ill" OR MM "Homeless Youth") OR (MM "Home Environment" OR MM "Living Arrangements") | Expanders - Apply equivalent subjects Search modes - Boolean/Phrase | Interface - EBSCOhost Research Databases Search Screen - Basic Search Database - APA PsycInfo | 19,141 |
| S14 | S12 OR S13 | Expanders - Apply equivalent subjects Search modes - Boolean/Phrase | Interface - EBSCOhost Research Databases Search Screen - Basic Search Database - APA PsycInfo | 32,238 |
| S15 | TI(employ* or unemploy*) | Expanders - Apply equivalent subjects Search modes - Boolean/Phrase | Interface - EBSCOhost Research Databases Search Screen - Basic Search Database - APA PsycInfo | 39,505 |
| S16 | ((((MM "Employment Status" OR MM "Employability" OR MM "Employment History" OR MM "Job Loss" OR MM "Reemployment" OR MM "Retirement" OR MM "Self-Employment" OR MM "Unemployment") OR (MM "Supported Employment")) OR (MM "Occupational Exposure")) OR (MM "Working Conditions" OR MM "Job Enrichment" OR MM "Noise Levels (Work Areas)" OR MM "Occupational Safety" OR MM "Telecommuting" OR MM "Work Rest Cycles" OR MM "Work Week Length" OR MM "Workday Shifts" OR MM "Working Space")) | Expanders - Apply equivalent subjects Search modes - Boolean/Phrase | Interface - EBSCOhost Research Databases Search Screen - Basic Search Database - APA PsycInfo | 55,613 |
| S17 | S15 OR S16 | Expanders - Apply equivalent subjects Search modes - Boolean/Phrase | Interface - EBSCOhost Research Databases Search Screen - Basic Search Database - APA PsycInfo | 81,356 |
| S18 | S15 OR S16 | Limiters - Publication Year: 2020-2024 Expanders - Apply equivalent subjects Search modes - Boolean/Phrase | Interface - EBSCOhost Research Databases Search Screen - Basic Search Database - APA PsycInfo | 15,127 |
| S19 | TI("Shift work" or "night shift*") | Expanders - Apply equivalent subjects Search modes - Boolean/Phrase | Interface - EBSCOhost Research Databases Search Screen - Basic Search Database - APA PsycInfo | 760 |
| S20 | TI("Shift work" or "night shift*") | Limiters - Publication Year: 2022-2024 Expanders - Apply equivalent subjects Search modes - Boolean/Phrase | Interface - EBSCOhost Research Databases Search Screen - Basic Search Database - APA PsycInfo | 95 |
| S21 | TI("social determinant*" or "commercial determinant*" or " social driver*" or "socioeconomic*") | Expanders - Apply equivalent subjects Search modes - Boolean/Phrase | Interface - EBSCOhost Research Databases Search Screen - Basic Search Database - APA PsycInfo | 10,349 |
| S22 | ((MM "Income (Economic)" OR MM "Salaries" OR MM "Income Level" OR MM "Lower Income Level" OR MM "Middle Income Level" OR MM "Upper Income Level") AND (MM "Socioeconomic Factors" OR MM "Economic Disadvantage" OR MM "Economic Resources" OR MM "Employment Status" OR MM "Income Level" OR MM "Social Class" OR MM "Social Disadvantage" OR MM "Socioeconomic Disparities" OR MM "Socioeconomic Status" OR MM "Socioeconomic Status" OR MM "Family Socioeconomic Status" OR MM "Income Level" OR MM "Social Class")) OR (MM "Social Determinants of Health") | Expanders - Apply equivalent subjects Search modes - Boolean/Phrase | Interface - EBSCOhost Research Databases Search Screen - Basic Search Database - APA PsycInfo | 6,202 |
| S23 | TI(income or earning*) | Expanders - Apply equivalent subjects Search modes - Boolean/Phrase | Interface - EBSCOhost Research Databases Search Screen - Basic Search Database - APA PsycInfo | 13,372 |
| S24 | S21 OR S22 OR S23 | Expanders - Apply equivalent subjects Search modes - Boolean/Phrase | Interface - EBSCOhost Research Databases Search Screen - Basic Search Database - APA PsycInfo | 26,963 |
| S25 | S21 OR S22 OR S23 | Limiters - Publication Year: 2022-2024 Expanders - Apply equivalent subjects Search modes - Boolean/Phrase | Interface - EBSCOhost Research Databases Search Screen - Basic Search Database - APA PsycInfo | 3,420 |
| S26 | TI(poverty or "social security" or "social welfare" or "job security") | Expanders - Apply equivalent subjects Search modes - Boolean/Phrase | Interface - EBSCOhost Research Databases Search Screen - Basic Search Database - APA PsycInfo | 6,350 |
| S27 | (((MM "Welfare Services (Government)") OR (MM "Poverty" OR MM "Food Insecurity" OR MM "Poverty Reduction")) OR (MM "Job Security")) OR (MM "Social Security") | Expanders - Apply equivalent subjects Search modes - Boolean/Phrase | Interface - EBSCOhost Research Databases Search Screen - Basic Search Database - APA PsycInfo | 14,155 |
| S28 | S26 OR S27 | Expanders - Apply equivalent subjects Search modes - Boolean/Phrase | Interface - EBSCOhost Research Databases Search Screen - Basic Search Database - APA PsycInfo | 15,573 |
| S29 | TI(sexuality or sexual orientation* or gay or heterosexual or homosexual or LGBT* or lesbian or "same sex" or "sexual minority" or transgender or "trans gender" or transsexual or "trans sexual" or "social inclusion" or "social exclusion" or racism or violen* or conflict or crime or migrant or migration or "non-binary" or nonbinary or lgbqt* or bisexual* or "bi-sexual*") | Expanders - Apply equivalent subjects Search modes - Boolean/Phrase | Interface - EBSCOhost Research Databases Search Screen - Basic Search Database - APA PsycInfo | 142,271 |
| S30 | ((((((((MM "Social Inclusion") OR (MM "Racism" OR MM "Internalized Racism" OR MM "Systemic Racism")) OR (MM "Exposure to Violence")) OR (MM "Violence" OR MM "Domestic Violence" OR MM "Gender Violence" OR MM "Gun Violence" OR MM "Patient Violence" OR MM "Police Violence" OR MM "Political Violence" OR MM "School Violence" OR MM "Sexual Violence" OR MM "Violent Crime" OR MM "Virtual Violence" OR MM "Workplace Violence")) OR (MM "Social Capital")) OR (MM "Social Environments" OR MM "Academic Environment" OR MM "Animal Environments" OR MM "Communities" OR MM "Home Environment" OR MM "Poverty Areas" OR MM "Rural Environments" OR MM "Suburban Environments" OR MM "Towns" OR MM "Urban Environments" OR MM "Working Conditions")) OR (MM "Community Resources")) OR (MM "Minority Groups" OR MM "Sexual Minority Groups")) OR (MM "Sexuality" OR MM "Sexual Aids") | Expanders - Apply equivalent subjects Search modes - Boolean/Phrase | Interface - EBSCOhost Research Databases Search Screen - Basic Search Database - APA PsycInfo | 194,213 |
| S31 | S29 OR S30 | Expanders - Apply equivalent subjects Search modes - Boolean/Phrase | Interface - EBSCOhost Research Databases Search Screen - Basic Search Database - APA PsycInfo | 284,255 |
| S32 | TI("ethnic minorit*") | Expanders - Apply equivalent subjects Search modes - Boolean/Phrase | Interface - EBSCOhost Research Databases Search Screen - Basic Search Database - APA PsycInfo | 2,444 |
| S33 | (MM "Ethnic Identity") AND (MM "Race and Ethnic Discrimination" OR MM "Racial Disparities" OR MM "Racial and Ethnic Groups" OR MM "African Cultural Groups" OR MM "Asians" OR MM "Blacks" OR MM "European Cultural Groups" OR MM "Indigenous Populations" OR MM "Latinos/Latinas" OR MM "Middle Eastern and North African Cultural Groups" OR MM "Multiracial" OR MM "North American Cultural Groups" OR MM "Oceanian Cultural Groups" OR MM "People of Color" OR MM "Romanies" OR MM "South American Cultural Groups" OR MM "Whites") | Expanders - Apply equivalent subjects Search modes - Boolean/Phrase | Interface - EBSCOhost Research Databases Search Screen - Basic Search Database - APA PsycInfo | 4,279 |
| S34 | S32 OR S33 | Expanders - Apply equivalent subjects Search modes - Boolean/Phrase | Interface - EBSCOhost Research Databases Search Screen - Basic Search Database - APA PsycInfo | 6,684 |
| S35 | (S7 OR S11 OR S14 OR S18 OR S20 OR S25 OR S28 OR S31) | Expanders - Apply equivalent subjects Search modes - Boolean/Phrase | Interface - EBSCOhost Research Databases Search Screen - Basic Search Database - APA PsycInfo | 343,031 |
| S36 | TI(cohort or longitudinal or "case-control" or "follow-up" or "follow up" or prospective or retrospective or incidence or incident or risk) or AB(cohort or longitudinal or "case-control" or "follow-up" or "follow up" or prospective or retrospective or incidence or incident or risk) | Expanders - Apply equivalent subjects Search modes - Boolean/Phrase | Interface - EBSCOhost Research Databases Search Screen - Basic Search Database - APA PsycInfo | 859,826 |
| S37 | DE "Cohort Analysis" | Expanders - Apply equivalent subjects Search modes - Boolean/Phrase | Interface - EBSCOhost Research Databases Search Screen - Basic Search Database - APA PsycInfo | 1,732 |
| S38 | S36 OR S37 | Expanders - Apply equivalent subjects Search modes - Boolean/Phrase | Interface - EBSCOhost Research Databases Search Screen - Basic Search Database - APA PsycInfo | 859,945 |
| S39 | TI("cross-section*" or "cross section*" or prevalence) or AB("cross-section*" or "cross section*" or prevalence) | Expanders - Apply equivalent subjects Search modes - Boolean/Phrase | Interface - EBSCOhost Research Databases Search Screen - Basic Search Database - APA PsycInfo | 246,798 |
|  |  |  |  |  |
| S40 | S3 AND S35 AND S38 | Expanders - Apply equivalent subjects Search modes - Boolean/Phrase | Interface - EBSCOhost Research Databases Search Screen - Basic Search Database - APA PsycInfo | 541 |
| S41 | s3 and s34 and (s38 or s40) | Expanders - Apply equivalent subjects Search modes - Boolean/Phrase | Interface - EBSCOhost Research Databases Search Screen - Basic Search Database - APA PsycInfo | 13 |
| S42 | S40 OR S41 | Expanders - Apply equivalent subjects Search modes - Boolean/Phrase | Interface - EBSCOhost Research Databases Search Screen - Basic Search Database - APA PsycInfo | 549 |

N.B. After consultation with an expert librarian, and running pilot searches to estimate number of hits, we made slight adjustments from the pre-registered protocol to the list of databases searched

**Table S.3 Findings of the included reviews**

|  | | | | | | | | | | | | | | | | | | | |
| --- | --- | --- | --- | --- | --- | --- | --- | --- | --- | --- | --- | --- | --- | --- | --- | --- | --- | --- | --- |
| **Author, Year** | **Search Date** | | | **Comprehensiveness of search strategy and sources of data** | | | **SDOH definition and measure** | | | | | **Study designs** | | **Populations included** | **Findings** | | **Review comments on quality of evidence/ confidence in causal link** | | |
| **Food Environment** | | | | | | | | | | | | | | | | | | | |
| McMichael, 2022 ^1^ | May 2021 | | | Minor concerns: no grey literature search | | | Food insecurity: “Inadequate access to sufficient, safe and nutritious food to meet dietary needs and food preferences for an active and healthy life”  Measured via Food Security Survey Module, experienced hunger in first 15 years of life | | | | | n=0. Review included cross-sectional, cohort, case-control, intervention, and review studies but identified no articles measuring dementia as an outcome | | Review searched for data from general population, anywhere in the world, but found no studies measuring dementia as an outcome | No studies identified. Some evidence of negative associations between food insecurity and poorer cognition, but small evidence base | | n/a | | |
| **Physical Environment and Pollution** | | | | | | | | | | | | | | | | | | | |
| **Multiple environmental domains** | | | | | | | | | | | | | | | | | | | |
| Zhao, 2021 ^2^ | Jan 2021 | | | Minor concerns: not clear what fields were searched for each search term; list of SDOH terms reasonably broad but less comprehensive than previous reviews.  Major concerns: outcome terms limited to ‘dementia’ (rather than e.g. AD) and not clear which fields searched | | | Natural, physical, and social environmental factors: included residential greenness, temperature, road proximity, metals and pesticides, rurality etc. | | | | | n=132. Cohort, cross-sectional, and case-control studies | | Mixed. Mainly population-based studies of older adults, from mix of HIC and LMICs | Mixed findings.  Random effects meta-analyses for dementia risk:  Moderate-quality evidence  **Increased Risk**  Exposure to aluminium OR 1.31 (1.05, 1.65)  Road proximity OR 1.07 (1.03, 1.11)  Exposure to solvents OR 1.22 (1.02, 1.45)  **Protective**  Residential greenness OR 0.98 (0.95, 1.00)  **Null**  Noise exposure OR 1.02 (0.98, 1.08)  Low- and very low-quality evidence  Increased risk for exposure to electromagnetic fields, exposure to pesticides, and living in deprived areas. Null associations for exposure to silicon, vibrations at work, exposure to a power line, and living in a rural area. | | Residential greenness might be favourable, and aluminium, solvents, and road proximity might be risks. More well-designed studies with longer follow-up duration urgently warranted  Exposure levels were mostly categorical variables with a possibility of misclassification. Heterogeneity may have occurred because of large methodology variations. The ethnic and area bias in the analyses of residential greenness and rural living were found, which may be due to the different economic levels and social context and need further exploration. Longitudinal studies could further clarify the causal relationships | | |
| Killin, 2016 ^3^ | Jan 2016 | | | Minor concerns: grey literature search only involved hand-picked literature already known to authors; list of environmental terms provided but not which derivatives were searched for or the fields searched in.  Major concerns: full search strategy not reported. | | | Environmental factors: not defined, but excluded physiological measures without direct link to environmental exposure (e.g. autopsy studies measuring trace elements in brain or serum)  Measures included: toxic heavy metals, other metals and trace elements, occupational-related exposures | | | | | n=52. Reviews, cohort studies, cross-sectional studies, and case-control studies. | | Majority of studies case-control studies with cases ascertained from clinical records but limited reported data on controls. Some cohorts using general adult population. Country of origin not always reported but where it is mostly HICs | Mixed findings  Strong evidence for an increased risk associated with Vitamin D deficiency. Strong evidence of mixed effects for silicon, pesticides, and solvent exposure  Moderate evidence of increased risk associated with occupational exposure to metals. Moderate evidence of null effect associated with diesel motor exhausts. Moderate evidence of mixed effects associated with arsenic, aluminium, selenium, and electromagnetic fields  Weak evidence for differing effects of tens of other environmental exposures | | Little evidence that toxic heavy metals, or indeed most metals, influence dementia risk, apart from aluminium – where larger, better-quality studies suggested an association. Other than silicon, there was little evidence for other trace elements affecting dementia risk, though selenium remains an interesting element. Of the occupational exposures, there was little strong evidence, but the evidence suggests that exposure to some pesticides and, possibly, metals may affect dementia risk. Strong evidence for vitamin D deficiency being associated with raised dementia risk. Moderate evidence for electromagnetic fields, though this complicated exposure requires some unpicking. No studies measured life course exposure well. | | |
| **Air Pollution** | | | | | | | | | | | | | | | | | | | |
| Livingston, 2020 ^4;^; 2024 ^5^ | Unclear. Published August 2024 | | | Unclear: details not reported | | | Air Pollution: Mostly ambient air pollution captured via local monitoring and linked to individuals through home address or modelling of travel patterns. Few studies measured indoor pollution from residential heating source | | | | | n=9 systematic reviews and meta-analyses since 2019 report; specific cohort studies also summarised because they are at low bias risk, describe risk in specific settings, or exposure from specific sources. Newer studies report interactions between air pollution and other factors (e.g. CVD), or use natural experiment designs | | Evidence reported from multiple global contexts | Air pollution, measured by levels of PM2.5 and PM10, judged to be a causal modifiable risk factor by Lancet Commission on dementia. Little evidence as to which particulate matter constituents (e.g. black carbon, nitrates) are important | | It would be valuable to standardise study design, reporting, and analyses to  allow comparisons and achieve a granular understanding.  Given the close link between SES, household conditions, and exposure to air pollution, minimising residual confounding in these studies is difficult | | |
| **Built Environment** | | | | | | | | | | | | | | | | | | | |
| Chen, 2022 ^6^ | September 2020 | | | Minor concerns: no grey literature search | | | Neighbourhood built environment: “human-made physical surroundings”  Measures included: urbanity/rurality, land use, neighbourhood physical disorder, transportation infrastructure, urban design, and urban nature | | | | | n=10. Cohort studies, cross-sectional studies | | General population from mostly urban settings in HICs, aged ≥45 | Mixed findings  3/5 analyses of land use (e.g. neighbourhood resources, accessibility, walkability) reported protective associations, 1/5 mixed, and 1/5 null  3/7 analyses of green space reported protective associations, 2/7 null, 1/7 mixed, and 1/7 a risk association  1/2 measuring residential distance to road reported protective association, 1/2 reported mixed findings | | [Typically grouped dementia with other cognitive findings in the summary, reporting mostly protective associations overall]  Several domains of NBE were shown to impact the cognitive health of older adults, including urbanity/rurality, land use, neighbourhood physical disorder, transportation infrastructure, urban design, and urban nature  [Quality appraisal mainly noted concerns around validity of built environment measures, such as need for more standardisation, life course assessments and changes to build environment, multi-domain measures; and also the need for more mediation and moderation analyses by individual characteristics like physical activity level and income] | | |
| **Noise** | | | | | | | | | | | | | | | | | | | |
| Huang, 2021 ^7^ | October 2020 | | | Minor concerns: no grey literature search, exact search terms not fully reported | | | Chronic noise exposure, excluding age-related hearing loss  Measures included: traffic noise, aircraft noise, general urban noise | | | | | n=6. Cohort studies, cross-sectional studies | | Adults from general population, all data from HICs | Mixed findings  1/4 studies of traffic noise exposure reported an association with reduced dementia risk, whilst 3/4 reported null effects (inclusive of studies above)  2/2 studies of road proximity of residence reported protective associations with living further from traffic  1/1 study suggested high occupational noise exposure was associated with reduced dementia risk, but with limited reporting | | The current studies failed to provide the required evidence to link noise exposure to AD or cognitive decline  [Limitations of evidence base included most studies including noise as a secondary exposure to air pollution, using proximity to road as proxy for actual measurement of traffic noise, no studies focused on high-risk populations such as loud workplaces, imprecise measurement of noise exposure] | | |
| Clark, 2020 ^8^ | March 2019 | | | Major concerns: searched only PubMed thoroughly and Science Direct using a subset of terms, along with including grey literature already known to authors. Not completely clear, but appears only searched in the keyword field | | | Environmental noise  Measures included: road traffic noise, daily general urban city noise; non-dementia outcome studies also included noise at schools, and workplaces such as airports | | | | | n=2. Cohort studies | | Mid- to late-life adults from general population from HICs | Null findings  2/2 cohort studies reported non-significant associations between exposure to noise levels and dementia risk, after adjustment for relevant confounders | | Low quality evidence of no effect  Mainly due to small evidence base rather than quality of cohort studies, which scored as low risk of bias for all domains except one of the two studies which was considered at unclear selection bias due to lack of reporting of response rates | | |
| **Pesticides** | | | | | | | | | | | | | | | | | | | |
| Yan, 2016 ^9^ | April 2016 | | | Minor concerns: no grey literature search, exact search terms not fully reported | | | Pesticides: “known neurotoxins. Most pesticides share several features, such as the ability to induce oxidative stress, mitochondrial dysfunction, α-synuclein fibrillization and neuronal loss” | | | | | n=7. Cohort, case-control with clinical or population controls | | Older adults, all data from HICs | Increased risk  Fixed effects meta-analysis of adjusted effective sizes suggests increased dementia risk OR 1.37 (1.09, 1.71). No significant evidence of publication bias. Significant effect if restricted to cohort studies, population controls, higher study quality, and age-adjustment. Robust to several sensitivity analyses | | Significant positive association between pesticide exposure and incidence of AD. These findings provide powerful evidence supporting the hypothesis that pesticide exposure is related to an increased risk of AD  Further prospective cohort studies and high-quality case-control studies with improved methods for estimating cumulative pesticide exposure and documenting cases of AD are required to validate the existence of a causal relationship. Further studies required to examine associations between specific compounds or functional groups of pesticide exposure | | |
| **Electromagnetic Fields** | |  | |  | | |  | | | | |  | |  |  | |  | | |
| Jalilian, 2018 ^10^ | November 2017 | | | No concerns | | | Occupational exposure to ELF-MF (frequency range of 3 Hz to 300 Hz) | | | | | n=22. Cohort studies, cross-sectional studies | | Limited reporting, but adults in occupations with exposure to magnetic fields (e.g. power lines). Most studies from HICs. Unclear how controls were selected | Increased risk  Random-effects meta-analysis, weighted by study risk of bias score, reported an increased risk of exposure to ELF-MF for AD: RR 1.63 (1.35, 1.96).  Robust exclusion of studies with higher risk of bias scores, restriction to cohort studies, and to use of either contemporaneous medical records or death certificates for outcome ascertainment. 17/22 studies were considered low risk of bias.  Subgroup analysis reported higher RRs for train drivers compared to electrical workers and welders (not significant); and for women (significant). Some evidence of publication bias but estimate robust to adjustment for this | | This systematic review and meta-analysis suggest that occupational exposure to ELF-MF may increase the risk of AD. However, this suggestion should be interpreted with caution given the moderate to high heterogeneity, and indication for publication bias. Bias from confounding, outcome and exposure misclassification is a concern given the high heterogeneity between the studies. Finally, more studies are essential to gain a better understanding | | |
| Garcia, 2008 ^11^ | April 2006 | | | Major concerns: full search strategy and search terms not reported | | | Occupational exposure to ELF-MF (frequencies ranging between 3 and 3000 Hz, and primarily on workers with occupational exposure to power-frequency fields (50–60 Hz)) | | | | | n=12. Cohort studies, case-control studies | | As per Jalilian 2018 | Mostly as per Jalilian 2018, except for higher risk estimate for men (in contrast to Jalilian) | | Main conclusions as per Jalilian 2018.  For future research, results for exposed men and women merit to be evaluated and reported separately. Also, additional efforts should be directed to better characterize ELF-MF exposure situations for female workers and housewives, as to include sufficient numbers of exposed cases from both sexes. It would be useful to investigate common cut-off points of exposure, particularly ≥0.2 and ≥0.5 μT. Some focus on potential non-linear relationships is needed too. Also, information on relevant duration and time windows of exposure is mostly absent. And more evidence is needed on interactions between ELF-EMF exposure and established risk factors for AD, such as age at onset, familial aggregation and isoforms of ApoE lipoprotein gene. | | |
| **Housing and Sanitation** | | | | | | | | | | | | | | | | | | | |
| Babulal, 2022 ^12^ | October 2021 | | | Major concerns: searched for studies from USA only | | | Homelessness: “A complex situation where persons are living in places unfit for human shelter, people who are unstably housed or in transitional housing (including children and families), and people fleeing domestic violence” | | | | | n=6. Not clear but appear to be case-control or cross-sectional studies | | Not fully reported but mostly US veterans, some with co-morbid alcohol use disorder | Increased risk  6/6 studies reported positive associations between homelessness and dementia, though the relationships were not always clearly described and, in some cases, dementia may have been considered the exposure and homelessness the outcome | | Despite slight variances, the consensus surrounding these studies indicates a complex relationship where homelessness can be a risk factor for dementia, a consequence of dementia, and co-vary with other chronic comorbidities. Some evidence suggests that earlier in life, homelessness can lead to neurodegeneration and increase dementia scores in some homeless adults  Limitations of the literature (narratively reported) included: limited longitudinal data and small sample sizes, particularly for non-veteran populations, due to patients being lost to follow-up | | |
| **Income, employment, and poverty** | | | | | | | | | | | | | | | | | | | |
| **Occupational Factors (General)** | | | | | | | | | | | | | | | | | | | |
| Huang, 2020 ^13^ | March 2020 | | | Minor concerns: no grey literature search, not clear which fields were used to search SDOH terms  Major concerns: searched only for ‘dementia’ and ‘cognition’ as outcome terms | | | Occupation: grouped by mental vs. manual work, occupational complexity, specific professions (e.g. veterans), conditions (e.g. job strain, shift work), and exposures (e.g. pesticides, magnetic fields) | | | | | n=30. Cohort studies, case-control studies | | Limited reporting. Mostly population-based studies from HICs | Mixed findings, mostly null  Random effects meta-analysis of dementia risk from cohort studies  **Increased risk**  Shift work RR 1.39 (1.15, 1.69)  Magnetic field RR 1.26 (1.01, 1.57)  Solvents RR1.18 (1.09, 1.28)  **Null**  Mental vs. manual work: RR 0.92 (0.81, 1.04), occupational complexity RR 0.95 (0.91, 1.00),  pesticide exposure RR 1.34 (0.90, 2.00),  night shifts RR 1.86 (0.59, 5.80) | | Higher work complexity with data and people may reduce the risk of dementia while occupational magnetic field exposures elevate the risk  Not all studies adjusted sufficiently for confounders. High levels of heterogeneity existed in our meta-analysis and cannot be fully explained by subgroup analysis.  Established cutoff values should be used to define the degree of exposure in future research on occupational exposure factors. More future studies focusing on specific occupations (categorized by ISCO) may throw light on the associations between occupational factors and cognition | | |
| Redbled, 2016 ^14^ | March 2014 | | | Major concerns: Full search strategy not reported only that ‘occupation’ and ‘dementia’ were key search terms. Search string was dementia terms AND occupation terms AND mini mental terms AND decline or impairment terms. Lines 3 and 4 will have led to exclusion of articles relevant to current review. Only 226 unique articles identified for screening | | | Occupation: the main activity, paid or unpaid, that the subjects carried out throughout their working lives and that can be included in any of the categories of ISCO, or equivalent | | | | | n=10. Cohort studies, case-control studies, cross-sectional studies | | Community-based older adults from mostly HICs | Mixed findings, mostly suggestive of an increased dementia risk for manual workers  4/5 cohort studies reported in summary table report risk associations associated with manual, blue collar, or less complex work. Range of confounders adjusted for across studies, including age, sex, education, health factors, but this is heterogeneous and it's not always clear which reported results are from crude or adjusted analyses | | The results obtained in the present systematic review support the idea that there is an association between the main occupation and the risk of cognitive impairment and dementia in people over 55 years of age  Study designs and methodologies were heterogeneous, limiting ability to compare across studies e.g. categorisation of type of work. No quality assessment of included articles. No assessment for publication bias | | |
| Then, 2014 ^15^ | September 2013 | | | No concerns | | | Psychological work conditions. Includes: demand characteristics of work tasks, impediments to task fulfilment, social conditions and the organisational setting | | | | | n=9. Cohort studies, case-control studies | | Mixed. Some general population cohorts, some veterans, some twin studies. All HICs | Mixed findings  Occupation Complexity -  3/4 studies of people complexity of job reported protective associations for higher complexity  3/4 studies of data complexity of job reported protective associations for higher complexity  1/4 studies of things complexity of job reported a protective association    Occupational Conditions -  Mixed results for 5 studies investigating associations between work demands (e.g., stress, perceived control) with some reporting significant associations and others null | | [Conclusions mostly pooled across dementia risk and cognitive function]  There may be an association between psychosocial workplace factors (e.g., high job control, high work complexity with people and data, intellectually demanding work) and reduced dementia risk. In general, interpretation of the review findings should be made with caution, as we cannot exclude the possibilities of a publication bias or selective reporting within the identified studies  [Excluded low quality studies from their analysis. Studies typically adjusted for age and sex and explored effects of education in subgroup analyses; or compared exposure across twins discordant for dementia status] | | |
| **Occupational environmental exposure** | | | | | | | | |  |  |  |  | |  |  | |  | | |
| Jalilian, 2018 ^10^ | November 2017 | | | | No concerns | | | Occupational exposure to ELF-MF (frequency range of 3 Hz to 300 Hz) | | | | n=22. Cohort studies, cross-sectional studies | | Limited reporting, but adults in occupations with exposure to magnetic fields (e.g. power lines). Most studies from HICs. Unclear how controls were selected | Increased risk  Random-effects meta-analysis, weighted by study risk of bias score, reported an increased risk of exposure to ELF-MF for AD: RR 1.63 (1.35, 1.96).  Robust to exclusion of studies with higher risk of bias scores, restriction to cohort studies, and to use of either contemporaneous medical records or death certificates for outcome ascertainment. 17/22 studies were considered low risk of bias.  Subgroup analysis reported higher RRs for train drivers compared to electrical workers and welders (not significant); and for women (significant). Some evidence of publication bias but estimate robust to adjustment for this | | This systematic review and meta-analysis suggest that occupational exposure to ELF-MF may increase the risk of AD. However, this suggestion should be interpreted with caution given the moderate to high heterogeneity, and indication for publication bias. Bias from confounding, outcome and exposure misclassification is a concern given the high heterogeneity between the studies. Finally, more studies are essential to gain a better understanding | | |
| Garcia, 2008 ^11^ | April 2006 | | | | Major concerns: full search strategy and search terms not reported | | | Occupational exposure to ELF-MF (frequencies ranging between 3 and 3000 Hz, and primarily on workers with occupational exposure to power-frequency fields (50–60 Hz)) | | | | n=12. Cohort studies, case-control studies | | As per Jalilian 2018 | Mostly as per Jalilian 2018, except for higher risk estimate for men (in contrast to Jalilian) | | Main conclusions as per Jalilian 2018.  For future research, results for exposed men and women merit to be evaluated and reported separately. Also, additional efforts should be directed to better characterize ELF-MF exposure situations for female workers and housewives, as to include sufficient numbers of exposed cases from both sexes. It would be useful to investigate common cut-off points of exposure, particularly ≥0.2 and ≥0.5 μT. Some focus on potential non-linear relationships is needed too. Also, information on relevant duration and time windows of exposure is mostly absent. And more evidence is needed on interactions between ELF-EMF exposure and established risk factors for AD, such as age at onset, familial aggregation and isoforms of ApoE lipoprotein gene. | | |
| Zhao, 2021 ^2^ | Jan 2021 | | | Minor concerns: not clear what fields were searched for each search term; list of SDOH terms reasonably broad but less comprehensive than previous reviews.  Major concerns: outcome terms limited to ‘dementia’ (rather than e.g. AD) and not clear which fields searched | | Occupational electromagnetic field and aluminium exposure | | | | | | n=24 (electromagnetic fields), n=4 (aluminium). Cohort, cross-sectional, and case-control studies | | Mixed. Mainly population-based studies of older adults, from mix of HIC and LMICs | Increased risk for exposure to occupational electromagnetic fields OR 1.35 (1.17, 1.55) but no effect for occupational aluminium exposure OR 1.08 (0.68, 1.71) | | Exposure to occupational electromagnetic fields might increase dementia risk factors. More well-designed studies with longer follow-up duration urgently warranted  Exposure levels were mostly categorical variables with a possibility of misclassification. Heterogeneity may have occurred because of large methodology variations. Longitudinal studies could further clarify the causal relationships | | |
| **Shift Work** | | | | | | | | | | | | | | | | | | | |
| Livingston, 2024 ^5^ | Unclear. Published August 2024 | | | Unclear: details not reported | | | Occupation-related sleep disturbance due to shift work captured from occupational records and linked to national registry/ healthcare/ mortality data | | | | | n=2. One systematic review, one cohort study | | Shift workers from Europe | A systematic review found heterogeneous evidence of dementia risk due to shiftwork and could not draw conclusions.  A cohort study found that shift work was associated with an increased risk of dementia HR 1.30 (CI 1.08, 1.58), but risk was not higher in people who worked night shifts than in people who worked day shifts. | | | Shift work discussed in the context of sleep disturbance. Overall, evidence not felt convincing that sleep disturbance is a causal, modifiable risk factor for dementia because of potential reverse causality bias and residual confounding | |
| Gao, 2023 ^16^ | April 2022 | | | No concerns | | | Shiftwork. Any work outside the regular day-time schedule, including fixed rotating shifts, evening or night work, and ordinary two/three shifts; and work performed in the period between 24:00 and 05:00, including three shifts, or permanent night shifts | | | | | n=5. Cohort studies, case-control studies. Unexposed groups were daytime workers | | Adult workers from Europe. One study included women only | Increased risk of dementia  Random effect meta-analysis:  Shift work RR 1.13 (CI 1.05, 1.21)  Night shifts RR 1.13 (1.03, 1.24)  Non-linear relationship observed in cubic spline models, with steeper increase in risk <7 years of shift work | | When compared with daytime work, shift work and night shifts are significantly related to greater dementia risk. Long-term shift work was linked to a higher risk of dementia. These findings indicate that it would be advisable to optimize the shift system and shorten the duration of shift work for workers. Robust to subgroup analyses stratifying by sex, study design, education, lifestyle and health factors  [All studies scored well on quality assessment and adjusted for relevant confounders. However, exposures of shift work were self-reported through interview or questionnaire, while whether workers were engaging in permanent or temporary shift work was not reported in most studies. No assessment of publication bias. Evidence base noted to be fairly small - further population-based studies of other ethnic groups and regions are still needed to confirm the association] | | |
| Hai, 2022 ^17^ | November 2021 | | | Minor concerns: limited search terms used for shiftwork, and not clear what search fields used | | | Shiftwork: night work, evening work, irregular work, rotational work, or a combination of these | | | | | n=4. Cohort studies | | All but one study covered by Gao 2023 | Findings largely as per Gao 2023.  One study differentiated continuous night shifts to other schedules including shift work, reporting reduced dementia risk for those on continuous nights compared to those with variable shift patterns | | Largely as per Gao 2023  Found no evidence of publication bias | | |
| Leso, 2021 ^18^ | September 2020 | | | Major concerns: search strategy not fully reported, only identified 61 articles for screening | | | Shiftwork: working at least 3 hours of the daily shift or a certain proportion of the yearly working time in a period of 7 hours defined by national law and including the time from midnight to 05:00 | | | | | n=6. Cohort studies, case-control studies | | Adult workers from Europe. One study used Swedish twin registry, another nurses | Generally, increased risk  Large overlap of included studies with the Gao and Hai reviews, and narratively described findings similar, although due to a lack of meta-analysis, some individual studies report non-significant trends | | The limited number of available studies, the several and different work schedules analysed, together with the possible co-exposure to other occupational risk factors prevent [us from drawing] conclusions on the shift work-dementia relationship. Further research should confirm such association and the causal relation with early cognitive alterations | | |
| **Traumatic Brain Injury (TBI)** | | | | | | | | | | | | | | | | | | | |
| Livingston, 2017 ^19^; 2020 ^4^; 2024 ^5^ | Unclear. Published August 2024 | | | Unclear: details not reported | | | Occupation-related TBI, specifically working in military or professional sporting occupations. Some studies including only those with documented TBI occurrence, whilst for others the exposure of interest was simply a profession with high TBI risk | | | | | n=9. Systematic reviews and meta-analyses, cohort studies  Reviews included studies where TBI was a social determinant (occupation) and other causes of TBI. Cohort studies with occupational groups compared to matched general population samples | | Military personnel (US studies), professional sportsmen (Europe and US) | Consistent evidence for an increased dementia risk for individuals with traumatic brain injury has been reported | | | | Traumatic brain injury (whether occupationally related or not judged to be a causal modifiable risk factor for dementia |
| **Social Class/Socioeconomic Status** | | | | | | | | | | | | | | | | | | | |
| Bodryzlova, 2023 ^20^ | October 2021 | | | Minor concerns: search lines for AND 'ageing' AND 'prospective' terms may have led to missing relevant articles; sought to retrieve government reports but did not report a search of grey literature | | | Social class: social stratification that includes notions of privilege and lifestyle  Measures included: education, occupation, income, neighbourhood, wealth, living alone | | | | | n=15. Cohort studies | | Population-based studies of people aged ≥60 without dementia at baseline, mostly from HICs | Generally, increased risk  Meta-regression estimates of a 1-unit reduction in social class on RR of dementia, adjusted for age, sex, and year of recruitment:  Education 2.52 (1.81, 3.51)  Occupation 2.37 (CI 1.58, 3.56)  Income 1.13 (CI 0.76, 1.70)  Random effects meta-analysis pooling across indicators suggested a crude RR for dementia of 1.48 (1.30, 1.69) for belonging to a lower social class | | [Authors did not offer a view on confidence in causality]  Studies were rated as excellent (n=2), good (n=10), and acceptable (n=4) with studies generally scoring well for representativeness and selection biases, but scored more poorly for exposure ascertainment due to self-reporting of social class indicators  [Minimal publication bias identified, not thought to have significantly effected results. Noted that key data on confounders like CVD often lacking. No separate analysis for higher-quality studies, no requirement for minimum follow-up period] | | |
| Wang, 2023 ^21^ | January 2022 | | | Minor concerns: no grey literature search | | | SES is a complex concept reflecting a person’s overall status in society, which encompasses income, education, and occupation  Measures included composite measures, parental occupational level, educational attainment, income, own occupational level | | | | | n=25. Cohort studies, case-control study | | Population-based studies of people aged ≥50 without dementia at baseline followed up for ≥1 year, mostly from HICs | Increased risk  Random effects meta-analysis for low compared to high SES reported an estimated RR for dementia of 1.40 (CI 1.12,1.74)  Sub-group meta-analyses reported mixed results, with composite measures and low education remaining significant (income and occupation null), as well comparing low to high risk of bias studies. Meta-regression results stratified by region, study years, response rate, age, follow-up time, baseline cognition, adjusted factors, SES measures used, and quality appraisal score were all non-significant. But these analyses may have been underpowered | | [Conclude this provides observational evidence of an association but were unable to explain the high heterogeneity convincingly as most subgroup analyses reported null effects. Education effects most robust, and education and income highest associative risk. Calls for more study designs to be used, such as Mendelian Randomisation, and assessment of mediation through biological mechanisms. No assessment for publication bias] | | |
| Fratiglioni, 2007 ^22^ | December 2006 | | | Major concerns: full search strategy not reported, limited databases searched, limited search terms | | | SES – no definition. Measures included: education, father’s occupation, own occupation, income, work complexity | | | | | n=33. Cohort studies, case-control studies, cross-sectional studies | | Limited information reported but appear to be population-based cohorts aged ≥60 | Generally, increased risk for lower SES  22/23 studies reported an association between low education level and increased dementia risk  3/6 studies reported an association between low SES and increased dementia risk (1/2 childhood SES; 2/4 adulthood SES).  4/4 studies reported an association between less occupational complexity and increased dementia risk | | [Conclude that the evidence for education is convincing, for SES is mixed and unconvincing, and for occupational complexity is promising but exposure ascertainment heterogeneous and small evidence base. Included only studies which they considered to be internally valid and laid out clear criteria for assessing this, including assessment of exposure, outcome, and inclusion of relevant confounders. However, the results of this process are not reported for each article. No assessment of publication bias] | | |
| Zhao, 2021 ^2^ | Jan 2021 | | Minor concerns: not clear what fields were searched for each search term; list of SDOH terms reasonably broad but less comprehensive than previous reviews.  Major concerns: outcome terms limited to ‘dementia’ (rather than e.g. AD) and not clear which fields searched | | | Neighborhood socioeconomic status | | | | | | | n=9. Cohort, cross-sectional, and case-control studies | Mixed. Mainly population-based studies of older adults, from mix of HIC and LMICs | Low quality evidence for an increased dementia risk for low neighborhood socioeconomic status OR 1.28 (1.16, 1.39). | | Nneighborhood socioeconomic status might increase dementia risk. More well-designed studies with longer follow-up duration urgently warranted  Exposure levels were mostly categorical variables with a possibility of misclassification. Heterogeneity may have occurred because of large methodology variations. Longitudinal studies could further clarify the causal relationships | | |
| **Social Inclusion & Discrimination** | | | | | | | | | | | | | | | | | | | |
| **Ethnicity & Race** | | | | | | | | | | | | | | | | | | | |
| Shiekh, 2021 ^23^ | September 2019 | | | No concerns | | | Ethnicity. White, Non- Latino White, and European American grouped as White; Black, African American, and African-Caribbean as Black; Asian and Asian-American as Asian; and Latino, Hispanic, Caribbean Hispanic, and Cuban Americans as Latino. Other ethnic groups included in this review were American Indian or Alaskan Native, Pacific Islander, Chinese, Malay, Indian, Han, Kazakh, Uyghur, Japanese, Filipino, South Asian, Other/Unknown Asian, and Mixed | | | | | n=19. Cohort studies, cross-sectional studies | | Adults, mainly from HICs | Mixed findings. Increased risk for Black groups  Random effects meta-analysis for dementia risk:  Black (ref White) people had an increased dementia risk in random effects meta-analysis RR 1.33 (1.07, 1.65); comparable RR to 3 studies which couldn’t be meta-analysed due to methods; same direction as cross-sectional analyses; and consistent when restricted to AD as the outcome (these studies higher risk of bias).  From meta-analysis of two studies, and two further non-meta-analysed studies, there was a generally non-significant trend towards a lower RR for dementia for Asian groups (ref White). There were mixed results for Latino groups, with some reporting increased risk (ref White) but most associations null. Risk of bias mixed in these studies. Malay had higher risk (ref: Chinese) RR 2.28 (1.45,3.58) low- to moderate-risk of bias | | Our study suggests important ethnic differences in dementia incidence and prevalence  Only about half of the included studies adjusted for CVD, or other factors e.g. SES, which could be confounders [could also be intermediates]. Studies included are based on electronic health records results from which might be affected by factors such as ethnic differences in healthcare service utilization. These could also be restricted in their measurement of the ethnic groupings and covariates assessed potentially leading to residual confounding  Further high-quality research studies from a range of global settings are needed with sufficiently large sample sizes to enable comparisons within, as well as between, ethnic groups including comparisons of more granular ethnic sub-groups. Future studies might also look at secular trends in ethnic inequalities in dementia risk over time. | | |
| Mehta, 2017 ^24^ | February 2014 | | | Minor concerns: no grey lit search  Major concerns: restricted to studies from the USA only | | | Ethnicity. Not defined | | | | | n=114. Prevalence and incidence (descriptive) studies | | US adult population | Mixed findings  Annual incidence averaged across cohorts considered sufficiently comparable (unclear how age standardisation was conducted if at all):  African American, mean 2.6%  Caribbean Hispanic American, mean 3.6%  Japanese American (men only), mean 2%  Mexican America (n=1 study), mean 0.8%  White, non-Latino American, mean 1.6%  High heterogeneity between studies. No data for American Indians, Alaska Natives, and some groups of Asian Americans such as Chinese, Filipino, Vietnamese, and South Asian groups | | Suggests the strong possibility of increased incidence of dementia in African American and Hispanic Caribbean American populations. Furthermore, it clarifies a clear heterogeneity in incidence and prevalence within the overall category of “Latino” older adults. Disaggregation of this category is strongly recommended in future research and reporting  [Key data gaps were the total lack of prevalence or incidence data for some groups. Key methodological issues included unclear age standardisation, sampling biases (e.g. from nursing home populations), disaggregation of ethnic groups, and challenges with self-determination of ethnicity and those with mixed heritage] | | |
| Venketasu-bramanian, 2011 ^25^ | 2010 | | | Major concerns: only searched for studies from the Asia-Pacific region, only searched Medline, only search for ‘dementia’ and the name of each country or phrases ‘multi-ethnic’ or ‘inter-ethnic’ | | | Ethnicity. Not defined | | | | | n=4. Prevalence (descriptive) studies | | Community-based studies from Singapore and Australia | Unclear findings  Dementia prevalence was lowest among ethnic Chinese, compared to ethnic Malays and ethnic Indians, across 3 comparative studies within Singapore. Differences were not explained by gender, age or education; but cardiovascular factors (hypertension, diabetes, cardiovascular diseases, stroke and smoking), depression and leisure time activities contributed modestly to the differences  Higher prevalence amongst Indigenous Australian compared to general population - but unclear which factors may explain this | | Information on possible interethnic differences are emerging, but more in-depth collaborative studies are needed to address important methodological issues, especially dementia incidence studies that pay close attention to cultural factors that can affect dementia recognition. The issue of how to determine race and ethnicity has significant implications on biomedical research, particularly where it is possible that biological factors may explain disparities seen in health status. Population stratification in association studies can be controlled for by estimating genetic ancestry, which may also help detect a biological basis for causation for population-specific differences in disease | | |
| **Migration** | | | | | | | | | | | | | | | | | | | |
| Selten, 2021 ^26^ | December 2018 | | | Minor concerns: no grey literature search. Aimed to report on risk by ethnic minority status and/or migrant status, but search terms skewed heavily towards the latter  Major concerns: searched only for studies on migrants living in Europe | | | Ethnic minority status or migrants. Not defined | | | | | n=7. Cohort studies, cross-sectional study | | Adults over 50 in the UK, Norway, and the Netherlands | Increased risk  In pooled analysis which combined migrants with ethnic minorities without report of migration history, dementia risk (ref “native populations”) was higher: OR 1.73 (1.42, 2.11). This was higher for population-based surveys (OR 3.10) but null for health care record-based studies (OR 0.94). Results were consistent for subgroup analyses involving African groups and Asian groups | | The evidence presented here suggests that migrants from Asia and Africa are at an increased risk of developing dementia and that they are less likely to reach the services  [Population-based studies rated as higher-quality, because healthcare records may miss those who struggle to access care, though even in the population-based studies the cultural validity of screening tests was uncertain. No evidence of publication bias] | | |
| **Education** | | | | | | | | | | | | | | | | | | | |
| Livingston, 2017 ^19^; 2020 ^4^; 2024 ^5^ | Unclear. Published August 2024 | | | Unclear: details not reported | | | Education measured in various ways:  (1) Attainment (e.g. university degree vs. completing high school vs. primary school vs. no formal education)  (2) Years of education  (3) Average reading level at age 14-15 (marker of education system quality) | | | | | n=8. Systematic reviews and meta-analyses, cohort studies, natural experiment study | | Data from HICs, LMICs, and intra-country comparisons between ethnic minority groups | Low formal educational attainment judged to be a casual modifiable risk factor for dementia, with likely direct and indirect effects on cognitive reserve. | Education differences (as well as life expectancy) reported to explain much of the increased dementia risk for women | | | |
| SDOH = Social Determinant of Health. HIC = High Income Countries. LMIC = Low- and Middle- Income Countries. CVD = Cardiovascular disease. PM = Particulate Matter. SES = Socioeconomic Status. AD = Alzheimer’s disease. OR = Odds Ratio. CI = Confidence Interval. NBE = Neighbourhood Built Environment. ISCO = International Standard Classification of Occupations. RR = Risk ratio. ELF-MF = Extremely low frequency magnetic fields. TBI = Traumatic Brain Injury | | | | | | | | | | | | | | | | | | | |

**Table S.4 Adjustments original search terms for review stage 2**

| **Social determinant of health** | **Original search terms** | **Adjustments** |
| --- | --- | --- |
| Food environment | exp *Food Supply/ or exp *Food Security/ or exp *Food Insecurity/ or exp *Fast Foods/ or exp *Access to healthy foods/ or exp *food desert/ or ((food* adj (system* or chain* or procurement or health* or unhealth* or afford* or cost or availab* or fast or junk or secur* or insecur* or vouchers or banks)) or fastfood or takeaway or (food adj3 reformulation) or famine).ti. | 1. Update 'food insecurity' terms from 2021 only 2. Retain all other search terms from inception |
| Physical environment, geography, and pollution | exp *Environment Design/ or exp *Built Environment/ or exp *Environmental Exposure/ or *Parks, Recreational/ or exp *crowding/ or exp *air/ or exp *weather/ or exp *cities/ or exp *climate/ or exp *environmental pollution/ or exp *fresh water/ or exp *noise/ OR (cycle lane* or bike lane* or cycle scheme* or walking infrastructure or street light* or green space or park* or "low traffic neigh*" or street closure* or urban design).ti. | 1. Contact authors of 2021 (broad) review to elicit complete search strategy - update from 2021 where this was comprehensive 2. Neighbourhood built environment from 2020 (unless covered above) 3. Noise from 2020 (unless covered above) |
| Housing and Sanitation | exp *Ill-Housed Persons/ or exp *Air Pollution, Indoor/ or exp *Residence Characteristics/ or exp *Sanitation/ or exp *home environment/ or (homeless* or (home* adj (warm* or damp* or mould* or slum or poor or insecur*)) or sanitation or hous*).ti. | 1. Retain all search terms from inception |
| Income, employment, working conditions, socioeconomic status, and poverty | exp *Social Welfare/ or exp *Income/ or exp *Poverty/ or exp *Employment, Supported/ or exp *Employment/ or exp *Socioeconomic Factors/ or exp *Unemployment/ or exp *Occupational Diseases/ or exp *work/ or exp *workplace/ or (income or poverty or social security or social welfare or employ* or unemploy* or job security or earning*).ti. OR exp *"Social Determinants of Health"/ or ("social determinant" or "commercial determinant" or " social driver*" or "socioeconomic*").ti. | 1. Contact authors of 2020 (broad) occupation review to elicit complete search strategy - update from 2020 where this was comprehensive 2. Update 'SES' terms from 2022 only 3. Add shiftwork terms, and search from 2022  4. Add magnetic fields terms, and search from 2017 (unless covered in environment review from 2021 above, blue section) |
| Social Inclusion | exp *Social Inclusion/ or exp *Racism/ or exp *Exposure to Violence/ or exp *Systemic Racism/ or exp *Violence/ or exp *Social Capital/ or exp *social welfare/ or exp *social environment/ or exp *minority groups/ or exp *"ethnic and racial minorities"/ or exp *ethnicity/ or exp *community resources/ or (social inclusion or social exclusion or racism or violen* or conflict or crime).ti. | 1. Update 'ethnicity' terms from 2019 only 2. Retain all other terms from inception 3. Add 'migration' terms |

**Table S.5 Findings of the primary literature**

| **Author, Year** | **SDOH** | **Study Design** | **Population** | **Outcome Ascertainment** | **Statistical Analysis** | **Findings** |
| --- | --- | --- | --- | --- | --- | --- |
| **Food Environment** | | | | | | |
| Qian, 2023 ^27^ | Food insecurity.  Assessed using the US Department of Agriculture 6-item Household Food Security Module and classified as food secure, low food secure, and very low food secure | Population-based cohort study  n=7,012  Up to 5-year follow-up | US adults aged ≥50 (Health and Retirement Study) | Dementia probability was estimated using a previously validated algorithm that includes various cognitive questions as well as demographic information | Generalized estimation equations were fit taking selective attrition into account through inverse probability of censoring weights. Confounders included: age at food security assessment (in 2013), sex, self-reported race and ethnicity, educational level, birthplace, married status, age at each interview wave, BMI (self-reported, 2012), and SES indicators | Compared with food-secure older adults, experiencing low food security was associated with higher dementia risk: OR 1.38 (95% CI 1.15, 1.67), as was experiencing very low food security: OR 1.37 (1.11, 1.59) |
| Tani, 2019 ^28^ | Healthy food availability.  The number of food stores selling fruits and vegetables within 500m or 1Km of residence assessed by GIS; and subjective (participant-reported) availability | Population-based cohort study  n=49,511  Mean follow up 2.9 years | Adults aged ≥65 years enrolled in the Japan Gerontological Evaluation Study, who were physically and cognitively independent | Dementia incidence was ascertained by linking the participants to the standardized in-home assessment and medical examination conducted under Japan’s public long-term care insurance registry | Cox proportional hazards models were used adjusting for age, sex, education, income, living situation, marital status, employment status, driving status, public transportation, prefecture of residence, BMI, frequency of vegetable/fruit intake, walking time, frequency of going out, hypertension, DM, hearing loss, depressive symptoms, ADLs, cognitive complaints, and population density | Compared to the highest quartile, less healthy food availability was generally associated with an increased dementia risk for other quartiles. Using objective measures, lower availability at 500m and 1km was significantly associated with dementia risk for quartiles 2 and 3 but not significant for lowest quartile in fully adjusted models: 500m: HR 0.98 (0.87, 1.11); 1Km: HR 1.16 (1.00, 1.34). Most other models and quartiles significant, with an HR between 1.2 and 1.6. Subjective availability of food stores was associated with an increased dementia risk: HR 1.30 (1.10, 1.52) for lowest quartile |
| **Physical Environment, Geography and Pollution** | | | | | | |
| **Built Environment** | | | | | | |
| Brown, 2024 ^29^ | Greenness.  Measured at the Census block level, the smallest geographic area for which the Bureau of the Census collects decennial census data, using the Normalized Difference Vegetation Index | Population-based cohort study  n=230,738  Up to 5-year follow-up | USA Medicare beneficiaries aged ≥65 years living in Miami-Dade County, Florida, living in consistently high and low greenness Census blocks between 2011 and 2016 | Incidence of a new AD condition from 2012 through 2016 was assessed with the USA Centers for Medicare and Medicaid Services Chronic Condition Algorithm for AD based on ICD-9 codes | Multi-variable Generalized Estimating Equations models with exchangeable correlation structure were used adjusted for adjusting for age, sex, race/ethnicity, and neighbourhood income | Older adults living in the consistently high greenness tertile, compared to those in the consistently low greenness tertile, had 16% lower odds of AD incidence: OR 0.84 (0.76, 0.94). Age, neighbourhood income and walkability moderated greenness’ relationship to odds of AD incidence, such that younger ages (65-74), lower-income, and non-car dependent neighbourhoods may benefit most from high greenness |
| Godina, 2023 ^30^ | Neighbourhood greenspace.  Percentage of greenspace and forest (tree canopy cover); and tertiles of greenspace diversity (number of greenspace types), for 5km radial buffers around participants’ residences, from the 1992 National Land Cover Dataset | Community-based cohort study  n=2,141  Up to 10-year follow-up | Older adults (mean age = 75.3) from the Cardiovascular Health and Cognition Study (recruited through random sampling of Medicare eligibility lists at multiple sites) | Dementia was evaluated by detailed neurological, and neuropsychological examinations. The possible cases of dementia were clinically adjudicated by a review committee of neurologists and psychiatrists | Cox proportional hazard regression analyses adjusting for age, race, sex, education, income, hypertension, DM, co-morbid index, BMI, smoking status, APOE e4 allele, neighborhood SES, urbanicity, and study site | Mostly null results for associations between greenspace, forest cover, and greenspace diversity and dementia risk |
| Hu, 2023 ^31^ | Residential greenness.  Percentage of residential greenness, as a proportion of all land-use types with home location buffered at 300m and 1000m were estimated for England residents using the 2005 Generalized Land Use Database for England | Population-based cohort study  n=375,342  Median follow-up 8.9 years | UK adults aged 40-69 (at baseline in 2006-2010) enrolled in the UK Biobank Study | First record of all-cause, AD, and/or vascular dementia from linked primary and secondary care health records, or mortality records. Using ICD and Read codes | Cox regression models adjusted for age, sex, education, ethnicity, smoking status, sleep duration, vegetables consumption, fruit consumption, fish consumption, obesity, deprivation quintiles, and APOE4 status. Mediation analysis for air pollution (PM2.5, PM10) | Increased greenspace exposure (300m buffer) associated with lower risk of all-cause dementia: HR 0.97 (0.94,1.00) per IQR increment, with similar results for 1Km buffer. Similar, significant findings for vascular dementia, 300m buffer. But otherwise null associations for vascular dementia 1000m buffer and for AD (either buffer). Air pollution found to mediate up to 66.6% of the association |
| Klompmaker, 2022 ^32^ | Greenness, parks, blue spaces.  Greenness (normalized difference vegetation index), percentage park cover, and percentage blue space cover (surface water ≥1.0% vs <1.0%) | Community-based cohort study  n=61,662,472  Up to 16-year follow-up | Medicare beneficiaries aged ≥65 from 2000-2016 (open cohort) | First hospitalisation with primary or secondary discharge diagnosis of dementia based on ICD-9 and ICD-10 codes | Cox-equivalent Poisson models adjusted for calendar year, region, US Census zip code–level covariates, county-level smoking status, an off‐set for total person-time, and strata for all possible combinations of age, sex, race and ethnicity, Medicaid eligibility, and follow-up year | Greenness was negatively associated with dementia risk: HR 0.95 (0.94, 0.96) per IQR increase. No association was found for percentage park or blue space cover |
| Rodriguez-Loureiro, 2022 ^33^ | Residential greenness.  Using the 2006 Normalized Difference Vegetation Index within 500m from residence | Population-based cohort study  n=1,134,502  Up to 13 years follow-up | Individuals aged ≥60 years residing in the five largest Belgian urban areas at baseline (2001) | ICD-10 codes from mortality records | Cox proportional hazards models using age as underlying time scale were used adjusted for baseline hazard as a function of age with strata terms for each 5-year categorized age group, gender, migrant background, living arrangement, education, housing tenure, household income, and a frailty term to account for the cluster effects of residing in one of the five largest urban areas | Higher residential greenness associated with a reduced risk of AD: HR 0.95 (0.93, 0.98) per IQR increment. Similar findings for vascular dementia and unspecified dementia. All associations attenuated when models additionally adjusted for air pollution |
| Slawsky, 2022 ^34^ | Residential green space.  Greenspace was defined by three metrics: Normalized Difference Vegetative Index, percent park overlap within a 2km radius, and linear distance to nearest park. Combined into a composite residential greenspace measure categorized into tertiles | Secondary analysis from within a randomised controlled trial  n=3,047  Median follow-up 6.1 years | US adults aged participants aged ≥75 years enrolled in the Gingko Evaluation of Memory Study | Dementia classification was based on DSM-IV criteria | Cox proportional hazards models were used adjusting for year, race, sex, treatment arm, recruitment site, neighbourhood SES, education, alcohol use, smoking, mobility, cognitive status at baseline, BMI, APOE ɛ4 status, and rurality | Mixed findings. Medium residential greenspace was associated with reduced risk of dementia in fully adjusted models (ref low greenspace): HR 0.77 (0.62, 0.96). High residential greenspace was not statistically significantly associated with dementia in fully adjusted models: HR 0.82 (0.63, 1.06) |
| Tani, 2021b ^35^ | Neighbourhood sidewalk coverage. Calculated as the percentage of sidewalk area of road area within 436 residential neighbourhood units using geographic information systems | Population-based cohort study  n=76,053  Mean follow-up 3 years | Adults aged ≥65 years enrolled in the Japan Gerontological Evaluation Study, who were physically and cognitively independent | Dementia incidence was ascertained by linking the participants to the standardized in-home assessment and medical examination conducted under Japan’s public long-term care insurance registry | Multilevel Weibull survival models with the “vce” (cluster) option were used adjusting for age, sex, education, annual income, living situation, marital status, employment status, hypertension, DM, hearing loss, heart disease, stroke, depressive symptoms, ADLs, cognitive complaints, duration of residence, walking time, frequency of going out, use of a car when going out | Increased sidewalk coverage was associated with a decreased dementia risk among participants living in urban areas: HR 0.52 (0.41, 0.68) (highest vs lowest quartile). But not among those living in rural areas: HR 1.27 (0.89, 1.80) |
| **Noise** | | | | | | |
| Cantuaria, 2021 ^36^ | Long term residential exposure to road traffic and railway noise.  Estimates based on prediction models drawing upon height of each address, annual average daily traffic, vehicle distribution, travel speed, and road type; annual average daily train lengths, travel speed, and train types obtained from the Danish state railway network | National registry-based cohort study  n=1,938,994  Mean follow-up 8.5 years | Adults aged ≥60 years living in Denmark between 1 January 2004 and 31 December 2017 | Incident cases of all cause dementia and dementia subtypes AD, vascular dementia, and PD related dementia), identified from national hospital and prescription registries | Cox regression models comparing dementia risk at the most and least exposed facades of buildings based on 10-year mean exposure, adjusted for sex, civil status, occupational status, income, and calendar year, with age as the underlying time | Household exposure to highest road traffic and railway noise at the most exposed façades of buildings (compared to lowest noise at least exposed facade) was associated with a higher risk of all cause dementia: HR 1.16 (1.13, 1.19) (road); 1.16 (1.12, 1.20) (railway). Positive exposure-response associations, with slight levelling off at highest exposures. Stronger associations for Alzheimer’s disease than other subtypes. 11.4% and 3.0% of incident dementia cases in Denmark in 2017 attributable to road and railway noise exposure, respectively |
| **Geography** | | | | | | |
| Gilsanz, 2017 ^37^ | Being born in a US state with high stroke mortality (typically Southern states); race (black or non-Black) | Community-based cohort study  n=7,423  Mean follow-up (starting in 1996, see outcome ascertainment) 11.6 years | Mid-life users of the Kaiser Permanente Northern California (USA) healthcare system who attended an optional health check-up between 196 and 1973 | Dementia diagnoses obtained from electronic health records from 1996 – 2015, using ICD-9 codes for AD and dementia | Cox proportional hazards models, adjusting for age, sex, race, education, midlife vascular risk factors (BMI, smoking duration, and hypertension status), and late-life cardiovascular risk factors (DM, hypertension, heart failure, acute MI, and stroke) | Birth in an HSMS was associated with a higher risk of dementia: HR 1.27 (1.11, 1.44). Compared with nonblack persons born outside of an HSMS, black individuals born in an HSMS had the highest dementia risk: HR 1.48 (1.31, 1.68), followed by nonblack persons born in an HSMS: HR 1.43 (1.20, 1.70), and black individuals not born in an HSMS: HR 1.32 (1.13, 1.54) |
| Li, 2022 ^38^ | Living in an urban or rural area.  Not defined | Population-based cohort study  n=8,221  Cross-sectional | Adults aged ≥65 from The Hubei Memory & Aging Cohort Study in Central China | Dementia diagnosis based on DSM-4 criteria was a result of consensus of an expert panel after reviewing participants’ functional, neurological, and cognitive abilities | Standardized prevalence rates and logistic regression models adjusting for gender, age, education level, living arrangements, cigarette smoking, drinking, and social connections | Age-standardised dementia prevalence rate in rural areas was 2.65 (0.73, 1.03) times higher than urban regions. In logistic regression models, dementia risk was lower among men in rural areas (ref: rural women): OR 0.65 (0.46, 0.92); and higher for low education groups in urban areas only (ref: urban high education): OR 2.61 (1.39, 4.91) |
| **Air Pollution** | | | | | | |
| Li, 2024 ^39^ | Household solid cooking fuel.  Self-reported main source of cooking fuel was classified into cleaner fuel (including natural gas, liquefied petroleum gas, or electric) and solid fuel (including coal, crop residue, wood, and solid charcoal) | Population-based cohort study  n=1,379  Up to 3 years of follow-up | Participants aged ≥65 years from the China Health and Retirement Longitudinal Study | Dementia diagnosis was based on the Community Screening Instrument for Dementia, which consists of seven cognitive tests and informant-based changes in daily functioning | Retrospective logistic regression analyses were conducted adjusting for age, education, urbanity, drinking, self-report hearing, peak expiratory flow, difficulties in ADLs, depression, social isolation, global cognition, and BMI | Solid cooking fuel use was associated with an increased dementia risk: OR 1.44 (1.06, 1.96) (ref clean fuel). Change in  cooking fuel from solid to clean (ref clean throughout) associated with increased risk: OR 1.64 (1.07, 2.51). Null association for transition from clean to solid fuel |
| **Other specific exposures** | | | | | | |
| Martinez, 2021 ^40^ | Agent Orange exposure.  Required both patient self-report of Vietnam service with exposure to Agent Orange and a clinician indicator that a health care encounter was associated with Agent Orange exposure | Cohort study  n=316,351  Mean follow-up 5.5 years (max 14) | A random 2% sample of US veterans of the Vietnam era who received inpatient or outpatient Veterans Health Administration care | Dementia was defined from linked healthcare records, using specific ICD-9 codes | Fine-Gray competing risk models were used adjusting for race, sex, education, income, DM, hypertension, MI, CVD, TBI, PD, mood disorder, anxiety, substance use disorder, PTSD, tobacco use, and sleep disorder | Agent Orange exposure associated with increased risk of dementia: HR 1.68 (1.59, 1.77). Veterans with Agent Orange exposure also developed dementia at a mean of 1.25 years earlier |
| Zeng, 2023 ^41^ | Occupational radon exposure.  Radon exposure (1915–1988) was assessed using two job-exposure matrices constructed from historical records and expressed in working level months | Retrospective, occupational cohort study  n=34,536  Up to 26 years of follow-up | Male miners with work experience in multiple ore types in Ontario, Canada | Dementia diagnoses were ascertained using hospital discharge or ambulatory care data (ICD-9 and ICD-10 codes) | Poisson regression models were used to estimate incidence RR between cumulative radon exposure in working level months and each neurodegenerative outcome adjusted for age, age*age, birth year throughout the follow‐up period, and self‐reported McIntyre Powder (respirable aluminium) exposure | No statistically significant associations were found for all-cause dementia across any exposure level or any ore type. For AD, those with slightly raised exposure (1-5 working level months) was associated with a higher risk compared to those with <1 working month: RR 1.23 (1.05, 1.45). But all higher exposure categories reported null associations |
| Zeng, 2021 ^42^ | Occupational aluminium dust exposure.  Respirable aluminium exposure through McIntyre Powder (binary annual classification), assessed by self-reports and historical records | Retrospective, occupational cohort study  n=36,826  Median follow-up 23 years | Male miners with work experience in multiple ore types in Ontario, Canada | Dementia diagnoses were ascertained using hospital discharge or ambulatory care data (ICD-9 and ICD-10 codes) | Poisson regression models were used adjusted for age, age*age, and birth year throughout the follow-up period | Increased all-cause dementia risk for those ever exposed: RR 1.12 (1.06, 1.19). Evidence of a dose-response relationship and higher risk for more recent exposure. Null associations with AD risk |
| Zhou, 2021 ^43^ | Childhood second hand smoke exposure.  Parental smoke exposure was defined as 1) the presence of parental smoking (either parent) of greater than 0 mean packs/day at any point in an examination period when his or her offspring participant was between 0 and 18 years of age, and 2) the summation of the number of cigarettes smoked daily by both the mother and father (categorized into no exposure, 0-1 pack/day and >1 pack/day) | Population-based cohort study  n=2,993  Median follow-up 31 years | Participants of the Framingham Offspring cohort with at least 1 parent in the Framingham Heart Study original cohort with a known smoking status at any point until his or her offspring reached the age of 18 years | Dementia diagnosis was made according to the DSM-IV. AD dementia diagnosis was based on the criteria of the National Institute of Neurological and Communicative Disorders and Stroke and the AD and Related Disorders Association for definite, probable, or possible AD | Cox proportional hazards models were applied adjusted for age, sex, hypertension, smoking, DM, and BMI | Parental smoking greater than 1 pack/day was associated with an increased risk of dementia compared with individuals with no exposure: HR 2.86 (2.00, 4.09). Similar results for AD specifically. Results for smoking between 0 and 1 pack/day were also significant: HR 1.79 (1.21, 2.64) |
| **Housing and Sanitation** | | | | | | |
| Roncarati, 2024 ^44^ | Homelessness and housing instability.  Assessed using ICD-10 codes | Case-control study  n=88,388  Median follow-up 9 years | US Veterans aged ≥50 | Dementia diagnosis based on ICD-10 codes from linked veterans’ healthcare data | Cox proportional hazard models were used adjusting for age, race, marriage status, combat Veteran, rurality, rheumatic disease, renal disease, liver disease, DM, hypertension, heart failure, pulmonary disease, valvular disease, cerebrovascular accident, TBI, alcohol and drug use disorder, depression, PTSD, psychoses, hospice use, and nursing home use | Experiencing housing instability associated with a higher dementia risk compared to veterans in stable housing: HR 1.41 (1.36, 1.47) |
|  |  |  |  |  |  |  |
| **Income, employment, and poverty** | | | | | | |
| **Occupation (general)** | | | | | | |
| Appel, 2022 ^45^ | Occupation and education.  Longest held position (of at least 3 years) between age 30 and 55. Years of formal education (>12 years, 10-12 years, <10 years) | Registry-based cohort study  n=1,210,720  Mean follow-up 10.5 years | Entire Danish population, excluding those who emigrated, those unemployed, and those assisting a self-employed spouse | Incident dementia defined by ICD-8 codes and anti-dementia medication ascertained from record linkage with in-patient, prescribing, research, and mortality datasets | Aalen’s additive hazards model adjusted for age, birth cohort, sex, marital status, cognitive ability (at 18), and Charlson Comorbidity Index | Each decrease in educational attainment and occupation-based SEP level was associated with higher dementia risk in a dose–response manner (14.8 and 10.9 additional dementia cases per 100,000 PY, respectively). Higher occupation-based SEP partly mitigated the higher dementia risk associated with lower educational attainment |
| Hyun, 2022 ^46^ | Occupational complexity and education.  Measures of occupational complexity were harmonized across countries using the International Standard Classification of Occupations (ISCO)-08. Educational attainment (highest qualification achieved) | Harmonised multi-community-based cohort study  n=10,195  Up to 6.4 years of follow-up (from data capture, longer from actual exposure) | Participants aged 58-103 years from seven studies included in the COSMIC collaboration representing 6 countries over 4 continents | Dementia diagnosis using DSM-IV criteria or Clinical Dementia Rating scale | Accelerated failure time model with a Weibull distribution controlled for sex and baseline age, late-life cardiovascular comorbidities, education, and APOE ε4 status. With causal mediation analysis | The meta-analytic estimate indicated that high occupational complexity, compared to low complexity, was associated with a 19% (5, 33) increase in dementia-free survival. 28% of the effect of education on dementia risk was mediated by occupational complexity |
| Hyun, 2020 ^47^ | Occupational complexity and ethnicity.  Occupation complexity was derived from the Dictionary of Occupational Titles. Race/ethnicity was categorized as non-Hispanic White, African American, and other | Population-based cohort study  n=1,079  Mean follow-up 4.6 years | Adults aged ≥70 enrolled in the Einstein Ageing Study in New York, USA | Dementia diagnosis was based on standardized clinical criteria from the DSM-IV | Cox proportional hazards models adjusted for retirement age, sex, and ethnicity, education, income, vascular and other comorbidities | Moderate-to-high levels of occupational complexity, compared to lower levels, was associated with lower dementia risk: HR 0.63 (0.43, 0.93) but this effect slightly attenuated when education was added and lost significance when further adjusted for income and comorbidities, though neither of these variables were themselves significant. Stratified by ethnicity, this association remained statistically significant only among African Americans: HR 0.35 (0.16, 0.76) |
| Ishtiak-Ahmed, 2019 ^48^ | Prolonged and serious conflicts at work.  Self-reported prolonged or serious conflicts with a supervisor or colleagues at work | Population-based cohort study  n=6,436  Mean follow-up 17.2 years | Danish adults aged ≥60 at the end of follow-up in the Copenhagen City Heart Study | Dementia diagnoses were extracted from three Danish national registers using ICD-8 and ICD-10 codes | Poisson regression adjusted for age, sex, time since baseline assessment, calendar year, educational attainment, psychiatric disorders, and private life social relations | No association between conflict (vs no conflict) and dementia risk: IRR 1.53 (0.77, 3.03). Also null effects in sex-stratified analyses |
| Ishtiak-Ahmed, 2018 ^49^ | Social relations at work.  Self-reported 1) possibilities to be in contact with coworkers, 2) get along with coworkers, and 3) satisfaction with supervisor | Population-based cohort study  n=1,572  Mean follow-up 15.8 years | Danish men, employed during midlife, aged ≥60 at the end of follow-up in the Copenhagen Male Study | Dementia diagnoses were extracted from three Danish national registers using ICD-8 and ICD-10 codes | Poisson regression adjusted for age, time since exposure measurement, calendar year, marital status, children living at home, educational attainment, job control, work pace, monotonous work, and all three measures of social relations at work | Mostly null findings. Limited possibilities for social contact with co-workers (compared to good possibilities) associated with higher dementia risk :IRR 2.74 (1.23, 6.11). But having some possibilities, or working alone null. Getting along with co-workers and satisfaction with supervisor also null associations |
| Kivimaki, 2021 ^50^ | Cognitive stimulation at work.  Categorised into; low (low demands and low control), medium (high control and low demands or high demands and low control), and high (high demands and high control), using a job exposure matrix indicator that captures any changes of job stimulation based on ISCO codes | Harmonised multi-cohort study  n=107,896  Up to 30.1 years of follow-up | Adult participants enrolled in seven European community-based cohorts | Dementia was identified through linked electronic health records and/or repeated clinical examinations | Cox proportional hazard models adjusted for age, sex, education, smoking, heavy alcohol consumption, physical inactivity, job strain, obesity, hypertension, DM, heart disease, and stroke | The risk of dementia was found to be lower for participants with high compared with low cognitive stimulation at work: HR 0.82 (0.68, 0.98) |
| Ko, 2022 ^51^ | Occupational attainment.  Assessed using the UK Standard Occupational Classification system to assess professional skill and academic level based on self-reported occupational history | Mendelian randomisation study  n=248,847 | UK adults aged 40-69 (at baseline in 2006-2010) enrolled in the UK Biobank Study | European summary statistics for AD were obtained from the International Genomics of Alzheimer’s Project meta-analyses | Multivariable Mendelian randomisation analyses | High (compared to low) occupation levels were associated with reduced risk of AD: OR 0.78 (0.65, 0.92) using the inverse variance weighted method; and OR 0.73 (0.57, 0.92) with the weighted median method. Occupational attainment had an independent effect on the risk for Alzheimer's disease even after taking educational attainment into account: OR 0.72 (0.54, 0.95); OR 0.68 (0.48, 0.97) using either method, respectively |
| Martin-Bassols, 2023 ^52^ | Cognitive activity at work.  Occupations were recorded following the Standard Occupation System codes, which were transformed to 41 Census Occupational Categories that were included in a factor analysis eliciting orthogonally rotated factors | Population-based cohort study  n=9,994  Mean follow-up 10 years | Male participants aged 60 to 85 and enrolled in the US Health and Retirement Study | Dementia diagnosis was based on the Langa-Weir Classification of Cognitive Function that included proxy's assessment of the respondent's memory and problems with ADLs and interviewer's assessment of the respondent's ability to answer survey questions | Multivariate regression analyses adjusted for age, ethnicity, place of birth, being a proxy respondent, survey year, years of parental education, educational attainment, and occupation and industry variables | 1 standard deviation increase in the cognitive activity of longest held occupation was associated with a 0.9 percentage point reduction in predicted dementia risk (confidence intervals not reported, but p-value <0.001); equating to a 24% relative reduction |
| Nabe-Nielsen, 2021 ^53^ | Occupational physical activity.  Occupational physical activity was assessed with the question “Is your current work…?” with the response options: (1) Sedentary (reference group), (2) Light physical work, and (3) Heavy physical work | Population-based cohort study  n=4,721  Mean follow-up 28 years | Danish men aged 40-59 enrolled in the Copenhagen Male Study who worked for one of 14 large private/public companies in the Copenhagen area | Dementia was identified through national registers and defined as being diagnosed with dementia (ICD codes), being registered with dementia as cause of death, or having purchased one of the drugs used in the treatment  of dementia | IRR adjusted for SES, marital status, psychological stress, calendar period, and exposure age | High occupational physical activity exposures was associated with a higher risk of dementia: IRR 1.48 (1.05, 2.10) compared to participants in sedentary jobs. This held even if leisure time physical activity was additionally adjusted for. Null findings for the light physical work group |
| Raggi, 2022 ^54^ | Occupational social class.  Assessed by the British Civil Service grade of employment, a 6-level variable ranging from high (administrative grades) to low (support grades) position | Occupation-based cohort study  n=9,951  Median follow-up 31.6 years | UK Civil Servants, aged 35-55, participating in the Whitehall II study | All-cause dementia was identified based on ICD-10 codes through linkage to secondary care, community mental health services, and mortality records | Cox regression including smoking as a mediator with time as timescale, adjusted for age at baseline, sex, ethnicity and education | Not fully reported. Compared to the highest occupational SES group, those in the lowest group had an increased dementia risk with HRs around 2.0. Evidence of 7-16% mediating effect of smoking history |
| Sundström, 2020a ^55^ | Retirement.  Age at retirement was retrieved from Statistics Sweden | National registry-based study  n=63,505  Mean 20.9 years of follow-up | Swedish citizens born in 1930 who were alive and had some income in 1990 | Dementia diagnosis (ICD codes) was based on data provided by the Swedish National Patient Register and the Cause of Death Register | Competing risk regression models were used adjusting for sex, education, marital status, occupation, and CVD | Increasing age at retirement: HR 0.98 (0.97, 1.00) per year, and later-than-average retirement age (≥66 years): HR 0.35 (0.24, 0.49) were associated with decreased dementia risk |
| Sundström, 2020b ^56^ | Mental demands at work.  Self-reported main occupation coded according to Swedish occupational classification and then linked to the Occupational Information Network database. A composite score for mental demands at work was created by summing all 21 variables reflecting level of cognitive ability needed to perform the job (range 0-7) | Population-based cohort study  n=1,277  Mean follow-up 11 years | Participants aged 60-85 enrolled in the Betula project (randomly invited group from the population register in the Umea municipality, Sweden | Dementia diagnoses were classified according to the DSM IV criteria based on analysis of neuro-psychological testing, structured interviews conducted by trained nurses, observations made at each test occasion, and evaluations of medical records done five yearly | Cox hazard models were used adjusting for age, gender, education, smoking, alcohol, CVD, and Apolipoprotein E status | No association between mental demands at work and incidence of dementia was found HR 0.99 (0.98, 1.01) |
| Zhao, 2023 ^57^ | Physicality of work, sedentary work, and shift work.  Self-reported phenotypes | Mendelian randomisation study  n=263,615 | UK adults aged 40-69 (at baseline in 2006-2010) enrolled in the UK Biobank Study | A large GWAS meta-analysis from the International Genomics of Alzheimer’s Project | Two-sample Mendelian randomization that used inverse variance weighted, MR-Egger, and weighted median | Jobs involving heavy manual or physical work associated with increased AD risk: OR 2.13 (1.36, 3.36), as were jobs that involve mainly walking or standing OR 1.74 (1.19, 2.54), and jobs involving shift work OR 2.78 (1.14, 6.80) |
| **Shift work** | | | | | | |
| Liao, 2022 ^58^ | Shift work.  Shift work (schedule falling outside of 9 am to 5 pm) was self-reported at baseline and categorized into non-shift workers or shift workers. Shift work was further categorized as night shift (work schedule that involves working through the normal sleeping hours) or non-night shift | Population-based cohort study  n=170,722  Median follow-up 12.4 years | UK adults aged 40-69 at baseline (2006-2010), employed, and enrolled in the UK Biobank study | Dementia diagnosis was based on electronic health records including hospital admissions and death registry, using ICD-9 and ICD-10 codes | Cox proportional hazard regression models adjusted for age at baseline, sex, ethnicity, education, and SES | Shift work associated with increased dementia risk: HR 1.30 (1.08, 1.58). No evidence that night shift working was associated with an increased risk compared to non-night shift working. No specific associations with AD or vascular dementia |
| Ling, 2023 ^59^ | Night shift work.  Shift work (schedule falling outside of 9 am to 5 pm) and night shift (work schedule that involves working through the normal sleeping hours) was self-reported at baseline and categorized into no shift workers, irregular shift workers (including shift work and never/ rarely/ sometimes/ usually night shift work), and always night shift workers (always night shift work) | Population-based cohort study  n=245,570  Median 13.1 years follow-up | UK adults aged 40-69 at baseline (2006-2010), employed, and enrolled in the UK Biobank study | Dementia diagnosis was based on electronic health records including hospital admissions and death registry, using ICD-9 and ICD-10 codes | Cox proportional hazards model adjusted for age, sex, BMI, education, neighbourhood SES, ethnicity, smoking, alcohol, hypertension, hypercholesterolaemia, stroke, MI, DM, chronotype, and sleep duration | Both irregular and always night shift working were associated with increased dementia risk: HR 1.20 (1.03, 1.40) and HR 1.47 (1.06, 2.03), respectively. Similar findings, though not all significant, for the association with AD |
| Ren, 2023 ^60^ | Night shifts.  Shift work was defined as a schedule outside of the working hours from 9:00 am to 5:00 pm. Answer options were never/rare, sometimes, usually, and always. Those reporting some shift work additionally reported whether their work involved night shifts (a schedule including working through normal sleeping hours (12:00 am to 6:00 am) | Population-based cohort study  n=276,059  Median 9 years of follow-up | UK adults aged 40-69 at baseline (2006-2010), employed, and enrolled in the UK Biobank study | Dementia was defined using UK Biobank baseline assessment data, hospital inpatient records, and death register data using ICD-9 and ICD-10 codes | Cox proportional hazards models were used adjusted for age, sex, ethnicity, education level, household income, neighbourhood deprivation, BMI, hypertension, DM, CVD, cancer, high cholesterol, depression, anxiety, sleep apnoea, antihypertensive drug use, lipid-lowering drug use, aspirin use, non-aspirin NSAID use, sleep duration, chronotype, APOE ε4 carrier status, smoking, alcohol, physical activity, and diet | Compared with day workers, shift workers had increased dementia risk, with some evidence for further increased risk for those reporting night shifts: HR 1.12 (1.01, 1.37) for never/rare nights, HR 1.24 (1.10, 1.48) for some/usual nights, and 1.35 (1.22, 1.61) for always nights, p-value for trend <0.001 |
| **Occupations (specific)** | | | | | | |
| Arora, 2021 ^61^ | Agricultural work.  Longest-held job in the agricultural sector defined as farmer/ forestry/ fishing in the occupational classification system and agriculture/ forestry/ fishing/ hunting in the industrial classification system | Population-based cohort study  n=12,991  Follow-up length not reported, but comparing midlife employment and late-life dementia | US adults aged ≥65 years enrolled in the Health and Retirement Study | Dementia prevalence according to neuropsychological and clinical assessment as well as expert clinical adjudication | Multiple logistic regression adjusted for age, gender, race, ethnicity, education, marital status, non-housing wealth, rural/urban location, census region, region of birth, years of parental education, and self-rated childhood SES | Longest-held job in agriculture was associated higher dementia risk compared to other jobs: OR 1.46 (1.13, 1.89) |
| Power, 2023 ^62^ | Military employment.  Military employment was self-reported and defined as having a first or second-longest occupation with the military | Population-based cohort study  n=4,370  Mean 8.7 years of follow-up | US adults aged ≥65 years participating in the longitudinal Adult Changes in Thought cohort study | Dementia diagnosis according to the DSM-IV criteria was based on clinical examination and additional cognitive testing | Cox proportional hazard models were used adjusted for age, gender, ethnicity, education, APOE e4 allele status, height, and childhood SES | Military employment was not significantly associated with risk of dementia: HR 1.01 (0.78, 1.29) or risk of AD |
| **Income** | | | | | | |
| Jain, 2022 ^63^ | Low income.  Assessed as dual-eligible Medicare-Medicaid status at any time during the 12 months prior to an intensive care admission | Clinic-based cohort study  n=382  Median follow-up 0.37 years | Any participant in the US National Health and Aging Trends Study who was admitted to intensive care between 2011-2017 who attended a post-intensive care follow-up interview | Transition from no or possible to probable dementia according to the National Health and Aging Trends Study classification based on self- or proxy-reported physician diagnosis, interview of proxy respondents, and score on neuropsychological tests | Multivariable logistic regression adjusted for age, sex, ethnicity, living alone, education, multimorbidity, mechanical ventilation, hospital length of stay, baseline cognition status, and depression | Medicaid eligibility was associated with an increased likelihood of transitioning to probable dementia: OR 9.79 (3.46, 27.65) |
| Keohane, 2023 ^64^ | Income and ethnicity.  Self-reported annual household income grouped into <$15,000, $15–24,999, $25,000–49,999, >$50,000. Self-reported ethnicity grouped into non-Hispanic Whites and non-Hispanic Blacks | Population-based cohort study  n=18,835  Mean follow-up 4.3 years | US adults aged ≥65 enrolled in the Southern Community Cohort Study with >12 months Medicare enrolment | First appearance of a dementia diagnosis in Medicare claims data using ICD9 and ICD-10 codes | Multivariable Cox proportional hazard models adjusted for age, sex, and calendar year, race, education, employment status, marital status, household size, tobacco and alcohol use, self-reported diagnoses, and BMI | Mixed and inconsistent findings.  Unadjusted incidence rates at least twice as high among those with incomes less than $15,000 vs $50,000 or more. Higher unadjusted dementia risk for Black participants than White participants. In fully adjusted, income-stratified models, mostly null associations between ethnicity and dementia risk were reported. In the $15-25k income strata, risk was lower for Black participants than White participants: HR 0.78 (0.62, 0.98) |
| **Neighbourhood Deprivation** | | | | | | |
| Becceril, 2023 ^65^ | Neighbourhood deprivation and race.  Area Deprivation Index based on American Community Survey data, generated from area-level indicators of education, employment, housing-quality, and poverty. Self-identified race/ethnicity as American Indian or Alaska Native, Asian, Hispanic, Non-Hispanic Black, and Non-Hispanic White | Primary care-based cohort study  n=253,421  Up to 10 years of follow-up | People aged >60 in Northeast Ohio, US attending primary care services | Dementia diagnosis according to ICD-10 codes from healthcare and mortality records | Fine-Gray sub distribution hazard regression comparing area deprivation index or race/ethnicity, adjusting for age, sex, depression, anxiety, obesity, hyperlipidaemia, hypertension, DM, coronary artery disease, chronic kidney disease, peripheral vascular disease, CVD, heart failure, alcohol abuse, smoking, | Compared to least deprived neighbourhood, all other quintiles had higher dementia risk. For most deprived quintile:  HR 1.36 (1.25, 1.48). In mutually adjusted models, compared to White ethnicity, Black, Hispanic, and Native American ethnicity also all associated with significant increased dementia risk (HR 1.54, 1.52, and 2.41, respectively). No significant association for Asians |
| Choi, 2024 ^66^ | Neighbourhood social environment.  Made up of (1) socioeconomic deprivation, (2) disorder, and (3) social cohesion. (1) A composite of: % of households on public assistance, % of persons with below poverty level income, % unemployed, % of women-headed households with children, and % without high-school degree. (2) Interviewers’ ratings of whether visible signs of physical and social disorder (e.g. vandalism, trash, drug dealing) were reported around a respondent’s housing. (3) Self-reported levels of solidarity and trust towards neighbourhoods and neighbours | Population-based cohort study  n=9,251  Median 9 years of follow-up | US adults aged >55 enrolled in the Health and Retirement Study | Dementia was assessed using the Langa–Weir classification containing proxy and self-reports and neuropsychological assessment | Separate Cox proportional hazards models compared binary categories of each exposure adjusted for age, sex, race/ethnicity, education, household wealth, employment status, cardiometabolic abnormalities, and cognitive status, including inverse probability of treatment weighting, as well as imputation and proxy status | High neighbourhood socioeconomic deprivation (compared to low) was associated with higher dementia risk: HR 1.18 (1.02, 1.38); as was any (compared to no) neighbourhood disorder: HR 1.27 (1.03, 1.59). Null association for neighbourhood social cohesion. Minimal evidence of mediation through social isolation |
| Dai, 2023 ^67^ | Neighbourhood deprivation.  Measured using the Townsend Index via participants’ addresses | Genetic correlation and Mendelian randomisation study within a population-based cohort  n=455,815 | UK adults of European ancestry, aged 40-69 years at baseline (2006-2010), enrolled in the UK Biobank study | AD identified by a genome-wide meta-analysis for clinically diagnosed AD case-control status and AD-by-proxy status | Genetic correlation and Mendelian randomization | There was a positive genetic correlation (ȓg=0.211) between neighbourhood SES and AD. One genetically predicted standard deviation elevation in neighbourhood SES associated with 18.5% (1.6, 38.2) increase in AD risk |
| Park, 2024 ^68^ | Economic disadvantage.  Lowest income group were medical aid beneficiaries, with the remainder of the cohort split into four levels of income (bottom 20%, 30-50%, 60-80%, top 20%) | Clinic-based cohort  n=16,780  Up to 2 years of follow-up | Those in the Korean National Health Insurance Database who had a ≥2-day intensive care unit admission between 2003 and 2019 | Dementia was determined using specific diagnostic codes (G30, G31) and prescription of certain medications (rivastigmine, galantamine, memantine, or donepezil) | Cox proportional hazard models adjusted for age, sex, region, length of stay, medical utilization, invasive treatments, comorbidity, readmission, primary discharge diagnosis, and type of hospitals | Those in receipt of medical aid (the lowest income group) had a higher dementia risk than those with higher incomes: HR 1.23 (1.04, 1.46), with some evidence of a U-shaped relationship for the four income groups (such that the bottom and top 20% groups had slightly higher incidence rates) |
| **Composite or life course assessments of SES** | | | | | | |
| Geraets, 2023 ^69^ | Life course SES and sex/gender.  Childhood SES was assessed with self-reported childhood deprivation and occupational attainment of the family breadwinner. Adulthood SES was assessed with self-reported occupational attainment and wealth | Population-based cohort study  n=8,941  Up to 11 years of follow-up | English adults aged ≥50 enrolled in the English Longitudinal Study of Ageing | Incidence of dementia was assessed by use of a combined algorithm of (1) self- or informant-reported physician-diagnosis of dementia or AD or (2) an average score of ≥ 3.38 on the16-item Informant Questionnaire on Cognitive Decline in the Elderly | Cox proportional hazard regressions adjusted for age, sex/gender, wealth, and clustering at the household level. | Dementia risk was higher among those who experienced  childhood deprivation (compared to no deprivation): HR 1.51 (1.17, 1.96), low occupational attainment (compared to high): HR 1.60 [1.23, 2.09), and low wealth (compared to high): HR 1.63 (1.26, 2.12). Null associations for parental occupation and for sex/gender |
| Hudomiet, 2022 ^70^ | Lifetime earnings, educational attainment, ethnicity, migrant status.  Lifetime earnings based on an individual’s 35 highest years of earnings. Self-reported ethnicities: non-Hispanic White, non-Hispanic Black, Hispanic, non-Hispanic other | Population-based cohort study  n=21,422  Up to 16 years of follow-up | US adults aged ≥65 enrolled in the Health and Retirement Study | Trends in dementia are primarily based on wave-to-wave differences in the averages of individuals’ performance on cognitive tests, but using an algorithm calibrated using a subgroup with  clinical diagnosis of cognitive tests to measure dementia | Dementia prevalence was estimated by a fitted linear time trend adjusting for the distributions of age, education, income, race and ethnicity, foreign-born status, marital status, hypertension, DM, stroke, and heart problems | Dementia prevalence rates dropped during the period of interest (2000-2016). Improvements in educational attainment accounted for half of this decrease. Prevalence of dementia tended to be higher among those with low lifecourse earnings, lower education, ethnic minority individuals, and for those not born in the US. These differences generally narrowed over time |
| Klee, 2023 ^71^ | Neighbourhood and individual-level deprivation.  Area-level deprivation was based on the Townsend Deprivation Index. Individual-level socioeconomic deprivation based on car and home ownership, housing type and income | Population-based cohort study  n=196,368  Median follow-up 8.0 years | UK adults aged ≥60 enrolled in the UK Biobank study | Dementia was ascertained using linked healthcare and mortality records, using ICD-9 and ICD-10 codes | Cox proportional-hazards regressions were applied adjusting for age, education, sex, marital status, ancestry, in-sample third-degree relatedness, depressive symptoms, and healthy-lifestyle score | High area-level: HR 1.28 (1.14, 1.43) and individual-level: HR 2.38 (1.98, 2.87) deprivation were independently associated with an increased risk for dementia compared to low-to-moderate level, irrespective of genetic risk |
| Korhonen, 2023 ^72^ | Childhood and adulthood socio-economic status.  Index calculated from various childhood SES indicators including: parental education, parental occupational social class, home ownership, geographical region, single parent, household crowding, housing standard. Adulthood SES from educational attainment, occupational social class, and income | Population registry-based study  n=95,381  Up to 19 years of follow-up (from data collection) | Children living in Finnish families at the age of 0–15 at the time of the 1950 Finnish population census and who were alive and lived in the community at the age of 49–64 years at the end of 1999 | Dementia was identified from hospital, medication reimbursement and death registers that cover the whole population using ICD-10 codes and anti-dementia medication | Discrete time survival analysis using logistic regression and mediation analysis applying the Karlson–Holm–Breen method, adjusted for age, gender, region of residence in 1950, calendar year, and childhood characteristics (with exception of childhood socio-economic index) | Mostly null findings. Household crowding (≥4 per heated room Vs. <2) was associated with an increased dementia risk: OR 1.18 (1.10, 1.28). Father only households (ref: two parents) also associated with increased risk: OR 1.26 (1.06, 1.50). Adulthood SES mediated 47-65% of household crowding association, but only 14% of single-father association |
| Lai, 2023 ^73^ | Individual-level socioeconomic position.  Based on a latent class model that divided the cohort into tertiles based upon a composite score of: (1) educational attainment (International Standard Classification of Education), employment status (employed or self-employed, retired, and others), and average total household income before tax (quintiles) | Population-based cohort study  n=331,066  Mean follow-up 12 years | UK adults aged 40-69 at baseline (2006-2010), enrolled in the UK Biobank study | Diagnosis of incident dementia was ascertained through hospital admissions data from ICD-10 codes | Cox proportional hazard regression models were used adjusting for age at baseline, sex, ethnicity, healthy lifestyle score, number of leisure and social activities per week, hearing difficulty, abdominal obesity, frailty, urbanicity, neighbourhood deprivation, and biological age | Compared to the high socioeconomic position tertile, both the medium (HR 2.59 (2.29, 2.92)) and low (HR 2.58 (2.28, 2.91)) tertiles were associated with increased dementia risk. Similar findings for AD diagnoses specifically |
| Li, 2023 ^74^ | Individual-level socioeconomic position.  Based on a latent class model that divided the cohort into tertiles based upon a composite score of: (1) educational attainment (International Standard Classification of Education), employment status (employed or self-employed, retired, and others), and average total household income before tax (quintiles) | Population-based cohort study  n=294,133  Median follow-up 12.5 years | UK adults aged 40-69 at baseline, enrolled in the UK Biobank study. Early-onset (age <65) analyses included those aged <60 at baseline and late-onset (age ≥65) included those aged ≥65 at the end of follow-up | Dementia diagnosis was based on electronic health records including hospital admissions and death registry, using ICD-9 and ICD-10 codes | Multivariable Cox proportional-hazard regression models adjusted for age, sex, ethnicity, assessment centres, social isolation, sleep quality, depressive symptoms; presence of APOE ε4, BMI, hypercholesterolaemia, DM; prevalent comorbidities (hearing difficulty, hypertension, and CVD), family history of dementia, and lifestyle (smoking, alcohol, physical activity, and diet) | Low (vs high) socioeconomic position was associated with an increased risk for early-onset dementia: HR 3.38 (2.61, 4.37) and late-onset dementia: HR 1.70 (1.55, 1.86). No interaction with, and minimal mediation by, lifestyle |
| Ou, 2024 ^75^ | Socioeconomic status.  Classified by household income, education qualifications, and employment status. An overall latent class SES variable was created that identified three latent classes | Population-based cohort study  n=276,730  Mean 8.5 years of follow-up | UK adults aged 40-69 at baseline (2006-2010), enrolled in the UK Biobank study | Dementia diagnoses were obtained using hospital inpatient records (ICD codes), primary care data, (Read codes), and death register data | Cox proportional hazards analysis was performed adjusted for age at inclusion, sex, ApoE ε4 status, BMI, depression, hypertension, DM, CVD, and area-level SES | Being in the medium and high SES groups was associated with reduced dementia risk (ref: low SES group): HR 0.32 (0.28, 0.38) for high; HR 0.40 (0.34, 0.46) for medium |
| Weiss, 2021 ^76^ | Occupational social class, neighbourhood safety, food security, education, and ethnicity.  Self-reported major occupational category of the longest held job (classified as upper white-collar, lower white-collar, blue-collar, and never worked for pay), self-reported neighbourhood safety (low [fair or poor] vs high [good, very good, or excellent]), and self-reported food security over the past two years (yes/no). Self-reported ethnicity: Black, White, and Hispanic | Population-based cohort study  n=16,234  Median follow-up 15.0 years | Participants of the Health and Retirement Study, US (waves 2000–2016) aged ≥50 years who did not self-report their ethnicity as "other" | Dementia based on Langa–Weir classification containing proxy and self-reports and neuropsychological assessment | A multistate framework to simultaneously evaluate all risk factors was performed while accounting for interval censoring and the semi-competing risk of mortality | Lower occupational social class, and food insecurity were generally associated with increased dementia risk, though non-significant for some strata of gender/ethnicity. Dementia incidence rates higher for non-White groups. Less than high school educational attainment associated with increased dementia risk for all women and white men, but null findings for Black and Hispanic men |
| **Social Inclusion & Discrimination** | | | | | | |
| **Ethnicity, Structural Racism, and Migration** | | | | | | |
| Beydoun, 2022 ^77^ | Ethnicity.  Race/ethnicity was self-identified as non-Hispanic White, Non-Hispanic Black, Mexican-American, other, and Non-White | Population-based cohort study  n=4,592  Up to 26 years of follow-up | US adults aged ≥60 years and enrolled in the  National Health and Nutrition Examination Survey | All-cause dementia defined using ICD-9 codes through linked electronic health records from  Centers for Medicare and Medicaid Services-Medicare data | Cox proportional hazards models were used to test associations between ethnicity and dementia. Mediation from SES (poverty income ratio and years of education), lifestyle (diet, nutritional biomarkers, smoking, alcohol, social support, and physical activity), health, and cognitive performance was further assessed through model attenuation and formal pathway analysis using GSEM models | Black ethnicity was associated with a higher risk of dementia compared to white ethnicity, in models adjusted for age and sex: HR 1.34 (1.15, 1.55). This was attenuated by inclusion of SES in the model. Null associations for Mexicans and other ethnicities. Pathway analysis identified significant pathways from non-white ethnicity to lower SES to lower dementia, with both direct pathways and indirect pathways through poorer diet and less social support |
| Bonnechere, 2023 ^78^ | Ethnicity.  Self-reported, White, Black or Asian | Population-based cohort study  n=272,660  Mean follow-up 11.2 years | UK adults aged 55-69 at baseline (2006-2010), enrolled in the UK Biobank study | Dementia ascertained from self-reported existing diagnosis at the baseline verbal interview, ICD codes from linked electronic healthcare records, or mortality records | Cumulative incidence risk was calculated adjusting for age, sex, education, hearing loss, TBI, hypertension, alcohol, obesity, smoking, depression, social isolation, physical inactivity, DM, PM2.5 exposure, family history of dementia, APOE genotype, and genetic risk score of dementia | Compared to White participants, an increased dementia risk was found for black participants: HR 1.63 (1.22, 2.19) but not for Asian participants |
| Cha, 2022 ^79^ | Ethnicity and sibling loss.  Self-reported ethnicity: non-Hispanic White, non-Hispanic Black, Hispanic. Sibling loss was assessed by a time-varying variable and categorized into no loss (reference), one loss, and multiple losses | Population-based cohort study  n=13,589  Up to 16 years of follow-up | US adults aged ≥65 enrolled in the Health and Retirement Study | Dementia based on Langa–Weir classification containing proxy and self-reports and neuropsychological assessment | Discrete-time hazard regression models adjusted for age, gender, and education | Compared to White ethnicity, Black ethnicity was associated with higher dementia risk: HR 1.70 (p<0.001), as was Hispanic ethnicity: HR 1.49 (p<0.001). Experiencing sibling loss, either one, or multiple, was associated with significant increases in dementia risk (HR 1.46 and 1.57, respectively) |
| Cheung, 2022 ^80^ | Ethnicity.  From national administrative registers, categorised as Māori, Pacific Islander, Asian, Middle East Latin American and African, Other and European | Population-based descriptive study  n=44,136 | New Zealanders aged ≥60 between 2016 and 2020 with a dementia diagnosis | Seven national health data sets which include community, secondary care, prescription, and mortality data | Dementia prevalence was age and sex standardised to the source population. 1-year period prevalence was reported for the 2019/20 period | For those aged ≥60, 1-year standardised dementia period prevalence was 5.4% for Māori, 6.3% for Pacific Islanders, 3.7% for Europeans and 3.4% for Asians. At aged ≥80: 17.5% for Māori, 22.2% for Pacific Islander, 13.6% for European and 13.5% for Asian |
| Fortinsky, 2023 ^81^ | Ethnicity and educational attainment.  Ethnicity: self-report classified as non-Hispanic White, non-Hispanic White, non-Hispanic other, Hispanic. Educational attainment: primary, secondary, or tertiary education | Cross-sectional study  n=3,520 | Adults aged ≥65 years receiving home- and community-based services in Connecticut, USA in 2019 | Diagnosed dementia or Cognitive Performance Scale 2 score ≥4 | Generalized multivariate logistic regression adjusted for participant age group, gender, education, race/ethnicity, depression, Charlson comorbidity index (excluding depression), and Social Vulnerability Index category. | No significant differences in dementia risk by ethnicity. Higher likelihood of dementia for those with only primary education (compared to tertiary): OR 1.82 (1.45, 2.28) |
| Garcia, 2020 ^82^ | Ethnicity and age of migration.  Ethnicity was grouped into US-born non-Latino White, US-born Latino, and foreign-born Latino. Age of migration was grouped into <18 years; 18-34 years; and ≥35 years | Population-based cohort study  n=13,266  Up to 8 years of follow-up | US adults aged ≥65 enrolled in the Health and Retirement Study from 2006-2014 | Dementia based on Langa–Weir classification containing proxy and self-reports and neuropsychological assessment | Multinomial logistic regression models adjusted for age, sex, education, marital status, health insurance status, geographical residence, chronic comorbid health conditions, BMI, smoking, alcohol, and participation rate | In fully adjusted models, compared to US-born Whites, there was no association with dementia for US-born Latinos, or any migrant group |
| Hayes-Larson, 2023 ^83^ | Ethnicity, migrant status, and education.  Self-reported Asian American ethnic group (Chinese, Filipino, Japanese), US-born or foreign-born status, and educational attainment (binary: college degree or not) | Population-based cohort study  n=14,749  Mean follow-up 9.9 years | Adults aged 60-90 members of the Kaiser Permanente Northern California health care delivery system | Incident dementia diagnosis using using ICD-9 and ICD-10 codes in electronic healthcare records | Cox proportional hazards and Aalen additive hazards models with age as timescale, adjusted for sex, and interaction of education with nativity | College education was associated with a lower dementia risk. This association was stronger and only statistically significant in the foreign-born strata. Within this strata, college education was associated with a reduced dementia risk for Chinese and Filipino groups but null associations for the Japanese group |
| Higgins-Tejera, 2023 ^84^ | Ethnicity.  Self-reported ethnicity: non-Hispanic White, non-Hispanic Black, Hispanic | Population-based cohort study  n=9,923 | US adults aged ≥50 enrolled in the Health and Retirement Study | Dementia based on Langa–Weir classification containing proxy and self-reports and neuropsychological assessment | Poisson regression to estimate prevalence ratios, adjusted for age, sex, education, smoking, alcohol, BMI, exercise, sleep, cholesterol, DM, and chronic conditions | The prevalence of dementia was higher for non-Hispanic Black (12%) and Hispanic (8.6%) groups than for the White group (2.7%) (p<0.001). Evidence for a very small amount of this difference being attributable to Cystatin C, secondary to a moderating effect |
| Kornblith, 2022 ^85^ | Ethnicity.  Self-reported ethnicity from healthcare database grouped into: American Indian or Alaska Native, Asian, Black, Hispanic, and White | Clinic-based cohort study  n=1,869,090  Mean follow-up 10.1 years | US adults aged ≥55 receiving care at a US Veterans Health Administration medical center between 1999 and 2019 | Incident diagnosis of dementia according to ICD-9 and ICD-10 codes from healthcare records | Fine-Gray proportional hazards models were used to examine time to diagnosis, with age as the time scale, accounting for competing risk of death, adjusting for adjusted for sex, education, DM, obesity, hypertension, PTSD, alcohol use disorder, TBI, stroke or transient ischemic attack | Compared with White participants, higher dementia risk for Asian participants: HR 1.20 (1.13, 1.28); Black participants: HR 1.54 (1.51, 1.57); and Hispanic participants HR 1.92 (1.82, 2.02). Non-significant findings for American Indian or Alaska Native participants: HR 1.05 (0.98, 1.13) |
| Li, 2023 ^86^ | Ethnicity.  Self-reported ethnicity categorised into: White and non-White | Cross-sectional study  n=9,277 | Community-dwelling adults in the USA aged ≥65 years participating in the 2019 National Health Interview Survey | Self-reported dementia diagnosis | Multivariate logistic regression model controlling for sex, severe visual impairment, hearing difficulty, DM, arthritis, cancer, coronary artery disease, and depression | No association between ethnic minority status and self-reported prevalent dementia: HR 1.12 (0.79, 1.60) |
| Lim, 2022 ^87^ | Ethnicity.  Self-reported ethnicity categorised as African Americans, Native Hawaiians, Japanese Americans, Latinos, Whites, and Filipinos | Population-based cohort study  n=105,796  Median follow-up 8.1 years | Hawaii and Los Angeles County Residents, aged 45-75, participating in the Multiethnic Cohort Study | Linkage to the Centers for Medicare & Medicaid administrative enrolment claims data since 1999 and National Death Index for vital status | Cox proportional hazards regression model adjusted for age at cohort entry, age at Medicare follow-up start, education, heart disease, stroke, DM, hypertension, mean annual Medicare usage, and competing risk for death | Compared to White participants, higher dementia risk for African Americans: women HR 1.22 (1.15, 1.30) and men HR 1.25 (1.15, 1.35). And a lower risk for Filipinos: women HR 0.84 (0.74, 0.95) and men HR 0.71 (0.62, 0.83). Null findings for Japanese-Americans and Latinos, and non-significant evidence of an increased risk for Native Hawaiian participants. Similar findings for specific AD diagnosis. Ethnic differences were less pronounced amongst APOE e4 carriers |
| Mukadam, 2022 ^88^ | Ethnicity.  Self-reported using UK Census categories: White, South Asian, Black, or other | Population-based cohort  n=294,162  Median follow-up 11.8 years | UK adults aged 55-69 at baseline (2006-2010), enrolled in the UK Biobank study | Diagnosis of dementia was established through self-report or linked electronic health records using ICD-9 and ICD-10 codes | Cox regression adjusted for age, sex and neighbourhood deprivation; and additionally, for known modifiable risk factors. Tests for interaction between dementia risk factors and ethnicity | Risk of dementia was higher in Black participants than White participants: HR 1.43 (1.16, 1.77) which was only slightly attenuated by adjusting for modifiable risk factors. No significant differences for South Asian groups compared to White participants. Association between each risk factor and dementia was similar in each ethnic group with no evidence to support any interaction effect |
| Mukadam, 2023 ^89^ | Ethnicity.  Derived from medical records and grouped into: White, South Asian, Black, or other | Clinic-based cohort study  n=662,882  Up to 21 years of follow-up | Adults aged ≥65 years living in the UK | All-cause dementia defined as any diagnostic code or anti-dementia medication recorded in linked electronic primary care, hospital, or mortality records | Estimated IRRs were calculated for South Asian and Black ethnic groups (compared to the White group) by fitting multivariable Poisson regression models with log person-time as an offset. Ethnic differences in age at dementia diagnosis were tested for using linear regression. Cox proportional hazards regression in those diagnosed with dementia with time to death as the outcome to test differences in survival. Analyses were adjusted for age, sex, and multiple deprivation | Dementia incidence was higher in Black people compared to White participants: IRR 1.22 (1.15, 1.30) but similar for South Asian participants. Being from an ethnic minority background was associated with an earlier age at diagnosis, and an earlier age at death, compared to White participants |
| Pohl, 2021 ^90^ | Ethnicity and residential segregation.  Ethnicity was self-reported and categorised into non-Hispanic White, non-Hispanic Black, and Hispanic. Residential segregation was defined across three domains: dissimilarity, isolation, and interaction using data from a previous community survey | Population-based cohort study  n=4,556  Mean follow-up 5.3 years | Adults, aged ≥65, living in Northern Manhattan, New York, USA and enrolled in the Washington Heights Inwood Columbia Aging Project | Diagnosis of dementia was established by a team of neurologists, psychiatrists, and neuropsychologists based on a review of available clinical information, including neuropsychologic and medical patient data | Cox proportional hazards models with random effects at the block group-level were used adjusted for age, sex/gender, ethnicity, childhood SES, years of education, occupation, language of test administration, birthplace, and recruitment cohort | None of the segregation measures were found to have an overall association with increased dementia risk. However, when stratified by the ethnic group experiencing the segregation, there was a more consistent pattern of statistically significantly increased dementia risk for areas where predominantly non-Hispanic Black participants live. Null associations for Hispanic areas (compared to predominantly White areas) |
| Younan, 2022 ^91^ | Ethnicity.  Self-reported and grouped into non-Hispanic White or Black participants | Population-based cohort study  n=34,536  Up to 26 years follow-up | US, community-dwelling women aged 65–79, enrolled in the Women’s Health Initiative Memory Study | AD was defined by DSM-IV | Cause-specific proportional hazard models accounting for death as the competing event, adjusted for age, education, family income, employment status, smoking, alcohol, physical activity, CVD, hypertension, depression, BMI, DM, postmenopausal hormone treatment, and study intervention assignment | AD risk was greater in Black compared to White women: HR 2.26 (1.29, 3.96) |
| Becerril, 2023 ^65^ | Self-identified race/ethnicity as American Indian or Alaska Native, Asian, Hispanic, Non-Hispanic Black, and Non-Hispanic White | Primary care-based cohort study  n=253,421  Up to 10 years of follow-up | People aged >60 in Northeast Ohio, US attending primary care services | Dementia diagnosis according to ICD-10 codes from healthcare and mortality records | Fine-Gray sub distribution hazard regression comparing race/ethnicity, adjusting for age, sex, depression, anxiety, obesity, hyperlipidaemia, hypertension, DM, coronary artery disease, chronic kidney disease, peripheral vascular disease, CVD, heart failure, alcohol abuse, smoking, | In mutually adjusted models, compared to White ethnicity, Black, Hispanic, and Native American ethnicity also all associated with significant increased dementia risk (HR 1.54, 1.52, and 2.41, respectively). No significant association for Asians |
| Gilsanz, 2017 ^37^ | Being born in a US state with high stroke mortality (typically Southern states); race (black or non-Black) | Community-based cohort study  n=7,423  Mean follow-up (starting in 1996, see outcome ascertainment) 11.6 years | Mid-life users of the Kaiser Permanente Northern California (USA) healthcare system who attended an optional health check-up between 196 and 1973 | Dementia diagnoses obtained from electronic health records from 1996 – 2015, using ICD-9 codes for AD and dementia | Cox proportional hazards models, adjusting for age, sex, race, education, midlife vascular risk factors (BMI, smoking duration, and hypertension status), and late-life cardiovascular risk factors (DM, hypertension, heart failure, acute MI, and stroke) | Birth in an HSMS was associated with a higher risk of dementia: HR 1.27 (1.11, 1.44). Compared with nonblack persons born outside of an HSMS, black individuals born in an HSMS had the highest dementia risk: HR 1.48 (1.31, 1.68), followed by nonblack persons born in an HSMS: HR 1.43 (1.20, 1.70), and black individuals not born in an HSMS: HR 1.32 (1.13, 1.54) |
| Hudorniet, 2022 ^70^ | Self-reported ethnicities: non-Hispanic White, non-Hispanic Black, Hispanic, non-Hispanic other | Population-based cohort study  n=21,422  Up to 16 years of follow-up | US adults aged ≥65 enrolled in the Health and Retirement Study | Trends in dementia are primarily based on wave-to-wave differences in the averages of individuals’ performance on cognitive tests, but using an algorithm calibrated using a subgroup with  clinical diagnosis of cognitive tests to measure dementia | Dementia prevalence was estimated by a fitted linear time trend adjusting for the distributions of age, education, income, race and ethnicity, foreign-born status, marital status, hypertension, DM, stroke, and heart problems | Dementia prevalence rates dropped during the period of interest (2000-2016). Prevalence of dementia tended to be higher among ethnic minority individuals, and for those not born in the US. These differences generally narrowed over time |
| Weiss, 2021 ^76^ | Self-reported ethnicity: Black, White, and Hispanic | Population-based cohort study  n=16,234  Median follow-up 15.0 years | Participants of the Health and Retirement Study, US (waves 2000–2016) aged ≥50 years who did not self-report their ethnicity as "other" | Dementia based on Langa–Weir classification containing proxy and self-reports and neuropsychological assessment | A multistate framework to simultaneously evaluate all risk factors was performed while accounting for interval censoring and the semi-competing risk of mortality | Dementia incidence rates higher for non-White groups |
| **Inter-personal Violence and Abuse** | | | | | | |
| Cations, 2022 ^92^ | Intimate partner violence.  Self-reported historical exposure was assessed with the question “Have you ever been in a violent relationship with a spouse/partner?”. More recent exposure with either two of the items “Being pushed, grabbed, shoved, kicked, or hit” and “Being forced to take part in unwanted sexual activity” in last 12 months. Classified into none, historical, current, or both | Population-based cohort study  n=12,085  Up to 21 years of follow-up | Australian women aged 70-75 enrolled in the Australian Longitudinal Study of Women’s Health | Dementia status was self-reported or obtained from linked secondary care, social care, prescription, or mortality data | Fine-Gray proportional hazards modelling with death as a competing risk adjusting for age, SES, country of birth, education, smoking, alcohol, physical activity, social support, mental health index, and physical health index | Null findings. Any history of intimate partner violence compared to no history: HR 1.02 (0.89, 1.17) |
| Koga, 2022 ^93^ | Elder abuse.  Measured across 3 self-reported domains: (1) physical abuse (‘In the past year, did you experience physical violence such as being hit, kicked…?’; (2) psychological abuse (‘In the past year, did you experience an act that harmed your self-esteem such as verbal abuse, cutting remarks…’); and (3)  financial abuse (‘Does anyone, including your family members, take or use your savings or pension benefits without your consent?’) | Population-based cohort study  n=12,236  Up to 6 years | Adults aged ≥65 years enrolled in the Japan Gerontological Evaluation Study, who were physically and cognitively independent | Dementia was assessed using the nationally standardized dementia scale proposed by the Ministry of Health, Labor and Welfare | Poisson regression analyses adjusted for sex, age, education, income, marital status, living arrangements, depressive symptoms, BMI, job type/status, alcohol, smoking, frequency of meeting friends, daily walking time, hypertension, stroke, DM, hearing, ADLs | Reporting financial abuse was associated with an increased risk of dementia: IRR 1.53 (1.09, 2.16). This increased risk was highest (IRR >2) in the first 500 days of follow-up but remained significant through later follow-up periods. Null association for psychological abuse, and a non-significant increase (IRR 1.53) for physical abuse |
| Leung, 2006 ^94^ | Spousal abuse.  Defined as having been struck in the head on ≥5 occasions with loss of consciousness (any time duration) on ≥2 of these occasions, self-reported and substantiated by one or more children | Case-control study  n=120 | Consecutive women referred to a Memory Disorders Clinic diagnosed with AD, compared to a group with ischaemic neurological disease, and another with epilepsy | AD was defined according to National Institute of Neurological and Communicative Disorders and Stroke criteria for probable AD | Fisher’s exact tests | No significant differences between cases and controls, though this was designed as a pilot study and was underpowered |
| Tani, 2020 ^95^ | ACEs.  Self-reported score of 0, 1, 2, or >3 of the following occurring before the age of 18: parental death, parental divorce, parental mental illness, family violence, physical abuse, psychological neglect, and psychological abuse | Population-based cohort study  n=17,412  Mean follow-up 3.2 years from data collection (but exposure from childhood) | Japanese adults aged ≥65 years enrolled in the Japan Gerontological Evaluation Study | Dementia incidence was ascertained by linking the participants to the standardized in-home assessment and medical examination conducted under Japan’s public long-term care insurance registry | Cox regression models were used adjusting for age, sex, childhood economic hardship, childhood height, education, hypertension, DM, stroke, heart disease, adult annual income, adult longest occupation, marital status, frequency of meeting friends, social participation, employment status, smoking, BMI, depressive symptoms, and hearing loss | Participants who experienced 3 or more ACEs had a greater risk of developing dementia compared with those who grew up without any ACEs: HR 1.78 (1.15, 2.75). Null associations for those who experienced 1 or 2 ACEs (compared to 0) |
| Tani, 2021a ^96^ | ACEs and individual-level social capital.  ACEs measured as per Tani, 2020 above. Individual-level social capital measured with three self-reported items (community trust, reciprocity, and attachment) and the overall social capital score was categorized as low (bottom 10%), middle, or high top 10%) | Population-based cohort study  n=16,821  Mean follow-up 3.2 years from data collection (but exposure from childhood) | Japanese adults aged ≥65 years enrolled in the Japan Gerontological Evaluation Study | Dementia incidence was ascertained by linking the participants to the standardized in-home assessment and medical examination conducted under Japan’s public long-term care insurance registry | Cox proportional hazards models adjusted for age, sex, childhood environment (economic hardship, height, and education), adult sociodemographic characteristics (income, longest-held occupation), smoking, BMI, depressive symptoms, hypertension, DM, stroke, heart disease, hearing loss, structural social capital (marital status, frequency of meeting friends, social participation, and employment status), and happiness | ACEs and lower social capital were associated with an increased risk of dementia. Stratification by social capital score showed that participants with low social capital and ≥3 adverse childhood experiences (versus none) had an increased dementia risk: HR 2.80 (1.44, 5.45), while no association was observed for those with medium social capital and ≥3 adverse childhood experiences: HR 1.19 (0.58, 2.43). No dementia cases were observed among those with ≥3 adverse childhood experiences and high social capital |
| **Social Capital/Social Cohesion** | | | | | | |
| Choi, 2024 ^66^ | Objective social isolation was assessed using the Steptoe Social Isolation index and subjective social isolation with the shortened Revised UCLA Loneliness Scale | Population-based cohort study  n=9,251  Median 9 years of follow-up | US adults aged >55 enrolled in the Health and Retirement Study | Dementia was assessed using the Langa–Weir classification containing proxy and self-reports and neuropsychological assessment | Separate Cox proportional hazards models adjusted for age, sex, race/ethnicity, education, household wealth, employment status, cardiometabolic abnormalities, and cognitive status, including inverse probability of treatment weighting, as well as imputation and proxy status | Both objective HR=1.14 (1.06, 1.23) and subjective social isolation were associ­ated with increased dementia risk HR=1.10 (1.05, 1.14) |
| Fujihara, 2023 ^97^ | Social capital.  Assessed using 3 self-reported domains: civic participation (e.g. engagement in volunteer groups), social cohesion (e.g. ‘do you think that people living in your area can be trusted in general?’), and reciprocity (e.g. ‘do you have someone who listens to your concerns and complaints?’ (and do you do this for others?)). Summed for individuals and at the community-level | Population-based cohort study  n=35,921  Mean follow-up 7.6 years | Adults aged ≥65 years enrolled in the Japan Gerontological Evaluation Study, who were physically and cognitively independent | Dementia onset was assessed using a standardized in-home assessment under the Japanese long-term care insurance scheme | Two-level multilevel survival analysis stratified by sex, adjusted for the community- and individual-level social capital and cross-level interaction terms, community-level covariates (population density, sidewalk coverage) and individual-level covariates (sex, age, marital status, living arrangement, education, income, employment status, alcohol, smoking, stroke, hypertension, DM, hearing loss, depression, and daily walking time) | At the individual level, civic participation was associated with a lower incidence of dementia in both males: HR 0.84 (0.77, 0.92); and females: HR 0.78 (0.73, 0.84). But null associations for social cohesion and reciprocity. At the community level, only civic participation (HR: 0.96) and social cohesion (HR: 0.93) were associated with a lower risk, but only for females. Other analyses null |
| Wong, 2022 ^98^ | Social cohesion and neighbourhood disorder.  Social cohesion composite score based on: self-reported knowing ("People in this community know each other very well"), helping ("People in this community are willing to help each other"), and trusting ("People in this community can be trusted"). Neighbourhood disorder recorded by study interviewers, how much e.g. litter, broken glass, graffiti, deserted dwellings can you see from the front of the person’s house | Population-based cohort study  n=5,181  Up to 9 years of follow-up | USA Medicare beneficiaries aged ≥65 years enrolled in the National Health and Aging Trends Study | Dementia diagnosis was derived from the study's algorithm that uses three cognitive measures: 1) Dementia Screening Interview, 2) cognitive tests, and 3) self-report of an AD or dementia diagnosis by a doctor | Cox regression analyses utilising survey weights and adjusted for age, sex, education, income, marital status, household size, metropolitan residence, self-rated health, BMI, ADLs, proxy respondent, major depressive disorder, generalized anxiety disorder, MI, hypertension, DM, stroke | Higher neighbourhood physical disorder was associated with increased dementia risk: HR 1.11 (1.01, 1.23). Compared to White participants living in an area of higher physical disorder, Hispanic participants had a higher dementia risk: HR 0.62 (0.49;0.79), with a smaller (HR 0.85) and non-significant finding for Black participants. No associations were reported between social cohesion and dementia risk |
| **Gender and Sexual Identity** | | | | | | |
| Guo, 2022 ^99^ | Transgender identity.  Transgender and cisgender adults were identified using a computable phenotyping algorithm drawing data from the gender field of clinical records, and ICD-9 and -10 codes related to transgender status | Clinic-based cohort study  n=37,069 | US adults with electronic health data available from the ‘OneFlorida’ clinical research network database between 2012-2020 | Dementia was assessed using ICD-9 and -10 codes as per the Centers for Medicare and Medicaid Services’ Chronic Conditions Data Warehouse | 10 male and 10 female cisgender controls matched to each transgender adult (matched on age and ethnicity). Overall and age-stratified prevalence rates calculated with a Chi-squared test for significance | Overall dementia prevalence was higher for transgender adults than cisgender controls (1.7% vs 0.8%, p<0.001). It was also higher in adults aged 18-49 (1.1% vs 0.3%, p<0.001) and in the >50 group (3.5% vs 2.2%) but this was not significant (p=0.067) which the authors ascribe to insufficient power (mean age 39.2) |
| Hanes, 2023 ^100^ | Same-sex relationships.  Participants' and partners' sex/gender was assessed at baseline with two responses: male and female. Categorised as same-sex relationship if they had a same sex/gender partner at any timepoint | Population-based cohort study  n=26,344  Up to 10 years follow-up | US adults aged ≥50 enrolled in the Health and Retirement Study from 1998-2018 | Dementia based on Langa–Weir classification containing proxy and self-reports and neuropsychological assessment | Multiple regression analyses compared the ages at which participants in same-sex relationships and different-sex relationships first reported AD or dementia diagnoses adjusted for baseline cognition, sex/gender, education, ethnicity, wealth, and self-rated health | Participants in same-sex relationships had self-reported dementia at a younger age on average (p<.001) (number of years difference not reported). Similar findings based on incident cognitive impairment indicating probable dementia (p<0.001) |
| Perales-Puchalt, 2019 ^101^ | Same-sex relationships.  Self-reported sex of participant and partner. Categorised as same-sex relationship if they had a same sex/gender partner at any timepoint | Clinic-based cohort study  n=5,057  Up to 12 years follow-up | Individuals registered on the US National Alzheimer’s Coordinating Center database, aged ≥55 years and with normal cognition at baseline, who had a spouse, partner or companion as study partner at any assessment | Dementia diagnoses were made by a clinician or a consensus team according DSM criteria | Cox models, adjusted for clustering by study centre, age at baseline, sex, education, living alone, and APOE e4 | No significant difference in dementia risk was found between those in a same-sex and opposite-sex relationship: HR 1.21 (0.73, 2.00) |
| **Education** |  |  |  |  |  |  |
| Appel, 2022 ^45^ | Occupation and education.  Longest held position (of at least 3 years) between age 30 and 55. Years of formal education (>12 years, 10-12 years, <10 years) | Registry-based cohort study  n=1,210,720  Mean follow-up 10.5 years | Entire Danish population, excluding those who emigrated, those unemployed, and those assisting a self-employed spouse | Incident dementia defined by ICD-8 codes and anti-dementia medication ascertained from record linkage with in-patient, prescribing, research, and mortality datasets | Aalen’s additive hazards model adjusted for age, birth cohort, sex, marital status, cognitive ability (at 18), and Charlson Comorbidity Index | Each decrease in educational attainment and occupation-based SEP level was associated with higher dementia risk in a dose–response manner (14.8 and 10.9 additional dementia cases per 100,000 PY, respectively). Higher occupation-based SEP partly mitigated the higher dementia risk associated with lower educational attainment |
| Fortinsky, 2023 ^81^ | Ethnicity and educational attainment.  Ethnicity: self-report classified as non-Hispanic White, non-Hispanic White, non-Hispanic other, Hispanic. Educational attainment: primary, secondary, or tertiary education | Cross-sectional study  n=3,520 | Adults aged ≥65 years receiving home- and community-based services in Connecticut, USA in 2019 | Diagnosed dementia or Cognitive Performance Scale 2 score ≥4 | Generalized multivariate logistic regression adjusted for participant age group, gender, education, race/ethnicity, depression, Charlson comorbidity index (excluding depression), and Social Vulnerability Index category. | No significant differences in dementia risk by ethnicity. Higher likelihood of dementia for those with only primary education (compared to tertiary): OR 1.82 (1.45, 2.28) |
| Hayes-Larson, 2023 ^83^ | Ethnicity, migrant status, and education.  Self-reported Asian American ethnic group (Chinese, Filipino, Japanese), US-born or foreign-born status, and educational attainment (binary: college degree or not) | Population-based cohort study  n=14,749  Mean follow-up 9.9 years | Adults aged 60-90 members of the Kaiser Permanente Northern California health care delivery system | Incident dementia diagnosis using ICD-9 and ICD-10 codes in electronic healthcare records | Cox proportional hazards and Aalen additive hazards models with age as timescale, adjusted for sex, and interaction of education with nativity | College education was associated with a lower dementia risk. This association was stronger and only statistically significant in the foreign-born strata. Within these strata, college education was associated with a reduced dementia risk for Chinese and Filipino groups but null associations for the Japanese group |
| Hudomiet, 2022 ^70^ | Lifetime earnings, educational attainment, ethnicity, migrant status.  Lifetime earnings based on an individual’s 35 highest years of earnings. Self-reported ethnicities: non-Hispanic White, non-Hispanic Black, Hispanic, non-Hispanic other | Population-based cohort study  n=21,422  Up to 16 years of follow-up | US adults aged ≥65 enrolled in the Health and Retirement Study | Trends in dementia are primarily based on wave-to-wave differences in the averages of individuals’ performance on cognitive tests, but using an algorithm calibrated using a subgroup with  clinical diagnosis of cognitive tests to measure dementia | Dementia prevalence was estimated by a fitted linear time trend adjusting for the distributions of age, education, income, race and ethnicity, foreign-born status, marital status, hypertension, DM, stroke, and heart problems | Dementia prevalence rates dropped during the period of interest (2000-2016). Improvements in educational attainment accounted for half of this decrease. Prevalence of dementia tended to be higher among those with low life course earnings, lower education, ethnic minority individuals, and for those not born in the US. These differences generally narrowed over time |
| Hyun, 2022 ^46^ | Occupational complexity and education.  Measures of occupational complexity were harmonized across countries using the International Standard Classification of Occupations (ISCO)-08. Educational attainment (highest qualification achieved) | Harmonised multi-community-based cohort study  n=10,195  Up to 6.4 years of follow-up (from data capture, longer from actual exposure) | Participants aged 58-103 years from seven studies included in the COSMIC collaboration representing 6 countries over 4 continents | Dementia diagnosis using DSM-IV criteria or Clinical Dementia Rating scale | Accelerated failure time model with a Weibull distribution controlled for sex and baseline age, late-life cardiovascular comorbidities, education, and APOE ε4 status. With causal mediation analysis | The meta-analytic estimate indicated that high occupational complexity, compared to low complexity, was associated with a 19% (5, 33) increase in dementia-free survival. 28% of the effect of education on dementia risk was mediated by occupational complexity |
| Perales-Puchalt, 2019 ^101^ | Same-sex relationships.  Self-reported sex of participant and partner. Categorised as same-sex relationship if they had a same sex/gender partner at any timepoint | Clinic-based cohort study  n=5,057  Up to 12 years follow-up | Individuals registered on the US National Alzheimer’s Coordinating Centre database, aged ≥55 years and with normal cognition at baseline, who had a spouse, partner or companion as study partner at any assessment | Dementia diagnoses were made by a clinician or a consensus team according DSM criteria | Cox models, adjusted for clustering by study centre, age at baseline, sex, education, living alone, and APOE e4 | No significant difference in dementia risk was found between those in a same-sex and opposite-sex relationship: HR 1.21 (0.73, 2.00) |
| SDOH = Social determinant of health. BMI = Body mass index. SES = Socioeconomic status. OR = Odds ratio. 95% CI = 95% Confidence interval. m = metre. Km = Kilometre. GIS = Geographic Information System. DM = Diabetes mellitus. ADL = Activities of Daily Living. HR = Hazard ratio. AD = Alzheimer’s disease. APOE = Apolipoprotein E. PM = Particulate matter. IQR = Interquartile range. ICD = International Classification of Disease. PD = Parkinson’s disease. DSM = Diagnostic and statistical manual. MI = Myocardial infarction. HSMS = High stroke mortality state. CVD = cardiovascular disease. TBI = Traumatic brain injury. PTSD = posttraumatic stress disorder. RR = Relative risk. COSMIC = Cohort Studies of Memory in an International Consortium. GWAS = Genome wide association studies. IRR = Incidence rate ratio. GSEM = Generalized structural equation modelling. ACEs = Adverse Childhood Experiences | | | | | | |

**References**

1. McMichael AJ, McGuinness B, Lee J, Minh HV, Woodside JV, McEvoy CT. Food insecurity and brain health in adults: A systematic review. *Critical reviews in food science and nutrition*. 2022;62(31):8728-8743. doi:10.1080/10408398.2021.1932721

2. Zhao Y-L, Qu Y, Ou Y-N, Zhang Y-R, Tan L, Yu J-T. Environmental factors and risks of cognitive impairment and dementia: A systematic review and meta-analysis. *Ageing research reviews*. 2021;72:101504. doi:10.1016/j.arr.2021.101504

3. Killin LOJ, Starr JM, Shiue IJ, Russ TC. Environmental risk factors for dementia: a systematic review. *BMC geriatrics*. 2016;16(1):175. doi:10.1186/s12877-016-0342-y

4. Livingston G, Huntley J, Sommerlad A, et al. Dementia prevention, intervention, and care: 2020 report of the Lancet Commission. *The Lancet*. 2020;396(10248):413-446. doi:10.1016/S0140-6736(20)30367-6

5. Livingston G, Huntley J, Liu KY, et al. Dementia prevention, intervention, and care: 2024 report of the Lancet standing Commission. *The Lancet*. 2024;404(10452):572-628. doi:10.1016/ S0140-6736(24)01296-0

6. Chen X, Lee C, Huang H. Neighborhood built environment associated with cognition and dementia risk among older adults: A systematic literature review. *Social science & medicine (1982)*. 2022;292:114560. doi:10.1016/j.socscimed.2021.114560

7. Huang L, Zhang Y, Wang Y, Lan Y. Relationship Between Chronic Noise Exposure, Cognitive Impairment, and Degenerative Dementia: Update on the Experimental and Epidemiological Evidence and Prospects for Further Research. *Journal of Alzheimer's disease : JAD*. 2021;79(4):1409-1427. doi:10.3233/JAD-201037

8. Clark C, Crumpler C, Notley, Hilary. Evidence for Environmental Noise Effects on Health for the United Kingdom Policy Context: A Systematic Review of the Effects of Environmental Noise on Mental Health, Wellbeing, Quality of Life, Cancer, Dementia, Birth, Reproductive Outcomes, and Cognition. *International journal of environmental research and public health*. 2020;17(2)doi:10.3390/ijerph17020393

9. Yan D, Zhang Y, Liu L, Yan H. Pesticide exposure and risk of Alzheimer's disease: a systematic review and meta-analysis. *Scientific reports*. 2016;6:32222. doi:10.1038/srep32222

10. Jalilian H, Teshnizi SH, Roosli M, Neghab M. Occupational exposure to extremely low frequency magnetic fields and risk of Alzheimer disease: A systematic review and meta-analysis. *Neurotoxicology*. 2018;69:242-252. doi:10.1016/j.neuro.2017.12.005

11. Garcia AM, Sisternas A, Hoyos SP. Occupational exposure to extremely low frequency electric and magnetic fields and Alzheimer disease: a meta-analysis. *International journal of epidemiology*. 2008;37(2):329-40. Comment in: Int J Epidemiol. 2008 Apr;37(2):341-3 PMID: 18276625 [https://www.ncbi.nlm.nih.gov/pubmed/18276625]. doi:10.1093/ije/dym295

12. Babulal GM, Rani R, Adkins-Jackson P, Pearson AC, Williams MM. Associations between Homelessness and Alzheimer's Disease and Related Dementia: A Systematic Review. *Journal of applied gerontology : the official journal of the Southern Gerontological Society*. 2022;41(11):2404-2413. doi:https:10.1177/07334648221109747

13. Huang L-Y, Hu H-Y, Wang Z-T, et al. Association of Occupational Factors and Dementia or Cognitive Impairment: A Systematic Review and Meta-Analysis. *Journal of Alzheimer's disease : JAD*. 2020;78(1):217-227. doi:10.3233/JAD-200605

14. Gracia Rebled AC, Santabarbara Serrano J, Lopez Anton RLA, Tomas Aznar C, Marcos Aragues G. [Occupation and Risk of Cognitive Impairment and Dementia in People in over 55 Years: A Systematic Review, Spain]. *Ocupacion laboral y riesgo de deterioro cognitivo y demencia en personas mayores de 55 anos: una revision sistematica*. 2016;90:e1-e15.

15. Then FS, Luck T, Luppa M, et al. Systematic review of the effect of the psychosocial working environment on cognition and dementia. *Occupational and environmental medicine*. 2014;71(5):358-65. Comment in: Occup Environ Med. 2014 May;71(5):305-6 PMID: 24578488 [https://www.ncbi.nlm.nih.gov/pubmed/24578488]. doi:10.1136/oemed-2013-101760

16. Gao Y, Fu X, Hu H, et al. Impact of shift work on dementia: a systematic review and dose-response meta-analysis. *Public health*. 2023;223:80-86. doi:10.1016/j.puhe.2023.07.029

17. Hai Y, Xue Y, Wang Y-H. Does Long-Term Shift Work Increase the Risk of Dementia? A Systematic Review and Meta-Analysis. *American journal of Alzheimer's disease and other dementias*. 2022;37:15333175221141535. doi:10.1177/15333175221141535

18. Leso V, Caturano A, Vetrani I, Iavicoli I. Shift or night shift work and dementia risk: a systematic review. *European review for medical and pharmacological sciences*. 2021;25(1):222-232. doi:10.26355/eurrev_202101_24388

19. Livingston G, Sommerlad A, Orgeta V, et al. Dementia prevention, intervention, and care. *The Lancet*. 2017;390(10113):2673-2734. doi:10.1016/S0140-6736(17)31363-6

20. Bodryzlova Y, Kim A, Michaud X, André C, Bélanger E, Moullec G. Social class and the risk of dementia: A systematic review and meta-analysis of the prospective longitudinal studies. *Scandinavian Journal of Public Health*. 2022:14034948221110019. doi:10.1177/14034948221110019

21. Wang A-Y, Hu H-Y, Ou Y-N, et al. Socioeconomic status and risks of cognitive impairment and dementia: a systematic review and meta-analysis of 39 prospective studies. *The Journal of Prevention of Alzheimer's Disease*. 2023;10:83-94. doi:10.14283/jpad.2022.81

22. Fratiglioni L, Wang H-X. Brain reserve hypothesis in dementia. *Journal of Alzheimer's disease : JAD*. 2007;12(1):11-22.

23. Shiekh SI, Cadogan SL, Lin L-Y, Mathur R, Smeeth L, Warren-Gash C. Ethnic differences in dementia risk: a systematic review and meta-analysis. *Journal of Alzheimer's Disease*. 2021;80(1):337-355. doi:10.3233/JAD-201209

24. Mehta KM, Yeo GW. Systematic review of dementia prevalence and incidence in United States race/ethnic populations. *Alzheimer's & dementia : the journal of the Alzheimer's Association*. 2017;13(1):72-83. doi:10.1016/j.jalz.2016.06.2360

25. Venketasubramanian N, Sahadevan S, Kua E, Chen C, Ng T-P. Interethnic differences in dementia epidemiology: global and Asia-Pacific perspectives. *Dementia and geriatric cognitive disorders*. 2011;30(6):492-498. doi:10.1159/000321675

26. Selten J-P, Termorshuizen F, van Sonsbeek M, Bogers J, Schmand B. Migration and dementia: a meta-analysis of epidemiological studies in Europe. *Psychological medicine*. 2021;51(11):1838-1845. doi:10.1017/S0033291720000586

27. Qian H, Khadka A, Martinez SM, et al. Food Insecurity, Memory, and Dementia Among US Adults Aged 50 Years and Older. *JAMA Network Open*. 2023;6(11):e2344186-e2344186. doi:10.1001/jamanetworkopen.2023.44186

28. Tani Y, Suzuki N, Fujiwara T, Hanazato M, Kondo K. Neighborhood food environment and dementia incidence: the Japan gerontological evaluation study cohort survey. *American journal of preventive medicine*. 2019;56(3):383-392. doi:10.1016/j.amepre.2018.10.028

29. Brown S, Aitken W, Lombard J, et al. Longitudinal Impacts of Precision Greenness on Alzheimer’s Disease. *The Journal of Prevention of Alzheimer's Disease*. 2024:1-11. doi:10.14283/jpad.2024.38

30. Godina SL, Rosso AL, Hirsch JA, et al. Neighborhood greenspace and cognition: The cardiovascular health study. *Health & place*. 2023;79:102960. doi:10.1016/j.healthplace.2022.102960

31. Hu H-Y, Ma Y-H, Deng Y-T, et al. Residential greenness and risk of incident dementia: a prospective study of 375,342 participants. *Environmental Research*. 2023;216:114703. doi:10.1016/j.envres.2022.114703

32. Klompmaker JO, Laden F, Browning MH, et al. Associations of greenness, parks, and blue space with neurodegenerative disease hospitalizations among older US adults. *JAMA Network Open*. 2022;5(12):e2247664-e2247664. doi:10.1001/jamanetworkopen.2022.47664

33. Rodriguez-Loureiro L, Gadeyne S, Bauwelinck M, Lefebvre W, Vanpoucke C, Casas L. Long-term exposure to residential greenness and neurodegenerative disease mortality among older adults: a 13-year follow-up cohort study. *Environmental health*. 2022;21(1):49. doi:10.1186/s12940-022-00863-x

34. Slawsky ED, Hajat A, Rhew IC, et al. Neighborhood greenspace exposure as a protective factor in dementia risk among US adults 75 years or older: a cohort study. *Environmental Health*. 2022;21(1):14. doi:10.1186/s12940-022-00830-6

35. Tani Y, Hanazato M, Fujiwara T, Suzuki N, Kondo K. Neighborhood sidewalk environment and incidence of dementia in older Japanese adults: the Japan gerontological evaluation study cohort. *American journal of epidemiology*. 2021;190(7):1270-1280. doi:10.1093/aje/kwab043

36. Cantuaria ML, Waldorff FB, Wermuth L, et al. Residential exposure to transportation noise in Denmark and incidence of dementia: national cohort study. *British Medical Journal*. 2021;374. doi:10.1136/bmj.n1954

37. Gilsanz P, Mayeda ER, Glymour MM, Quesenberry CP, Whitmer RA. Association between birth in a high stroke mortality state, race, and risk of dementia. *JAMA neurology*. 2017;74(9):1056-1062. doi:10.1001/jamaneurol.2017.1553

38. Li L, Cheng G-R, Liu D, et al. The Hubei Memory and Aging Cohort Study: study design, baseline characteristics, and prevalence of cognitive impairments. *Journal of Alzheimer's Disease*. 2022;85(2):561-571. doi:10.3233/JAD-215129

39. Li M, Ma C, Wu C. Association between solid cooking fuel use and dementia in older Chinese adults: the mediating effect of depression. *International Journal of Environmental Health Research*. 2024;34(2):779-791. doi:10.1080/09603123.2023.2167950

40. Martinez S, Yaffe K, Li Y, Byers AL, Peltz CB, Barnes DE. Agent Orange exposure and dementia diagnosis in US veterans of the Vietnam era. *JAMA neurology*. 2021;78(4):473-477. doi:10.1001/jamaneurol.2020.5011

41. Zeng X, Berriault C, Arrandale VH, DeBono NL, Harris MA, Demers PA. Radon exposure and risk of neurodegenerative diseases among male miners in Ontario, Canada: a cohort study. *American Journal of Industrial Medicine*. 2023;66(2):132-141. doi:10.1002/ajim.23449

42. Zeng X, MacLeod J, Berriault C, et al. Aluminum dust exposure and risk of neurodegenerative diseases in a cohort of male miners in Ontario, Canada. *Scandinavian journal of work, environment & health*. 2021;47(7):531-539. doi:10.5271/sjweh.3974

43. Zhou S, Wang K. Childhood secondhand smoke exposure and risk of dementia, Alzheimer’s disease and stroke in adulthood: A prospective cohort study. *The Journal of Prevention of Alzheimer's Disease*. 2021;8:345-350. doi:10.14283/jpad.2021.10

44. Roncarati JS, DeVone F, Halladay C, Tsai J, Jutkowitz E. Risk of dementia among veterans experiencing homelessness and housing instability. *Journal of the American Geriatrics Society*. 2024;72(2):382-389. doi:10.1111/jgs.18680

45. Appel AM, Brønnum-Hansen H, Garde AH, et al. Socioeconomic position and late-onset dementia: a nationwide register-based study. *Journal of aging and health*. 2022;34(2):184-195. doi:10.1177/0898264321103720

46. Hyun J, Hall CB, Katz MJ, et al. Education, occupational complexity, and incident dementia: a COSMIC collaborative cohort study. *Journal of Alzheimer’s Disease*. 2022;85(1):179-196. doi:10.3233/JAD-21062

47. Hyun J, Hall CB, Sliwinski MJ, et al. Effect of mentally challenging occupations on incident dementia differs between African Americans and non-Hispanic Whites. *Journal of Alzheimer's Disease*. 2020;75(4):1405-1416. doi:10.3233/JAD-191222

48. Ishtiak-Ahmed K, Hansen ÅM, Mortensen EL, et al. Prolonged or serious conflicts at work and incident dementia: a 23-year follow-up of the Copenhagen City Heart Study. *International Archives of Occupational and Environmental Health*. 2019;92:165-173. doi:10.1007/s00420-018-1365-9

49. Ishtiak-Ahmed K, Hansen ÅM, Garde AH, et al. Social relations at work and incident dementia: 29-years’ follow-up of the Copenhagen male study. *Journal of Occupational and Environmental Medicine*. 2018;60(1):12-18. doi:10.1097/JOM.0000000000001158

50. Kivimäki M, Walker KA, Pentti J, et al. Cognitive stimulation in the workplace, plasma proteins, and risk of dementia: three analyses of population cohort studies. *British Medical Journal*. 2021;374. doi:10.1136/bmj.n1804

51. Ko H, Kim S, Kim K, et al. Genome-wide association study of occupational attainment as a proxy for cognitive reserve. *Brain*. 2022;145(4):1436-1448. doi:10.1093/brain/awab351

52. Martin‐Bassols N, de New SC, Johnston DW, Shields MA. Cognitive activity at work and the risk of dementia. *Health Economics*. 2023;32(7):1561-1580. doi:10.1002/hec.4679

53. Nabe‐Nielsen K, Holtermann A, Gyntelberg F, et al. The effect of occupational physical activity on dementia: results from the Copenhagen Male Study. *Scandinavian journal of medicine & science in sports*. 2021;31(2):446-455. doi:10.1111/sms.13846

54. Raggi M, Dugravot A, Valeri L, et al. Contribution of smoking towards the association between socioeconomic position and dementia: 32-year follow-up of the Whitehall II prospective cohort study. *The Lancet Regional Health–Europe*. 2022;23doi:10.1016/j.lanepe.2022.100516

55. Sundström A, Rönnlund M, Josefsson M. A nationwide Swedish study of age at retirement and dementia risk. *International journal of geriatric psychiatry*. 2020;35(10):1243-1249. doi:10.1002/gps.5363

56. Sundström A, Sörman DE, Hansson P, Ljungberg JK, Adolfsson R. Mental demands at work and risk of dementia. *Journal of Alzheimer's disease*. 2020;74(3):735-740. doi:10.3233/JAD-190920

57. Zhao J, Li K, Liao X. Working status and risk of Alzheimer's disease: A Mendelian randomization study. *Brain and Behavior*. 2023;13(1):e2834. doi:10.1002/brb3.2834

58. Liao H, Pan D, Deng Z, et al. Association of shift work with incident dementia: a community-based cohort study. *BMC medicine*. 2022;20(1):484. doi:10.1186/s12916-022-02667-9

59. Ling Y, Yuan S, Huang X, et al. The association of night shift work with the risk of all-cause dementia and Alzheimer's disease: A longitudinal study of 245,570 UK biobank participants. *Journal of Neurology*. 2023;270(7):3499-3510. doi:10.1007/s00415-023-11672-8

60. Ren J-J, Zhang P-D, Li Z-H, et al. Association of night shifts and lifestyle risks with incident dementia. *The Journals of Gerontology: Series A*. 2023;78(9):1725-1732. doi:10.1093/gerona/glad116

61. Arora K, Xu L, Bhagianadh D. Dementia and cognitive decline in older adulthood: are agricultural workers at greater risk? *The Journals of Gerontology: Series B*. 2021;76(8):1629-1643. doi:10.1093/geronb/gbab005

62. Power MC, Murphy AE, Gianattasio KZ, et al. Association of military employment with late-life cognitive decline and dementia: a population-based prospective cohort study. *Military medicine*. 2023;188(5-6):e1132-e1139. doi:10.1093/milmed/usab413

63. Jain S, Murphy TE, O’Leary JR, Leo-Summers L, Ferrante LE. Association between socioeconomic disadvantage and decline in function, cognition, and mental health after critical illness among older adults: a cohort study. *Annals of internal medicine*. 2022;175(5):644-655. doi:10.7326/M21-3086

64. Keohane LM, Nikpay S, Braun K, et al. Association of race and income with incident diagnosis of Alzheimer’s disease and related dementias among Black and White older adults. *Journal of Applied Gerontology*. 2023;42(5):898-908. doi:10.1177/07334648221142851

65. Becerril A, Pfoh ER, Hashmi AZ, et al. Racial, ethnic and neighborhood socioeconomic differences in incidence of dementia: a regional retrospective cohort study. *Journal of the American Geriatrics Society*. 2023;71(8):2406-2418. doi:10.1111/jgs.18322

66. Choi EY, Cho G, Chang VW. Neighborhood Social Environment and Dementia: The Mediating Role of Social Isolation. *The Journals of Gerontology, Series B: Psychological Sciences and Social Sciences*. 2024;79(4):gbad199. doi:10.1093/geronb/gbad199

67. Dai J, Xu Y, Wang T, Zeng P. Exploring the relationship between socioeconomic deprivation index and Alzheimer's disease using summary-level data: From genetic correlation to causality. *Progress in Neuro-Psychopharmacology and Biological Psychiatry*. 2023;123:110700. doi:10.1016/j.pnpbp.2022.110700

68. Park YS, Joo HJ, Jang YS, Jeon H, Park E-C, Shin J. Socioeconomic Status and Dementia Risk Among Intensive Care Unit Survivors: Using National Health Insurance Cohort in Korea. *Journal of Alzheimer's Disease*. 2024;91(1):273-281. doi:10.3233/JAD-230715

69. Geraets AF, Leist AK. Sex/gender and socioeconomic differences in modifiable risk factors for dementia. *Scientific Reports*. 2023;13(1):80. doi:10.1038/s41598-022-27368-4

70. Hudomiet P, Hurd MD, Rohwedder S. Trends in inequalities in the prevalence of dementia in the United States. *Proceedings of the National Academy of Sciences*. 2022;119(46):e2212205119. doi:10.1073/pnas.2212205119

71. Klee M, Leist AK, Veldsman M, Ranson JM, Llewellyn DJ. Socioeconomic Deprivation, Genetic Risk, and Incident Dementia. *American Journal of Preventive Medicine*. 2023;64(5):621-630. doi:10.1016/j.amepre.2023.01.012

72. Korhonen K, Leinonen T, Tarkiainen L, Einiö E, Martikainen P. Childhood socio-economic circumstances and dementia: prospective register-based cohort study of adulthood socio-economic and cardiovascular health mediators. *International Journal of Epidemiology*. 2023;52(2):523-535. doi:10.1093/ije/dyac205

73. Lai KY, Webster C, Kumari S, Gallacher JE, Sarkar C. The associations of socioeconomic status with incident dementia and Alzheimer’s disease are modified by leucocyte telomere length: A population-based cohort study. *Scientific reports*. 2023;13(1):6163. doi:10.1038/s41598-023-32974-x

74. Li R, Li R, Xie J, et al. Associations of socioeconomic status and healthy lifestyle with incident early-onset and late-onset dementia: a prospective cohort study. *The Lancet Healthy Longevity*. 2023;4(12):e693-e702. doi:10.1016/S2666-7568(23)00211-8

75. Ou Y-N, Zhang Y-B, Li Y-Z, et al. Socioeconomic status, lifestyle and risk of incident dementia: a prospective cohort study of 276730 participants. *Geroscience*. 2024;46(2):2265-2279. doi:10.1007/s11357-023-00994-0

76. Weiss J. Contribution of socioeconomic, lifestyle, and medical risk factors to disparities in dementia and mortality. *SSM-Population Health*. 2021;16:100979. doi:10.1016/j.ssmph.2021.100979

77. Beydoun MA, Beydoun HA, Banerjee S, Weiss J, Evans MK, Zonderman AB. Pathways explaining racial/ethnic and socio-economic disparities in incident all-cause dementia among older US adults across income groups. *Translational Psychiatry*. 2022;12(1):478. doi:10.1038/s41398-022-02243-y

78. Bonnechère B, Liu J, Thompson A, Amin N, van Duijn C. Does ethnicity influence dementia, stroke and mortality risk? Evidence from the UK Biobank. *Frontiers in Public Health*. 2023;11:1111321. doi:10.3389/fpubh.2023.1111321

79. Cha H, Thomas PA, Umberson D. Sibling deaths, racial/ethnic disadvantage, and dementia in later life. *The Journals of Gerontology: Series B*. 2022;77(8):1539-1549. doi:10.1093/geronb/gbab202

80. Cheung G, To E, Rivera-Rodriguez C, et al. Dementia prevalence estimation among the main ethnic groups in New Zealand: a population-based descriptive study of routinely collected health data. *BMJ open*. 2022;12(9):e062304. doi:10.1136/bmjopen-2022-062304

81. Fortinsky RH, Robison J, Steffens DC, Grady J, Migneault D, Wakefield D. Association of race, ethnicity, education, and neighborhood context with dementia prevalence and cognitive impairment severity among older adults receiving medicaid-funded home and community-based services. *The American Journal of Geriatric Psychiatry*. 2023;31(4):241-251. doi:10.1016/j.jagp.2022.12.001

82. Garcia MA, Ortiz K, Arévalo SP, et al. Age of migration and cognitive function among older Latinos in the United States. *Journal of Alzheimer's Disease*. 2020;76(4):1493-1511. doi:10.3233/JAD-191296

83. Hayes-Larson E, Ikesu R, Fong J, et al. Association of education with dementia incidence stratified by ethnicity and nativity in a cohort of older Asian American individuals. *JAMA network open*. 2023;6(3):e231661-e231661. doi:10.1001/jamanetworkopen.2023.1661

84. Higgins Tejera C, Ware EB, Kobayashi LC, et al. Decomposing interaction and mediating effects of race/ethnicity and circulating blood levels of cystatin C on cognitive status in the United States health and retirement study. *Frontiers in Human Neuroscience*. 2023;17:1052435. doi:10.3389/fnhum.2023.1052435

85. Kornblith E, Bahorik A, Boscardin WJ, Xia F, Barnes DE, Yaffe K. Association of race and ethnicity with incidence of dementia among older adults. *JAMA*. 2022;327(15):1488-1495. doi:10.1001/jama.2022.3550

86. Li JM, Boustani MA, French DD. Social determinants of health in community-dwelling dementia patients aged 65 and over: analysis of the 2019 national health interview survey. *Gerontology and Geriatric Medicine*. 2023;9:23337214231190244. doi:10.1177/23337214231190244

87. Lim U, Wang S, Park SY, et al. Risk of Alzheimer's disease and related dementia by sex and race/ethnicity: The Multiethnic Cohort Study. *Alzheimer's & Dementia*. 2022;18(9):1625-1634. doi:10.1002/alz.12528

88. Mukadam N, Marston L, Lewis G, Livingston G. Risk factors, ethnicity and dementia: A UK Biobank prospective cohort study of White, South Asian and Black participants. *Plos one*. 2022;17(10):e0275309. doi:10.1371/journal.pone.0275309

89. Mukadam N, Marston L, Lewis G, Mathur R, Rait G, Livingston G. Incidence, age at diagnosis and survival with dementia across ethnic groups in England: A longitudinal study using electronic health records. *Alzheimer's & Dementia*. 2023;19(4):1300-1307. doi:10.1002/alz.12774

90. Pohl DJ, Seblova D, Avila JF, et al. Relationship between residential segregation, later-life cognition, and incident dementia across race/ethnicity. *International Journal of Environmental Research and Public Health*. 2021;18(21):11233. doi:10.3390/ijerph182111233

91. Younan D, Wang X, Gruenewald T, et al. Racial/ethnic disparities in Alzheimer’s disease risk: role of exposure to ambient fine particles. *The Journals of Gerontology: Series A*. 2022;77(5):977-985. doi:10.1093/gerona/glab231

92. Cations M, Keage HA, Laver KE, Byles J, Loxton D. Intimate partner violence and risk for mortality and incident dementia in older women. *Journal of interpersonal violence*. 2022;37(5-6):NP2605-NP2625. doi:10.1177/0886260520943712

93. Koga C, Tsuji T, Hanazato M, Takasugi T, Kondo K. Types of elder abuse and Dementia onset among older adults in Japan: A 6-year longitudinal study from the Japan Gerontological Evaluation Study. *Archives of gerontology and geriatrics*. 2022;100:104656. doi:10.1016/j.archger.2022.104656

94. Leung F-H, Thompson K, Weaver DF. Evaluating spousal abuse as a potential risk factor for Alzheimer’s disease: Rationale, needs and challenges. *Neuroepidemiology*. 2006;27(1):13-16. doi:10.1159/000093894

95. Tani Y, Fujiwara T, Kondo K. Association between adverse childhood experiences and dementia in older Japanese adults. *JAMA Network Open*. 2020;3(2):e1920740-e1920740. doi:10.1001/jamanetworkopen.2019.20740

96. Tani Y, Fujiwara T, Kondo K. Adverse childhood experiences and dementia: Interactions with social capital in the Japan Gerontological Evaluation Study Cohort. *American journal of preventive medicine*. 2021;61(2):225-234. doi:10.1016/j.amepre.2021.01.045

97. Fujihara S, Tsuji T, Nakagomi A, et al. Association of community-level social capital with dementia: A multilevel nine-year longitudinal study using data from the Japan Gerontological Evaluation Study. *Social Science & Medicine*. 2023;338:116316. doi:10.1016/j.socscimed.2023.116316

98. Wong R, Wang Y. Role of neighborhood physical disorder and social cohesion on racial and ethnic disparities in dementia risk. *Journal of Aging and Health*. 2022;34(9-10):1178-1187. doi:10.1177/08982643221101352

99. Guo Y, Li Q, Yang X, et al. Prevalence of Alzheimer’s and related dementia diseases and risk factors among transgender adults, Florida, 2012‒2020. *American Journal of Public Health*. 2022;112(5):754-757. doi:10.2105/AJPH.2022.306720

100. Hanes DW, Clouston SA. Cognitive aging in same-and different-sex relationships: Comparing age of diagnosis and rate of cognitive decline in the health and retirement study. *Gerontology*. 2023;69(3):356-369. doi:10.1159/000526922

101. Perales‐Puchalt J, Gauthreaux K, Flatt J, et al. Risk of dementia and mild cognitive impairment among older adults in same‐sex relationships. *International Journal of Geriatric Psychiatry*. 2019;34(6):828-835. doi:10.1002/gps.5092
